# Enhancing Cultural Humility: Addressing Mental Health Disparities in AANHPI Communities

**DOI:** 10.15766/mep_2374-8265.11599

**Published:** 2026-05-21

**Authors:** Karen Chen, Maya Y. Xia, Haley K. Evile, Ted Shi, Harry Liang, Akul Naik, Julia Kim, Charissa Chou, Catherine A. Shu, Usha S. Krishnan

**Affiliations:** 1 Medical Student, Columbia University Vagelos College of Physicians and Surgeons; 2 Associate Professor, Department of Medicine, Columbia University Irving Medical Center; 3 Professor, Department of Pediatrics, Columbia University Irving Medical Center

**Keywords:** Clinical/Procedural Skills Training, Psychiatry, Psychiatry - Child & Adolescent, Qualitative Research

## Abstract

**Introduction:**

Asian American, Native Hawaiian, and Pacific Islander (AANHPI) individuals experience significant mental health disparities but remain largely underrepresented in mental health research and medical education. Addressing this disparity requires educational interventions that improve knowledge of the unique social, cultural, and systemic barriers AANHPI patients face in accessing mental health care.

**Methods:**

We developed and implemented a workshop to educate medical students, clinicians, and health-affiliated professionals on providing culturally responsive mental health care to AANHPI populations. Kern's 6-Step Curriculum Development model was used to create a 1-hour workshop involving a presentation, case-based discussions, reflective exercises, interactive components, and pre/postworkshop evaluations of participants’ knowledge, competence, and confidence (5-point scales; 1 = *Strongly disagree*, 5 = *Strongly agree*). Presentations focused on disparities among AANHPI subgroups, barriers to receiving mental health care, and strategies for engaging with AANHPI populations.

**Results:**

Among 54 participants with pre/postworkshop survey responses, 79% were medical learners, 15% clinicians, and 6% other professionals, and mean scores significantly improved in knowledge of AANHPI mental health disparities (2.6 vs 4.2; *P* < .001), ability to discern sociocultural factors affecting care (3.1 vs 4.2; *P* < .001), and confidence in conducting psychiatric evaluations of AANHPI patients (2.4 vs 3.3; *P* < .001).

**Discussion:**

Implementing this educational workshop significantly improved participants’ knowledge, confidence, and preparedness to address mental health disparities and deliver care to AANHPI populations. Expanding this workshop to reach a broader audience of health care professionals and trainees could promote more equitable, culturally informed mental health care for AANHPI patients.

## Educational Objectives

By the end of this activity, learners will be able to:

1. Demonstrate increased knowledge of cultural influences and social determinants affecting mental health perceptions within AANHPI communities.
2. Express greater confidence in identifying and addressing mental health needs across diverse AANHPI subgroups.
3. Explain culturally responsive strategies for engaging AANHPI patients in mental health care.

## Introduction

Asian American, Native Hawaiian, and Pacific Islander (AANHPI) people represent one of the most diverse and fastest-growing populations in the US.^1^ By 2060, over 36.8 million individuals in the US will identify as AANHPI, spanning more than 50 different subgroups.^2^ Within this diverse population, various health care disparities, such as access to insurance and language barriers, form obstacles to patient care.^3^

One of the most prevalent disparities in AANHPI communities lies in mental health awareness and access to treatment. Up to 23.5% of Asian Americans experience a psychiatric disorder in their lifetime. However, Asian Americans are up to 5 times less likely to seek mental health services compared to their White peers, resulting in poorer mental health outcomes.^4,5^ Native Hawaiians and Pacific Islanders also face significant mental health challenges, with 36.1% reporting symptoms of depression—the highest prevalence among all US racial and ethnic groups.^6^ Since the COVID-19 pandemic, there is even more urgency to address these disparities; mental health outcomes in AANHPI individuals have worsened as a result of COVID-19–related discrimination and hate crimes, with nearly 1 in 3 AANHPI individuals reporting psychological distress, higher than the 2021 national average of 12%.^7^

Within the medical literature, several factors have been proposed to contribute to mental health care disparities in AANHPI communities, including social and self-stigma regarding mental health. The “model minority” stereotype portrays Asian Americans as smart and accomplished, which places pressure on youth to meet the expectations of their community, including non-Asian groups.^8^ This stereotype drives the misconception that Asian Americans are less likely to experience mental health problems, which leads to lower perceived mental health needs.^9^ Within AANHPI communities, mental health can also be regarded as a taboo topic, and certain cultural values, including resilience and self-sufficiency, discourage help-seeking, which may result in underreporting of mental distress and illness.^10^ Furthermore, in some Pacific Islander communities, mental illness is not viewed as seriously as physical illness, which perpetuates stigma.^6^

Additional barriers to mental health care for AANHPI populations include a lack of cultural humility in care, language barriers, lack of insurance coverage, and economic instability. It is also essential to note that AANHPI individuals are often aggregated into a single category, which obscures disparities within certain subgroups.^11^ For example, 6.8% of AANHPI individuals are uninsured, but this frequency of individuals lacking insurance can range from 2.3% of Japanese Americans up to 12.3% of Native Hawaiians and Pacific Islanders.^12^

Improving health care professionals’ understanding of how AANHPI communities perceive mental health has been documented for decades as a potential solution to mental health disparities; however, little progress has been made to incorporate this into medical education.^13^ Many providers are thus unfamiliar with effective strategies for navigating culturally informed conversations or tailoring treatment plans to align with AANHPI patients’ needs. A review of the literature underscores the urgent need for educational interventions that address mental health disparities within AANHPI populations. While much of the research in this area has focused on broader racial and ethnic groups, attention to AANHPI communities, including in standard medical school curricula, remains limited.

Historically, the destigmatization of mental health in AANHPI populations has been underrepresented in medical education; however, recent publications have begun to bridge this gap. Reports such as Beyond the Surface (from The Asian American Foundation) and Piecing the Puzzle of AANHPI Mental Health (from AAPI Data and the UCLA Center for Health Policy Research) provide valuable insights into the cultural, structural, and intergenerational factors that shape mental health experiences in these communities.^14,15^ These works emphasize how stigma, discrimination, and a lack of culturally responsive care contribute to persistent disparities. Furthermore, while *MedEdPORTAL* includes curricula focused on mental health disparities in African American and pediatric populations,^16,17^ no comparable resources currently exist for the AANHPI community. Furthermore, research shows that culturally tailored education improves provider communication, patient trust, and care outcomes. Cultural humility and reflective practice—outlined in reports by Tervalon and Murray-García and by Kumagai and Lypson—enhance responsiveness to diverse populations.^18,19^ AAPI Data further highlights that increased provider awareness leads to better engagement and equity in care for AANHPI patients.^20,21^

This workshop was developed in direct response to persistent gaps in medical education surrounding mental health care for AANHPI populations. Despite growing awareness of racial and ethnic disparities in psychiatric outcomes, medical learners often receive little to no formal instruction on the unique cultural, historical, and structural factors that shape mental health experiences within AANHPI communities. Specifically, training programs rarely provide disaggregated data or frameworks that reflect the diversity of AANHPI subgroups, leading to generalized assumptions and missed opportunities for culturally responsive care. In addition, learners are frequently unprepared to recognize how stigma—rooted in cultural norms, intergenerational dynamics, and systemic barriers—impacts help-seeking behaviors and patient engagement in these communities. Another key deficit was the lack of practical, culturally informed communication strategies in existing curricula, facing uncertainty in navigating sensitive conversations about psychiatric symptoms, family involvement, and treatment preferences with AANHPI patients.

To address these deficits, the workshop was intentionally designed to be accessible to learners across all levels of medical education, with particular relevance for those in their preclinical years. The materials include case-based discussions, reflective exercises, and interactive components that encourage learners to critically examine their assumptions and build confidence in applying culturally grounded approaches. By centering AANHPI voices and emphasizing the intersection of culture, stigma, and clinical practice, the workshop equips future physicians with the tools needed to provide equitable and empathetic mental health care.

## Methods

### Educational Approach

The Kern Six-Step Curriculum Development model was employed to ensure a rigorous and systematic approach to curriculum design.^22^ In the first step, we defined the problem by reviewing academic sources, revealing the underrepresentation of AANHPI mental health in both the literature and medical education. The second step involved a targeted needs assessment to identify specific deficits in the training of medical learners and health care professionals, particularly in addressing cultural and systemic barriers to mental health care. The third and fourth steps focused on establishing clear educational objectives and creating an interactive educational workshop, including a didactic PowerPoint presentation (Appendix A) with clinical case studies and interactive questions. These activities were designed to actively engage participants and encourage the application of newly acquired knowledge. The fifth and sixth steps involved implementation, evaluation, and feedback, with the workshop piloted across diverse student body audiences and assessed through pre- and postworkshop surveys to measure its impact on participants’ knowledge, attitudes, and skills (Appendix B).

### Facilitators and Target Audience

The 1-hour workshop was delivered 4 times in Fall 2024: at a virtual medical education conference attended primarily by medical students and physicians, at an in-person AANHPI affinity group conference for medical learners, and at 2 in-person sessions open to all learners at the medical school. The Columbia University Institutional Review Board determined the workshop to be not human subjects research (No. AAAV4220) under Federal Policy for the Protection of Human Subjects common rule 45 CFR part 46.

Each session was facilitated by a team of 4 medical students interested in improving care for AANHPI patients. Before implementation, facilitators reviewed the slide deck, evaluation forms, and facilitator guide, which required approximately 2 hours of preparation. The facilitator guide was used to standardize delivery across sessions by outlining the sequence of the workshop, pairing each slide with key teaching points, and providing suggested timing to support consistency, pacing, and engagement. Although the same facilitator team delivered all sessions, each facilitator was prepared to lead any portion of the workshop to maintain flexibility in the event of personnel changes.

The workshop was designed for a broad audience of physicians-in-training and is most appropriate for elective or diversity, equity, and inclusion programming. It does not require extensive prior knowledge. Although intended primarily to supplement preclinical medical education, it is also suitable for third- and fourth-year learners, particularly those who have completed preclinical or clinical psychiatry training and can revisit familiar concepts through the lens of disparities in psychiatric care for AANHPI patients. A brief introduction to relevant psychiatric disorders is included to ensure accessibility for learners with varying levels of prior exposure.

### Implementation

The workshop was designed to be completed in approximately 60 minutes, including participant orientation and evaluation. Required materials included standard audiovisual equipment for slide projection, the workshop slide deck, printed or electronic evaluation forms, and the facilitator guide. Materials are provided in Appendix A (PowerPoint presentation), Appendix B (pre/postworkshop evaluation forms), and Appendix C (facilitator guide).

Before each session, facilitators reviewed the workshop objectives, slide content, key discussion points, and pacing recommendations, using the slide deck and facilitator guide. At the start of the session, learners were introduced to the purpose of the workshop and asked to complete the preworkshop evaluation form (Appendix B), which required approximately 5–10 minutes. This form assessed baseline knowledge of mental health disparities among AANHPI subgroups, self-rated confidence in discussing these topics, perceptions of curricular emphasis at their medical school, and demographic characteristics.

Facilitators then delivered the main workshop content over approximately 40–45 minutes using the 48-slide presentation (Appendix A), with guidance from the facilitator guide (Appendix C) to ensure consistent coverage of core concepts and key teaching points. Learners were asked to engage with the material and participate in discussion as prompted. At the end of the session, learners completed the postworkshop evaluation form (Appendix B), which took approximately 5–10 minutes. This form retained the core knowledge, confidence, and curricular assessment items from the preworkshop evaluation to allow pre/postworkshop comparison, while replacing demographic questions with items assessing workshop quality and perceived educational value.

The workshop can be implemented in a single session with one or more facilitators, a computer, projection capability, and either paper or electronic access to the evaluation forms. The appendices provide the materials needed for replication at other institutions.

### Evaluation and Analysis

Paired pre- and postworkshop questionnaires (Appendix B) were used to assess immediate changes in learner knowledge, self-reported confidence, and perceptions of curricular emphasis related to mental health disparities among AANHPI subgroups. The instruments were developed by the workshop authors on the basis of the session's educational objectives. The preworkshop questionnaire included 11 items assessing baseline knowledge, confidence, curricular perceptions, and demographics. The postworkshop questionnaire also included 11 items, retaining the core knowledge, confidence, and curricular assessment items for pre/postworkshop comparison while replacing demographic items with questions about workshop quality and participant feedback.

Analysis focused on 3 domains aligned with the workshop objectives: knowledge of mental health disparities among AANHPI populations, ability to identify how social and cultural factors influence care for AANHPI patients, and confidence in conducting a psychiatric evaluation of an AANHPI patient. Confidence-related items, including perceptions of whether participants’ medical schools adequately addressed these topics, were measured on 5-point Likert scales (1 = *Strongly disagree*, 5 = *Strongly agree*) and analyzed using the Wilcoxon signed rank test. Knowledge-based responses were analyzed using paired *t* tests. Statistical significance was defined as a 2-sided *P* value less than .05.

## Results

Across the 4 workshop offerings, there were 11, 20, 14, and 25 participants, respectively, for a total of 70 attendees. Of these, 54 participants submitted paired preworkshop and postworkshop survey responses, corresponding to a paired survey response rate of 77%. Most participants were from a single medical school, where the workshop was primarily implemented. Approximately 15% of participants (*n* = 8) came from 5 additional medical schools across the US. Among the 54 participants, 53 (98%) reported their occupational status as medical learners (*n* = 42, 79%), clinicians (*n* = 8, 15%), or individuals with other occupations (*n* = 3, 6%). The cohort was ethnically diverse, with 50 participants (93%) reporting ethnic data, identifying as Asian (*n* = 25, 50%), Hispanic/Latinx (*n* = 11, 22%), White (*n* = 10, 20%), African American/Black (*n* = 2, 4%), or another ethnicity (*n* = 2, 4%).

There were significant improvements from the preworkshop survey to postworkshop survey in participants’ knowledge regarding mental health disparities in AANHPI populations (mean score 2.6 vs 4.2; *P* < .001), ability to discern how social and cultural factors affect care in AANHPI populations (mean score 3.1 vs 4.2; *P* < .001), and confidence in conducting a psychiatric evaluation of AANHPI patients (mean score 2.4 vs 3.3; *P* < .001) (Table 1). Participants’ views on their medical school training on this topic did not change significantly from pre- to postworkshop (mean score 2.3 vs 2.6; *P* = .30), with participants generally disagreeing that their medical school was doing a good job teaching about this topic (Table 1).

**Table 1.**
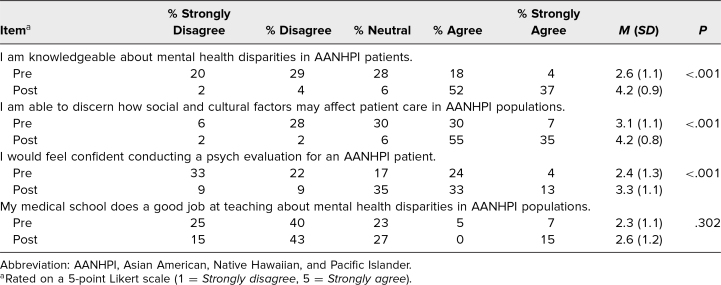
Overall Participant Self-Reported Ratings of Knowledge, Competency, and Confidence Before and After the Addressing Mental Health Disparities in AANHPI Communities Workshop (*N* = 54)

When stratified by ethnicity, participants self-identifying as AANHPI (*n* = 25) and those reporting other ethnicities (non-AANHPI; *n* = 29) showed similar significant improvements in their overall self-reported ratings in response to all of the competency and belief-related questions, except that there was no significant improvement in confidence in conducting a psychiatric assessment of AANHPI patients. Furthermore, non-AANHPI participants generally reported lower confidence in all 3 competency areas (Table 2).

**Table 2.**
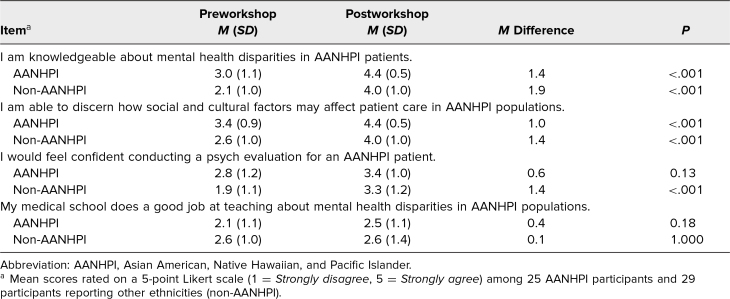
Participant Self-Reported Ratings of Knowledge, Competency, and Confidence by Ethnicity, Before and After the Addressing Mental Health Disparities in AANHPI Communities Workshop (*N* = 54)

In addition, in the preworkshop survey, we specifically assessed participants’ knowledge regarding mental health disparities in AANHPI populations with 4 multiple-choice questions. These questions were discussed during the workshop and were asked again in the postworkshop survey. All 4 questions showed improvements in correct responses between the preworkshop and postworkshop survey (Table 3).

**Table 3.**
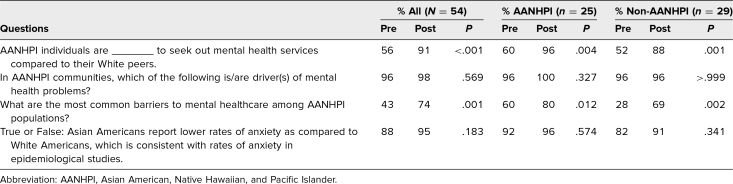
Percentage of Correct Answers in Response to Multiple-Choice Knowledge Questions on Pre- and Postworkshop Surveys

Qualitative feedback from the postworkshop survey assessed the workshop's effectiveness in meeting the educational objectives. Thirty-one participants (57%) completed this survey item, with 26 affirming that the educational objectives were met. Overall, participants described the workshop as engaging, informative, and effective in increasing awareness of mental health disparities and social determinants of health affecting AANHPI populations.

Qualitative feedback also included participants’ comments with suggestions for improvements, focused on (1) highlighting treatments/initiatives for mental health disparities or (2) the structure and delivery of the workshop. Qualitative feedback was largely favorable and suggested opportunities to further strengthen the workshop's practical relevance and interactivity. Two themes were most salient. First, participants expressed interest in more actionable content, including examples of current initiatives to address AANHPI mental health disparities, culturally grounded healing practices, and concrete resources for continued learning. Second, several comments suggested making the session more interactive and case based, such as incorporating small-group discussion, live polling, and more individualized patient stories to deepen engagement and illustrate how the material applies in practice.

## Discussion

We successfully developed and delivered a workshop for health care professionals and trainees that improved knowledge about mental health disparities in AANHPI patients and increased their ability to discern how social and cultural factors may affect patient care in AANHPI populations. This workshop addresses an important need in current mental health training by educating participants on the influence of cultural beliefs, social determinants of health, and systemic barriers on mental health outcomes in AANHPI communities, and by providing effective strategies for navigating culturally informed conversations with AANHPI patients. Our emphasis on specific mental health concerns within different AANHPI subgroups also highlights the importance of understanding the cultural complexities and diversity within AANHPI communities.^16^

Overall, the workshop was well received by participants and effectively met the educational objectives. There was a statistically significant increase in participants’ knowledge about mental health disparities in AANHPI patients, ability to discern how social and cultural factors may affect patient care in AANHPI populations, and confidence in conducting a psychiatric evaluation of an AANHPI patient. Participants’ feedback highlighted case studies and interactive polling as effective teaching measures.

Based on participants’ feedback, improvements were made after every session. These improvements included changing the discussion questions individual audience members volunteered to answer to anonymous polling, which increased engagement and participation, and clarifying the presentation of statistics on the slides. Further suggestions for improvement include personalized stories from individuals of more ethnic groups, and a deeper discussion of how the Western-minded approach to medicine can be at odds with AANHPI cultures.

While participants had improved confidence in conducting a psychiatric evaluation of an AANHPI patient postworkshop, this improvement was smaller than the increase in confidence in improved knowledge about disparities and discerning AANHPI social and cultural factors. This finding suggests a gap in the workshop, and future iterations should include more explicit language and questions for participants to use in their clinical encounters.

There are several limitations to our workshop. First, we did not assess participants’ prior experience in psychiatric settings, which may have affected the workshop's impact. Most attendees were preclinical medical learners with limited exposure to psychiatric patients, contributing to low confidence in conducting psychiatric evaluations with AANHPI individuals, both before and after the workshop. Additionally, while we measured immediate changes in knowledge and confidence, we did not assess long-term retention or behavioral changes in clinical practice. Future evaluations should include longitudinal follow-up and implementation across diverse educational settings to better understand clinical impact.

The 1-hour format also limited our ability to explore a broader range of ethnic experiences or delve deeply into cultural tensions between Western medical models and AANHPI perspectives. Expanding the workshop's duration and scope would allow for richer engagement with these themes.

Survey design limitations further impacted interpretation. Question 3 was overly general and was revised for specificity, while Question 4 was revised for clarity. High pretest scores on several items suggest a ceiling effect, limiting measurable change. As an exploratory tool, the survey lacked formal validation; future versions will incorporate validated instruments to improve reliability. The confidence item combined 2 distinct concepts and will be split into separate items in future iterations. Finally, overrepresentation of AANHPI participants may have skewed responses toward greater interest and familiarity with the topic, which we acknowledge as a limitation in generalizability.

To build on this initial workshop, we plan to develop a series of follow-up educational interventions that address additional dimensions of mental health care in AANHPI communities. Future workshops will explore topics such as stigma reduction strategies, intergenerational dynamics affecting help-seeking behavior, and culturally adapted clinical approaches tailored to specific AANHPI subgroups. Furthermore, we also plan to include cohort comparisons to assess differences between workshop participants and clinical learners exposed only to the standard curriculum. By expanding the curriculum in this way, we aim to provide learners with a more comprehensive, clinically applicable framework for delivering equitable, culturally responsive mental health care.

## Appendices


AANHPI Mental Health Workshop.pptxPre- and Postworkshop Survey.docxFacilitator Guide.docx
*All appendices are peer reviewed as integral parts of the Original Publication.*
