# Supplementary material for: Enhancing Cultural Humility: Addressing Mental Health Disparities in AANHPI Communities
Source: MedEdPORTAL. 2026 May 21;22:11599. doi: 10.15766/mep_2374-8265.11599 (PMC13192378; doi:10.15766/mep_2374-8265.11599)
Supplement: Supplementary file 1 — AANHPI Mental Health Workshop.pptxPre- and Postworkshop Survey.docxFacilitator Guide.docx [file mep_2374-8265.11599-s001.zip › A. AANHPI Mental Health Workshop.pptx]

## Slide 1
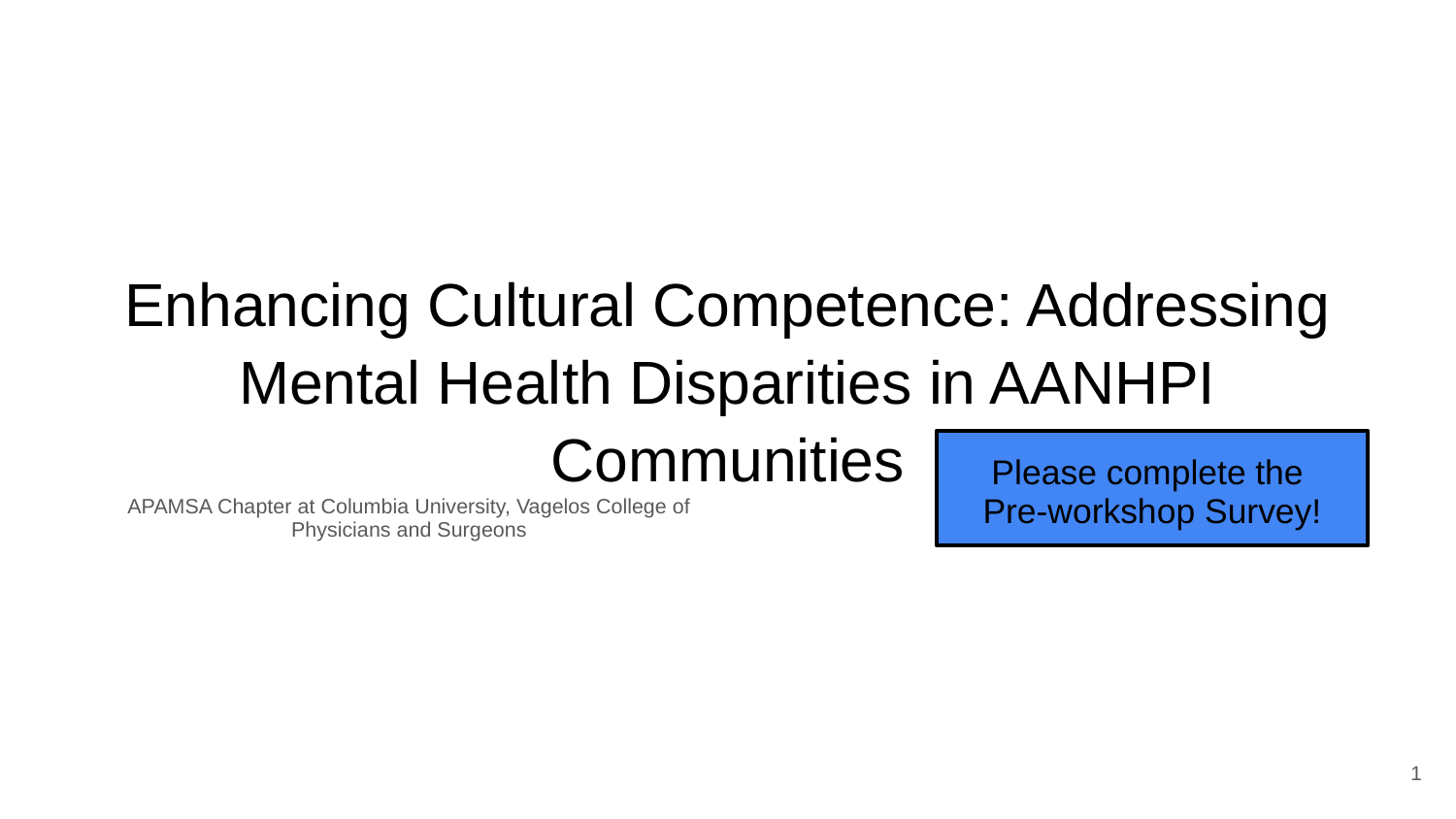

# Enhancing Cultural Competence: Addressing Mental Health Disparities in AANHPI Communities
Please complete the
Pre-workshop Survey!
APAMSA Chapter at Columbia University, Vagelos College of Physicians and Surgeons
‹#›

## Slide 2
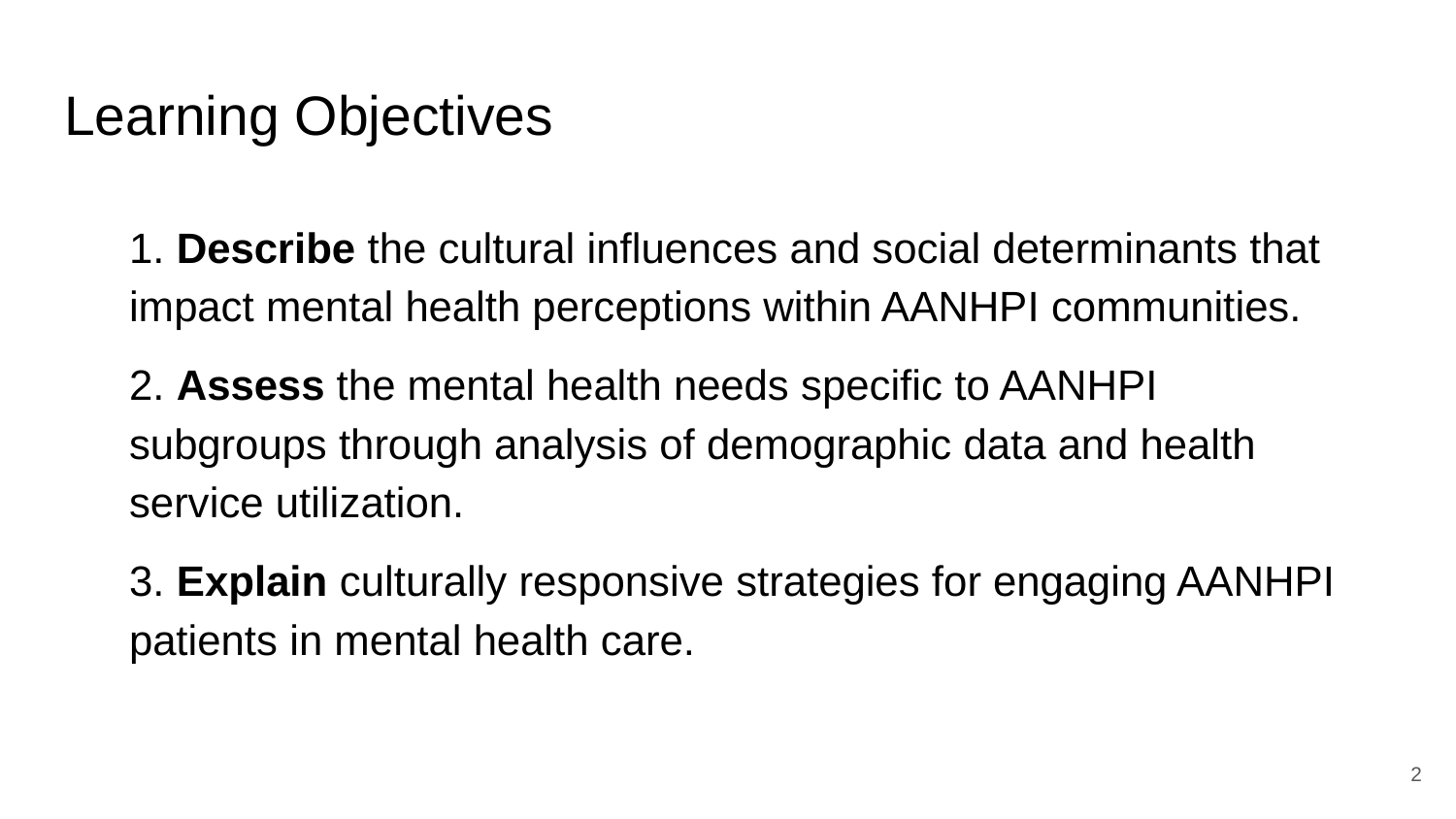

# Learning Objectives
1. Describe the cultural influences and social determinants that impact mental health perceptions within AANHPI communities.
2. Assess the mental health needs specific to AANHPI subgroups through analysis of demographic data and health service utilization.
3. Explain culturally responsive strategies for engaging AANHPI patients in mental health care.
‹#›

## Slide 3
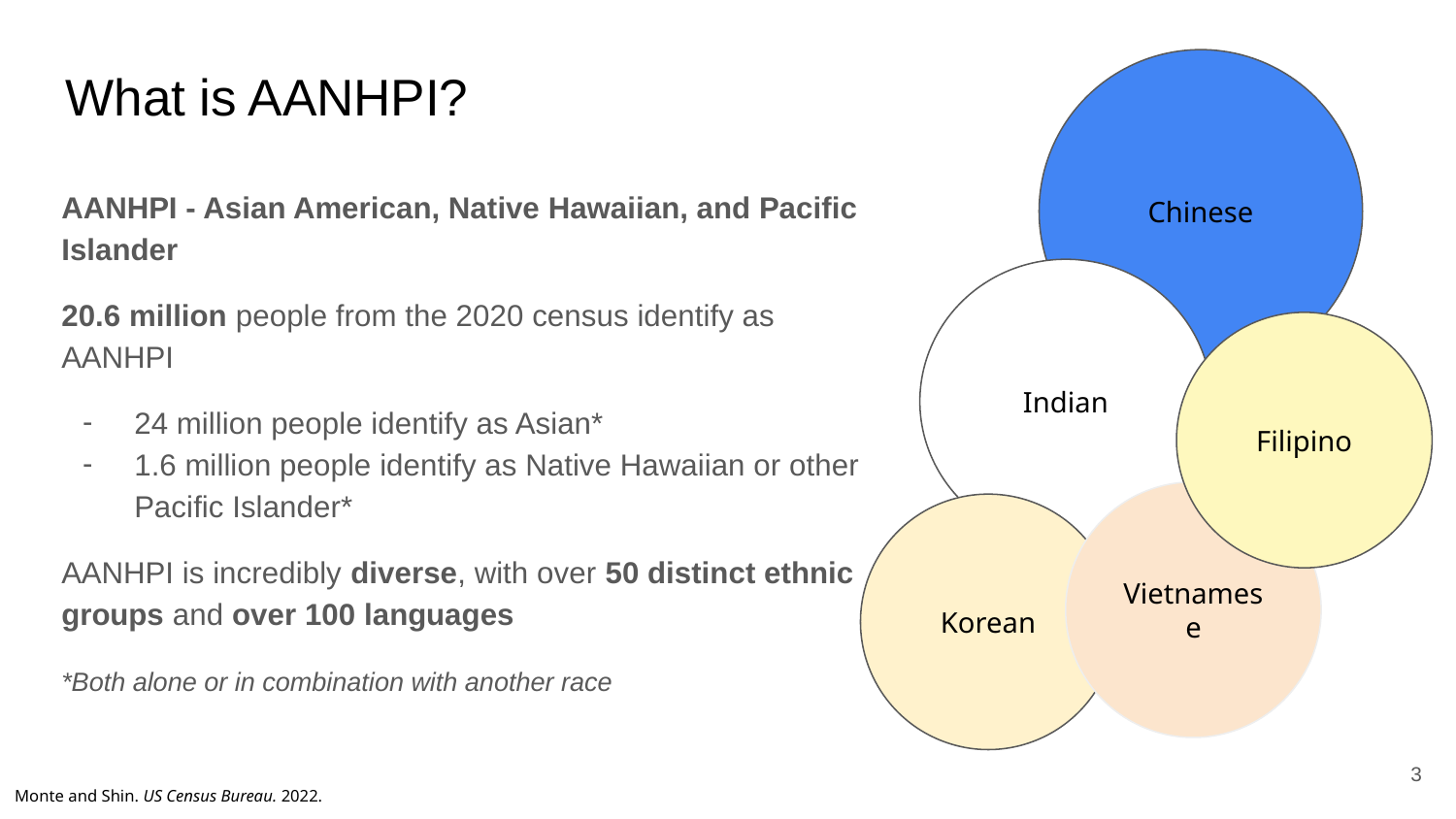

# What is AANHPI?
Chinese
AANHPI - Asian American, Native Hawaiian, and Pacific Islander
20.6 million people from the 2020 census identify as AANHPI
24 million people identify as Asian*
1.6 million people identify as Native Hawaiian or other Pacific Islander*
AANHPI is incredibly diverse, with over 50 distinct ethnic groups and over 100 languages
*Both alone or in combination with another race
Indian
Filipino
Vietnamese
Korean
‹#›
Monte and Shin. US Census Bureau. 2022.

## Slide 4
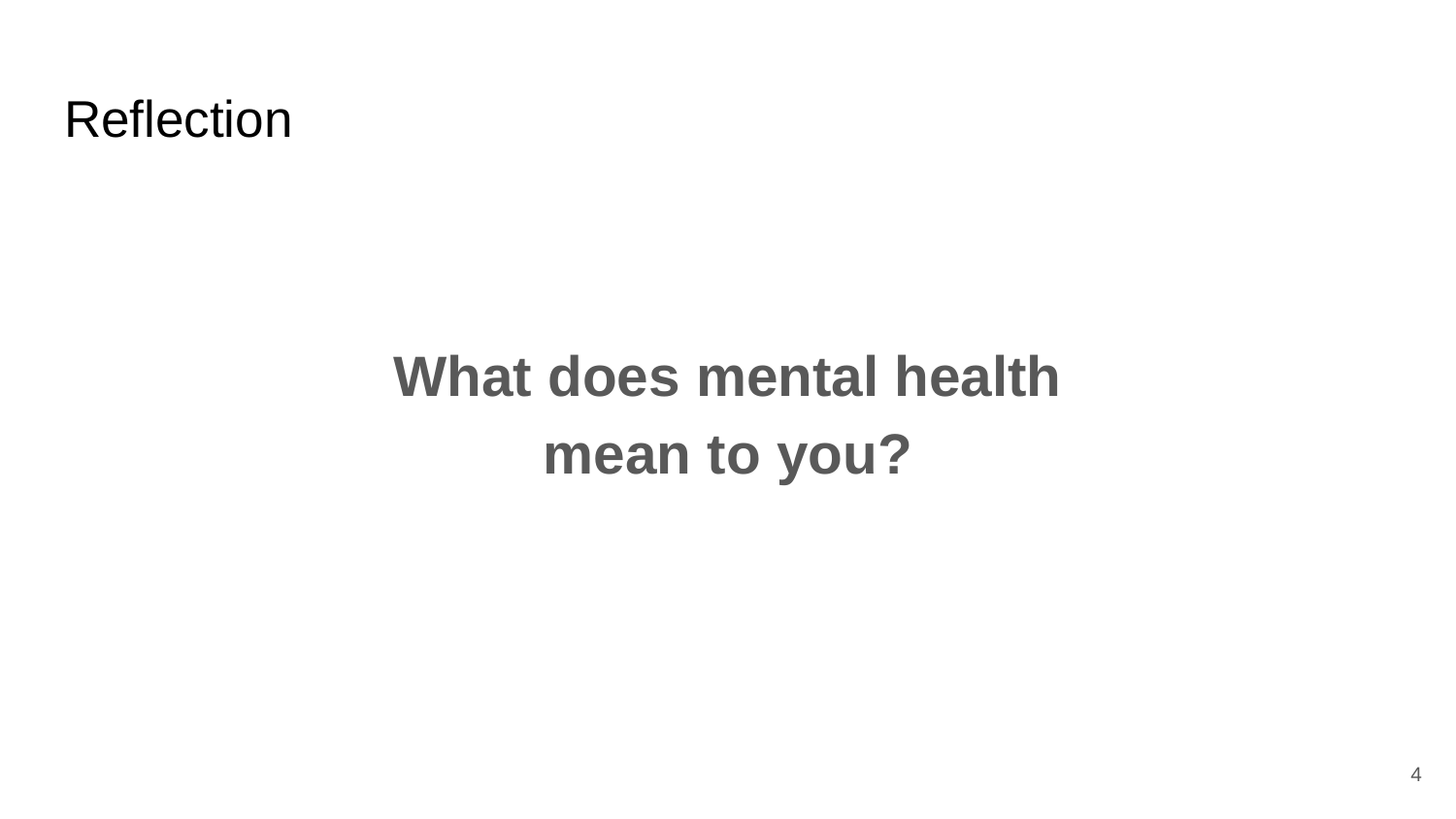

# Reflection
What does mental health mean to you?
‹#›

## Slide 5
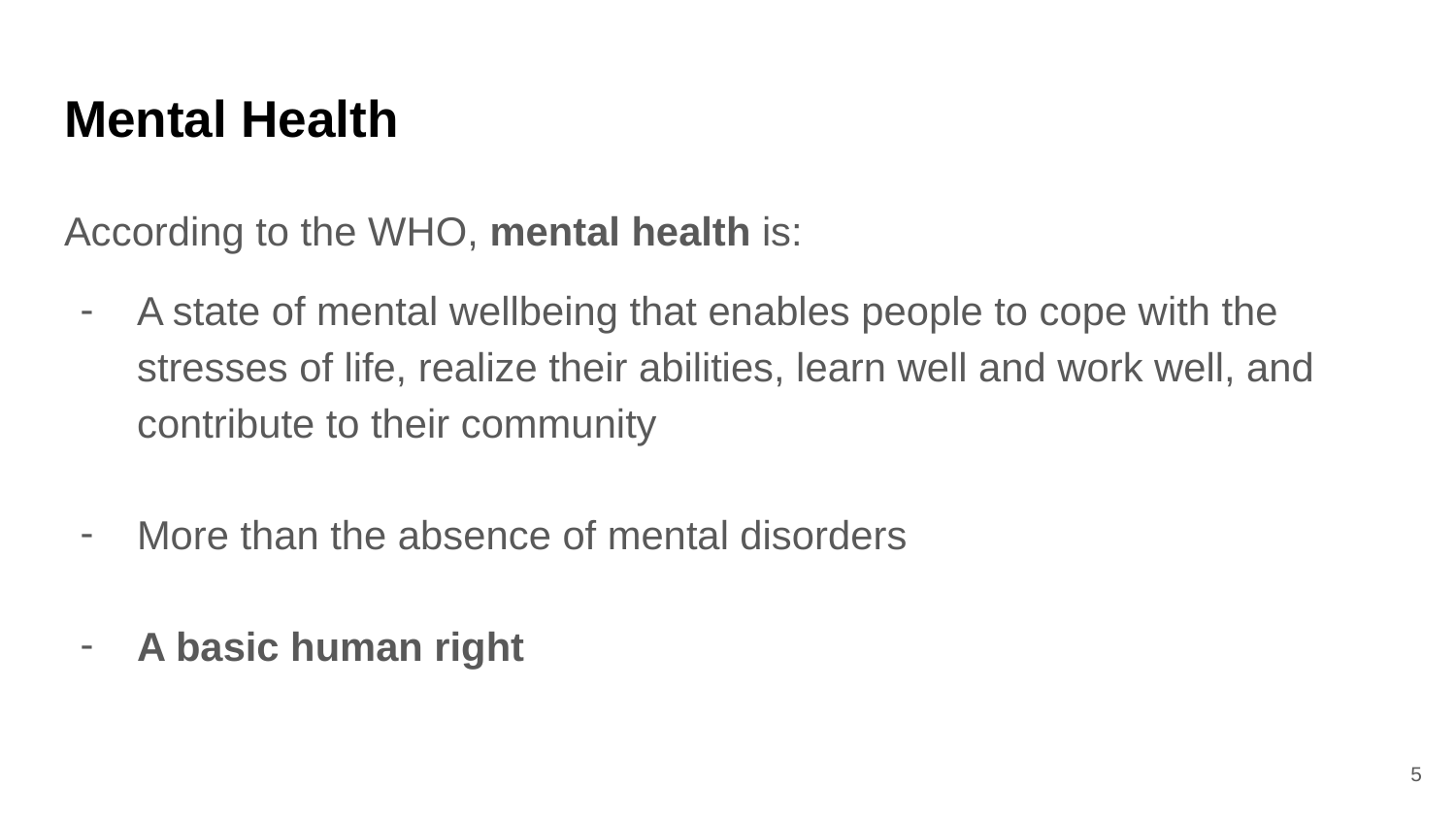

# Mental Health
According to the WHO, mental health is:
A state of mental wellbeing that enables people to cope with the stresses of life, realize their abilities, learn well and work well, and contribute to their community
More than the absence of mental disorders
A basic human right
‹#›

## Slide 6
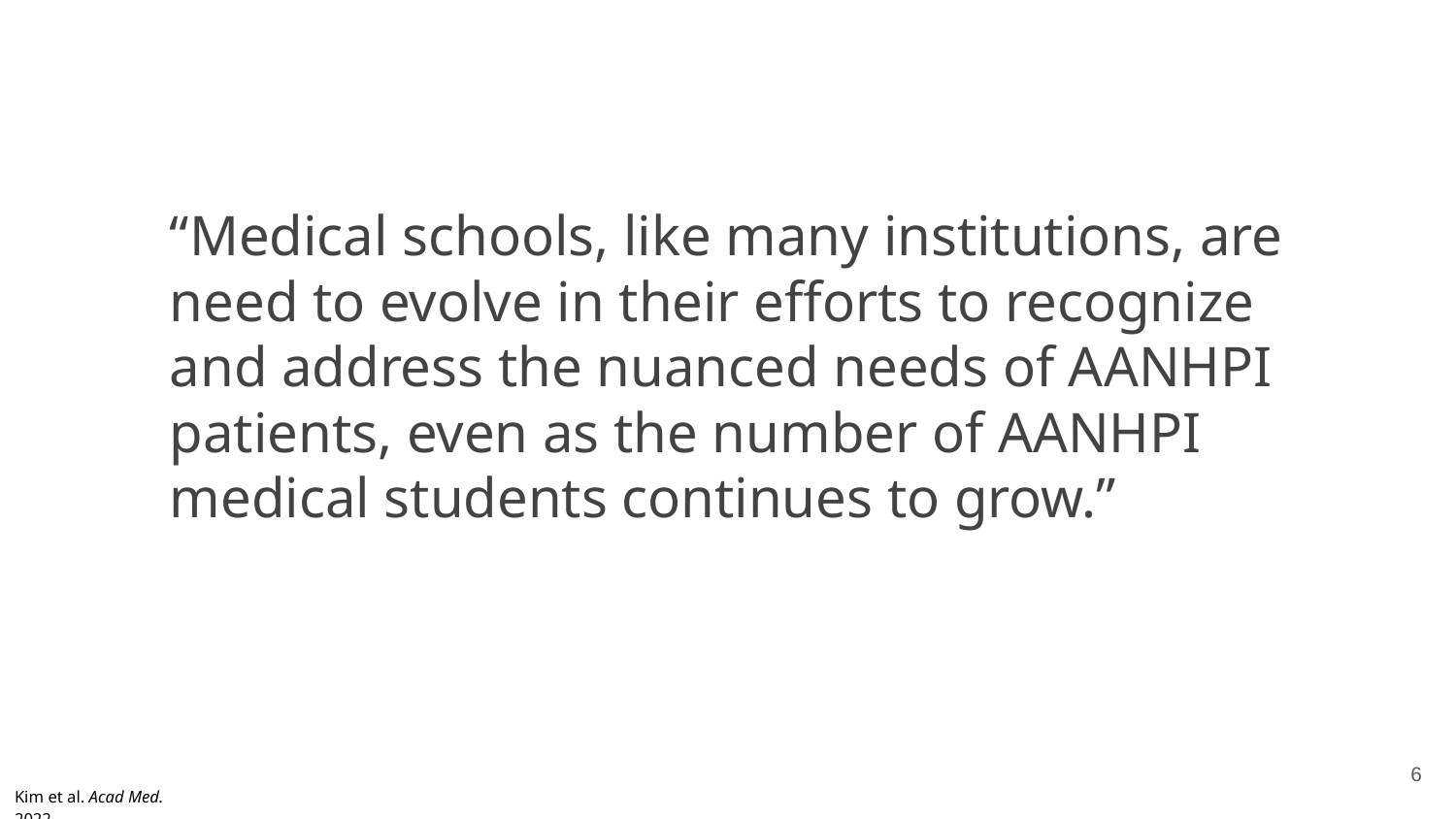

# “Medical schools, like many institutions, are need to evolve in their efforts to recognize and address the nuanced needs of AANHPI patients, even as the number of AANHPI medical students continues to grow.”
‹#›
Kim et al. Acad Med. 2022.

## Slide 7
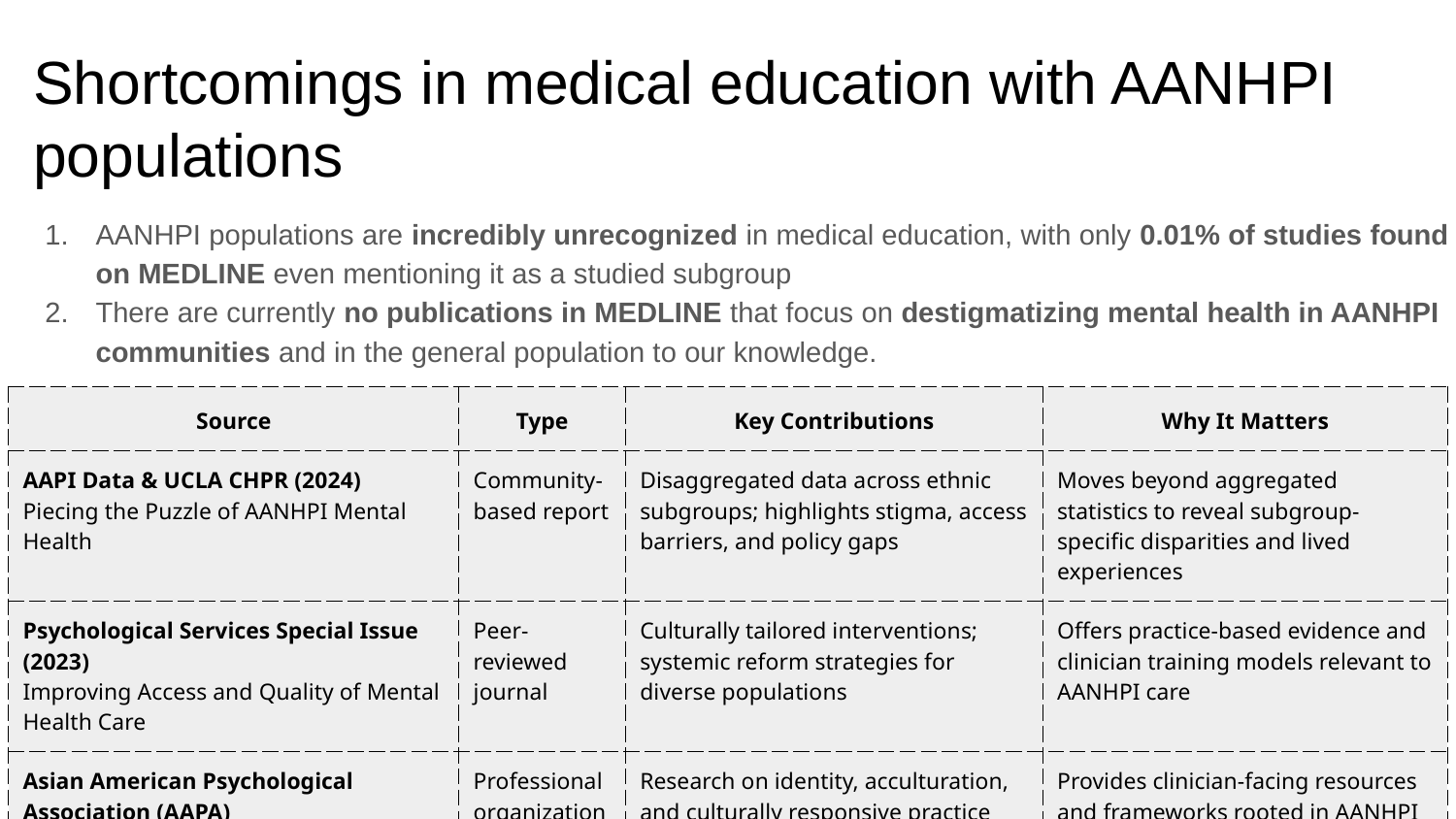

# Shortcomings in medical education with AANHPI populations
AANHPI populations are incredibly unrecognized in medical education, with only 0.01% of studies found on MEDLINE even mentioning it as a studied subgroup
There are currently no publications in MEDLINE that focus on destigmatizing mental health in AANHPI communities and in the general population to our knowledge.
| Source | Type | Key Contributions | Why It Matters |
| --- | --- | --- | --- |
| AAPI Data & UCLA CHPR (2024) Piecing the Puzzle of AANHPI Mental Health | Community-based report | Disaggregated data across ethnic subgroups; highlights stigma, access barriers, and policy gaps | Moves beyond aggregated statistics to reveal subgroup-specific disparities and lived experiences |
| Psychological Services Special Issue (2023) Improving Access and Quality of Mental Health Care | Peer-reviewed journal | Culturally tailored interventions; systemic reform strategies for diverse populations | Offers practice-based evidence and clinician training models relevant to AANHPI care |
| Asian American Psychological Association (AAPA) AAPA Publications | Professional organization | Research on identity, acculturation, and culturally responsive practice | Provides clinician-facing resources and frameworks rooted in AANHPI psychology |
‹#›

## Slide 8
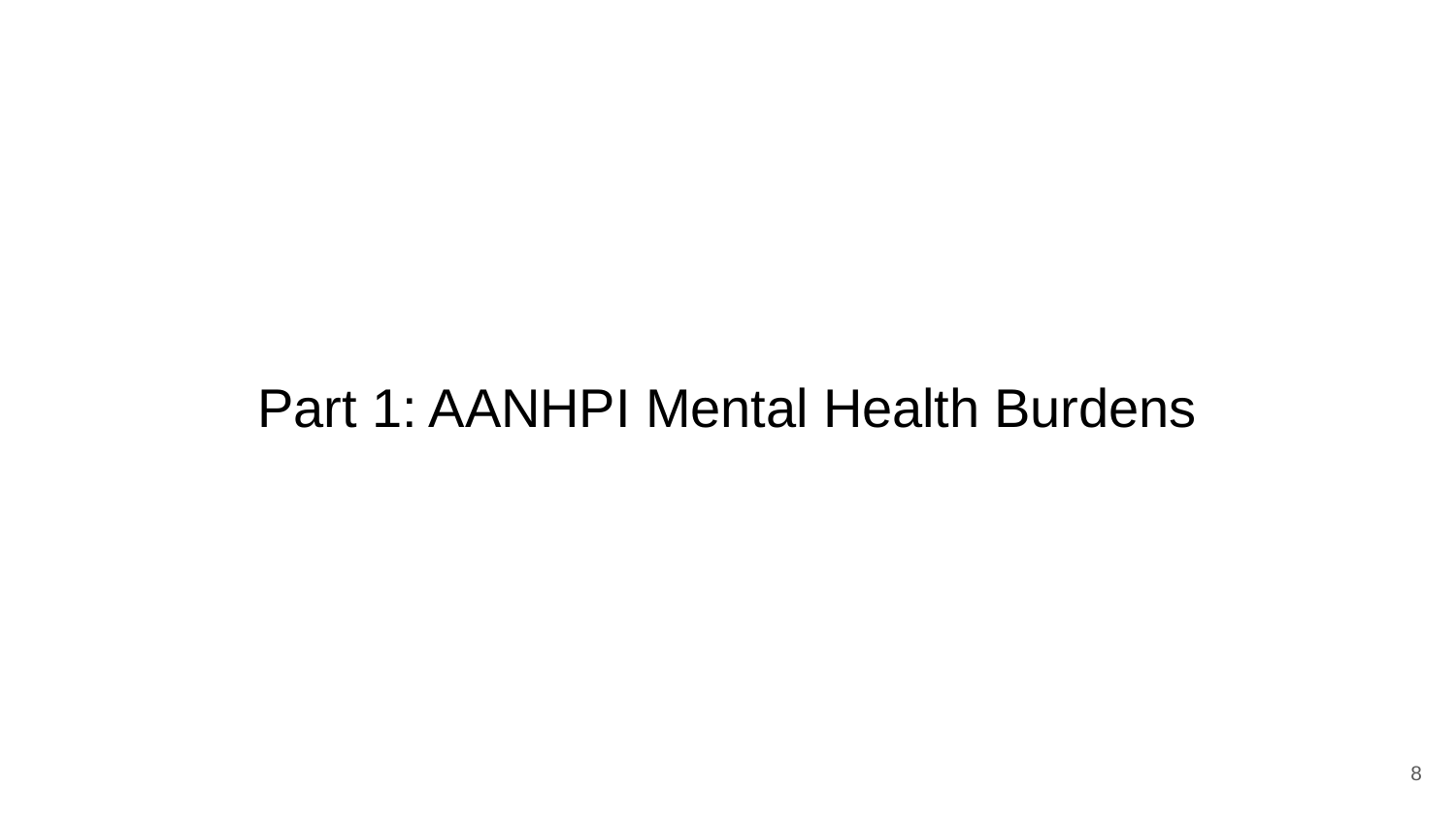

# Part 1: AANHPI Mental Health Burdens
‹#›

## Slide 9
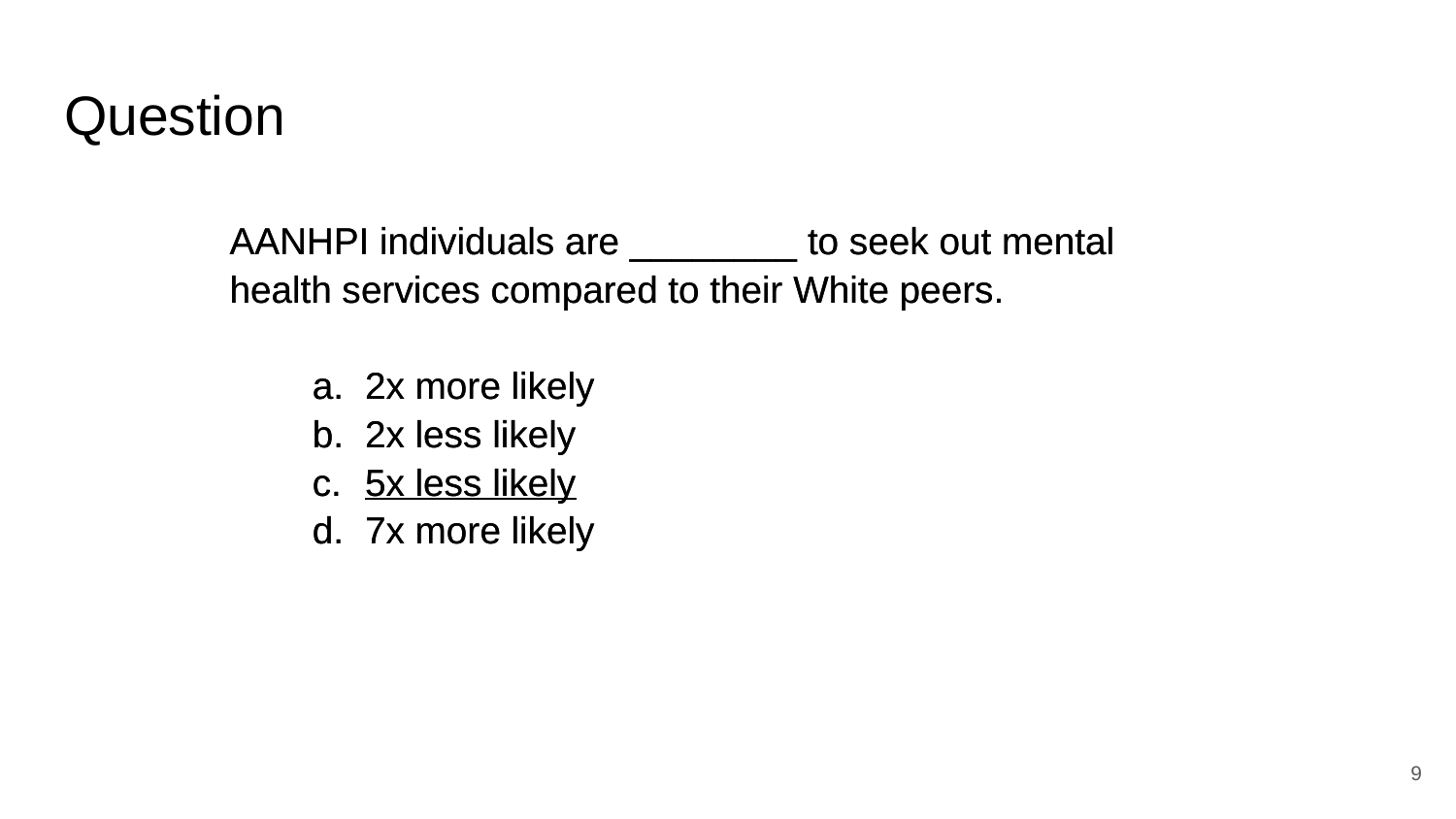

# Question
AANHPI individuals are ________ to seek out mental health services compared to their White peers.
2x more likely
2x less likely
5x less likely
7x more likely
AANHPI individuals are ________ to seek out mental health services compared to their White peers.
2x more likely
2x less likely
5x less likely
7x more likely
‹#›

## Slide 10
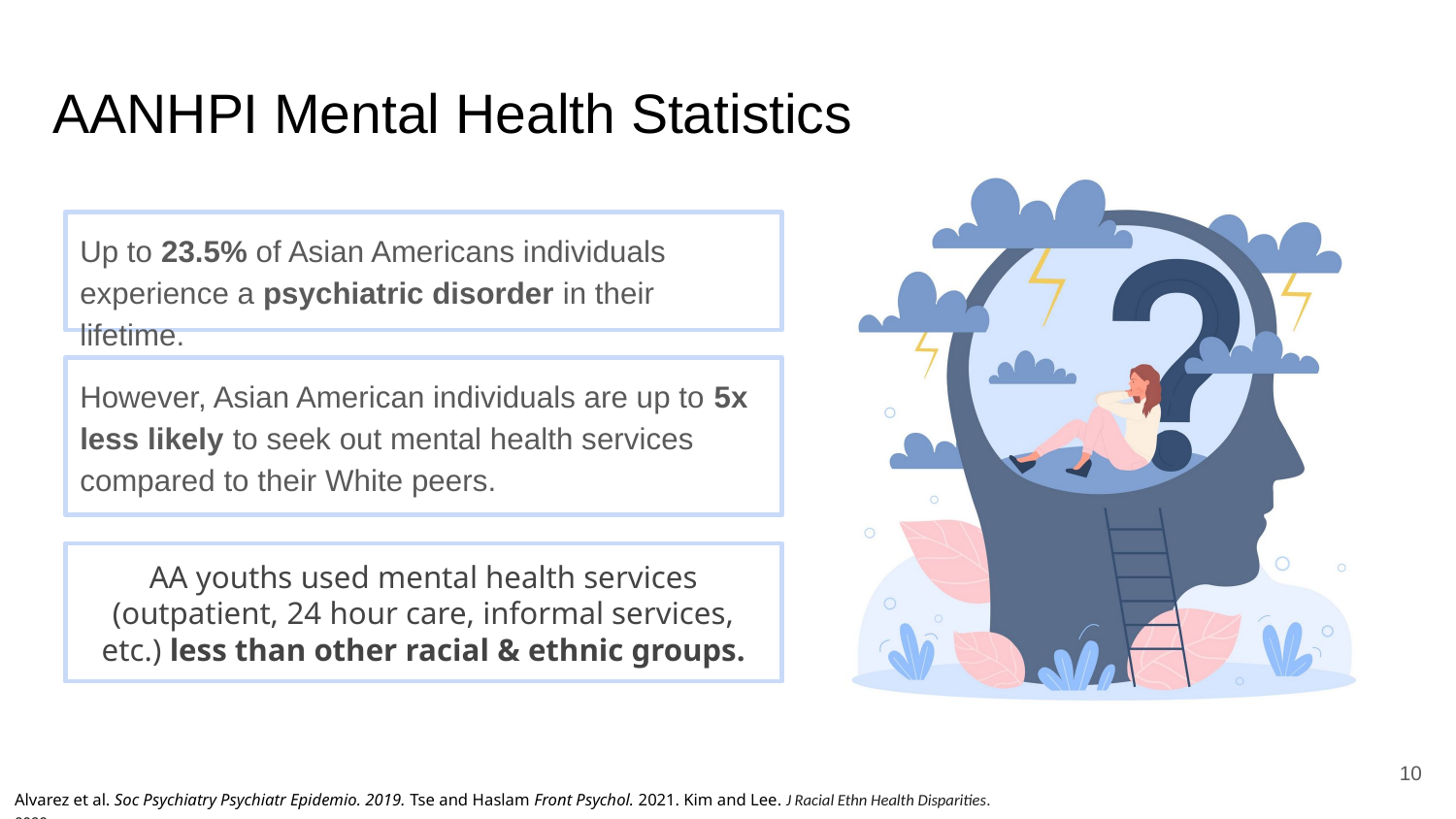

AANHPI Mental Health Statistics
Up to 23.5% of Asian Americans individuals experience a psychiatric disorder in their lifetime.
However, Asian American individuals are up to 5x less likely to seek out mental health services compared to their White peers.
AA youths used mental health services (outpatient, 24 hour care, informal services, etc.) less than other racial & ethnic groups.
‹#›
Alvarez et al. Soc Psychiatry Psychiatr Epidemio. 2019. Tse and Haslam Front Psychol. 2021. Kim and Lee. J Racial Ethn Health Disparities. 2022.

## Slide 11
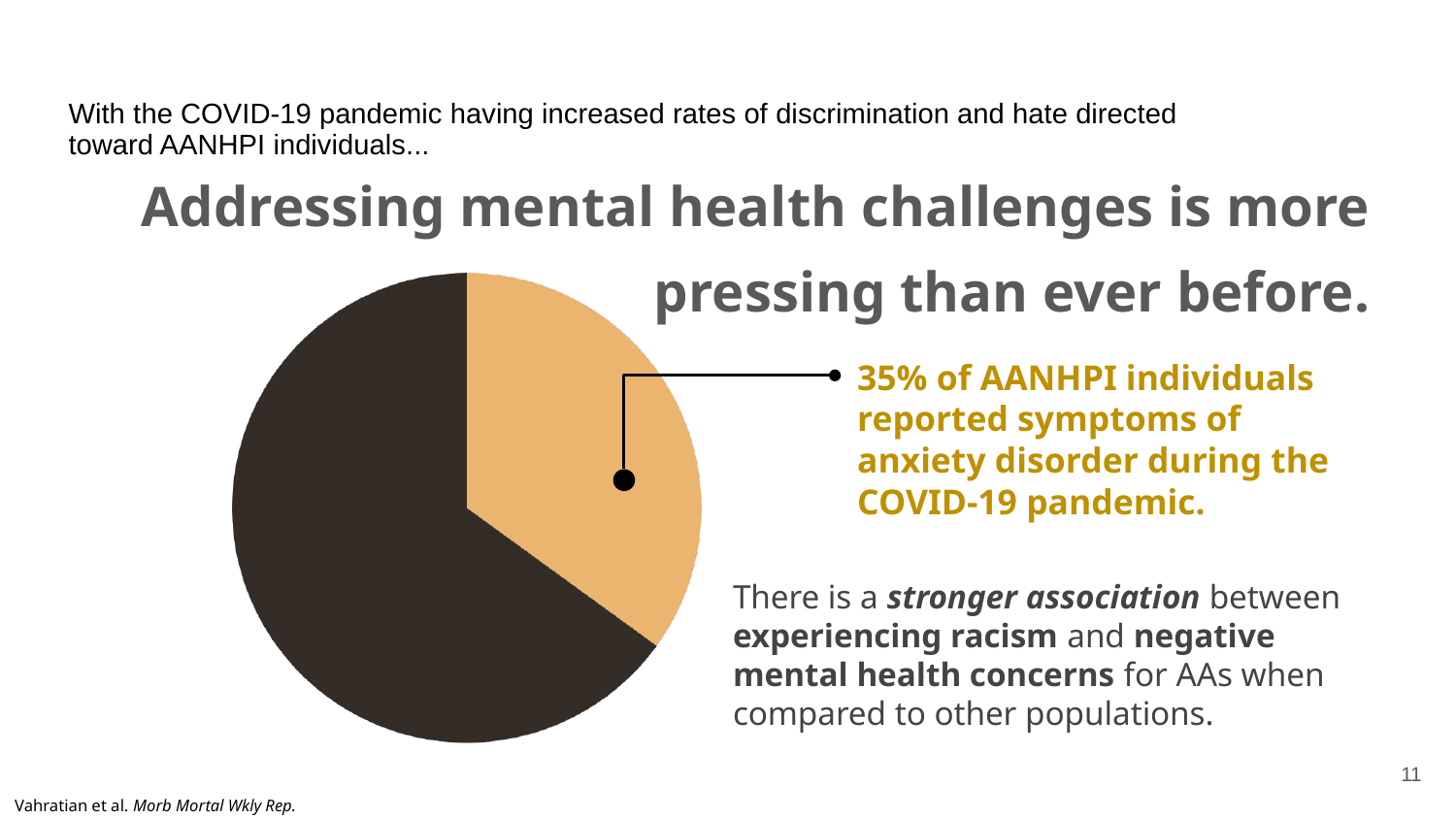

# With the COVID-19 pandemic having increased rates of discrimination and hate directed toward AANHPI individuals...
Addressing mental health challenges is more pressing than ever before.
35% of AANHPI individuals reported symptoms of anxiety disorder during the COVID-19 pandemic.
There is a stronger association between experiencing racism and negative mental health concerns for AAs when compared to other populations.
‹#›
Vahratian et al. Morb Mortal Wkly Rep. 2021.

## Slide 12
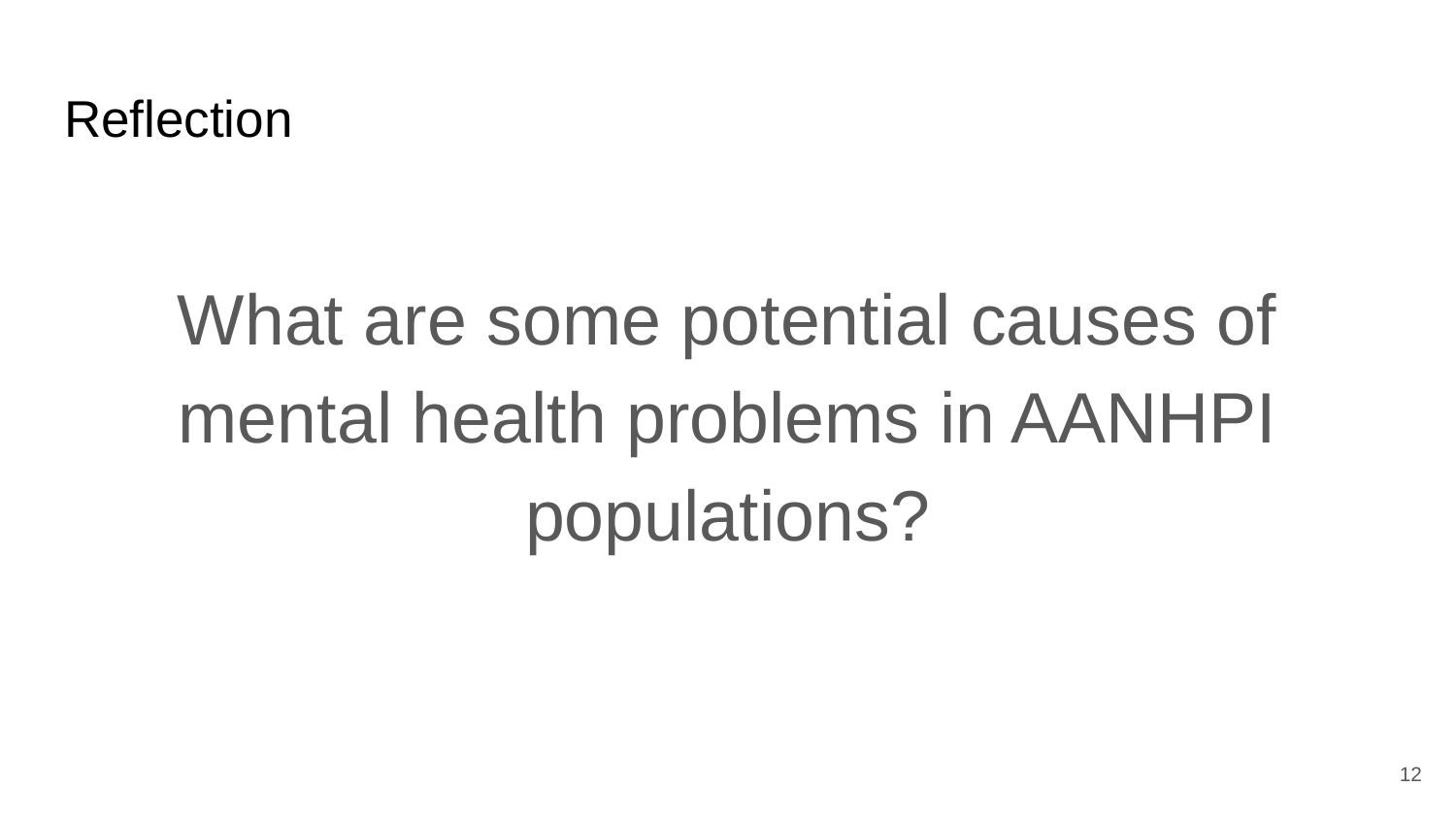

# Reflection
What are some potential causes of mental health problems in AANHPI populations?
‹#›

## Slide 13
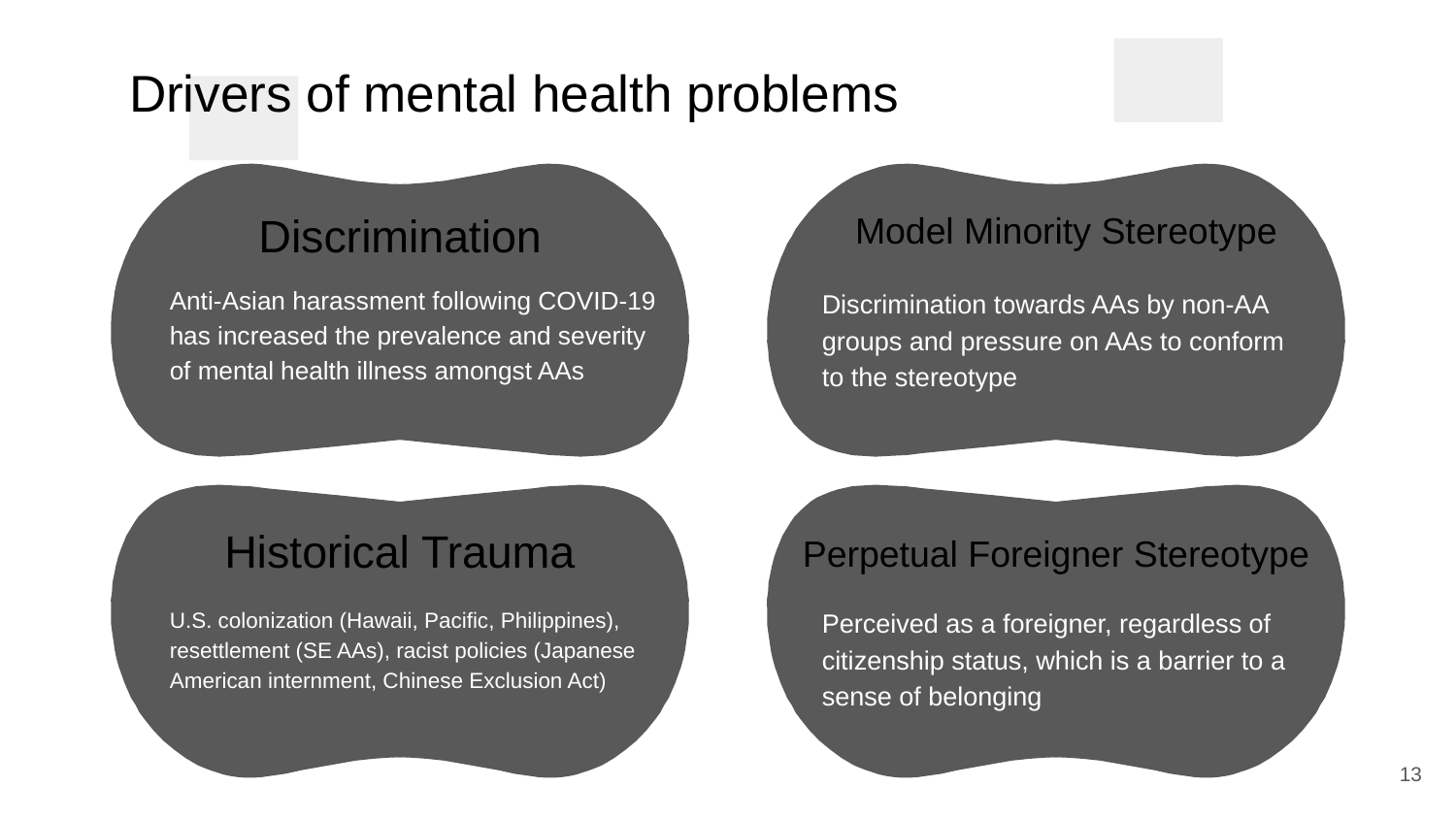

Drivers of mental health problems
Discrimination
Model Minority Stereotype
Anti-Asian harassment following COVID-19 has increased the prevalence and severity of mental health illness amongst AAs
Discrimination towards AAs by non-AA groups and pressure on AAs to conform to the stereotype
Historical Trauma
Perpetual Foreigner Stereotype
U.S. colonization (Hawaii, Pacific, Philippines), resettlement (SE AAs), racist policies (Japanese American internment, Chinese Exclusion Act)
Perceived as a foreigner, regardless of citizenship status, which is a barrier to a sense of belonging
‹#›

## Slide 14
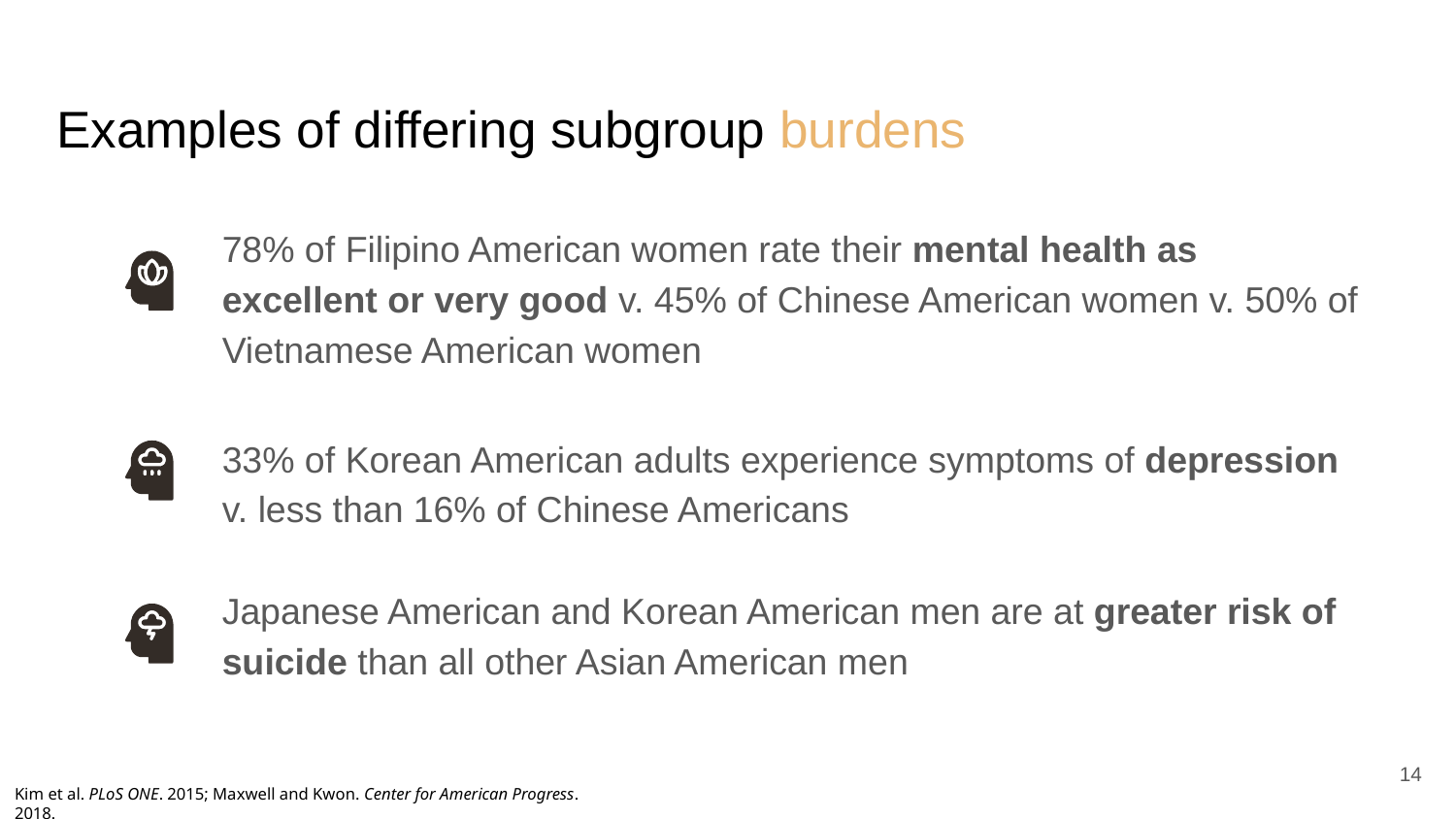

# Examples of differing subgroup burdens
78% of Filipino American women rate their mental health as excellent or very good v. 45% of Chinese American women v. 50% of Vietnamese American women
33% of Korean American adults experience symptoms of depression v. less than 16% of Chinese Americans
Japanese American and Korean American men are at greater risk of suicide than all other Asian American men
‹#›
Kim et al. PLoS ONE. 2015; Maxwell and Kwon. Center for American Progress. 2018.

## Slide 15
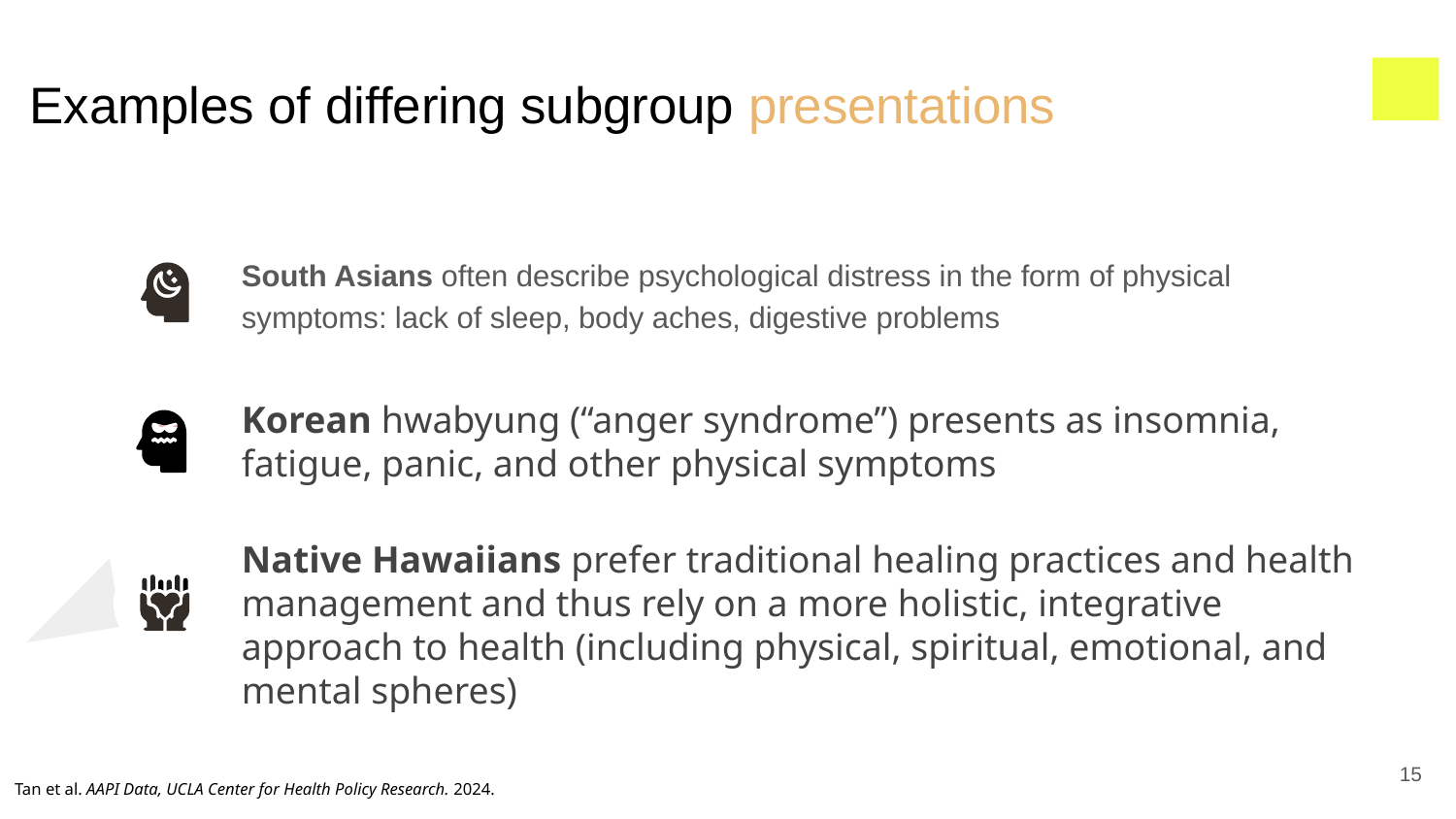

# Examples of differing subgroup presentations
South Asians often describe psychological distress in the form of physical symptoms: lack of sleep, body aches, digestive problems
Korean hwabyung (“anger syndrome”) presents as insomnia, fatigue, panic, and other physical symptoms
Native Hawaiians prefer traditional healing practices and health management and thus rely on a more holistic, integrative approach to health (including physical, spiritual, emotional, and mental spheres)
‹#›
Tan et al. AAPI Data, UCLA Center for Health Policy Research. 2024.

## Slide 16
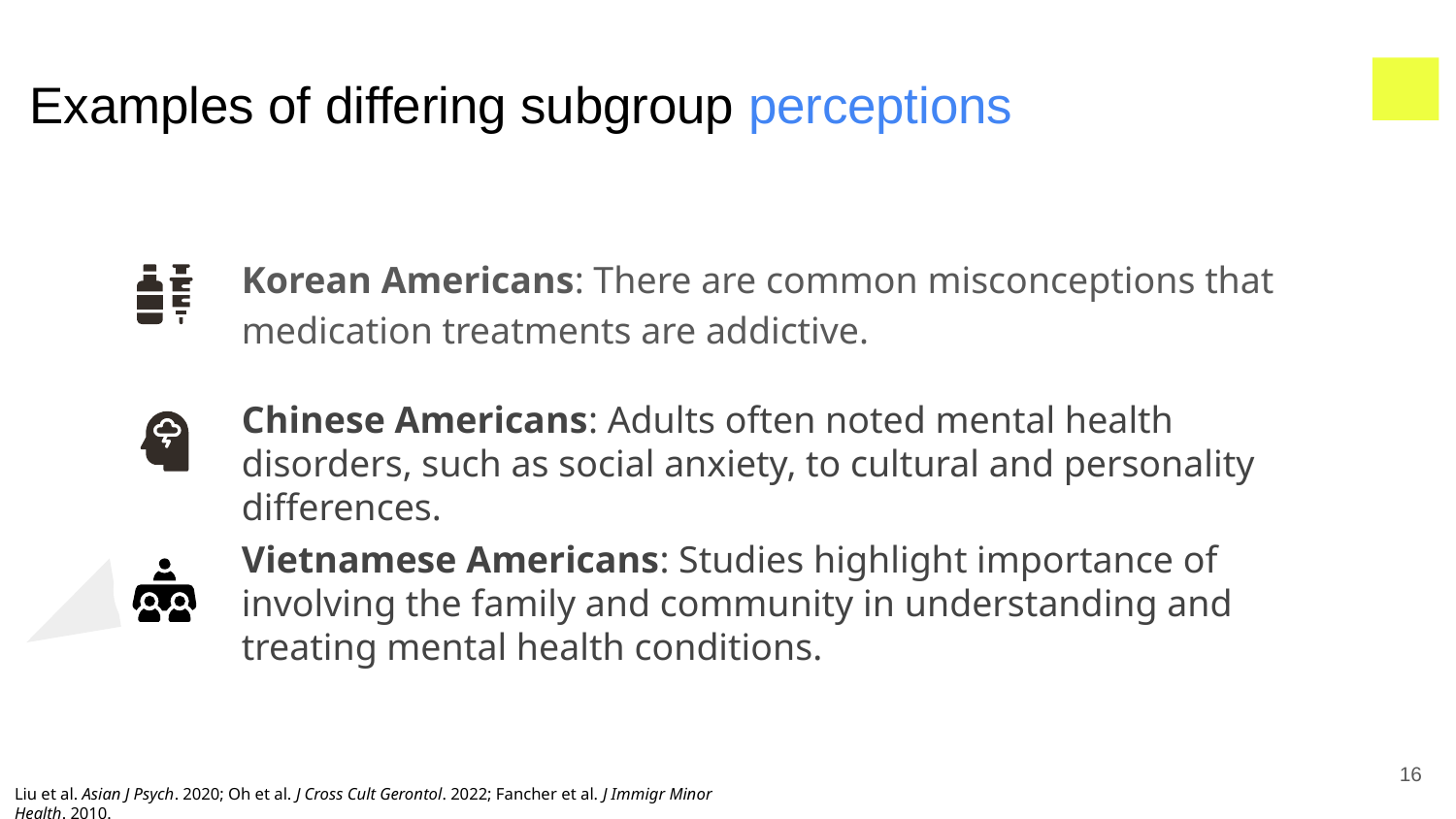

# Examples of differing subgroup perceptions
Korean Americans: There are common misconceptions that medication treatments are addictive.
Chinese Americans: Adults often noted mental health disorders, such as social anxiety, to cultural and personality differences.
Vietnamese Americans: Studies highlight importance of involving the family and community in understanding and treating mental health conditions.
‹#›
Liu et al. Asian J Psych. 2020; Oh et al. J Cross Cult Gerontol. 2022; Fancher et al. J Immigr Minor Health. 2010.

## Slide 17
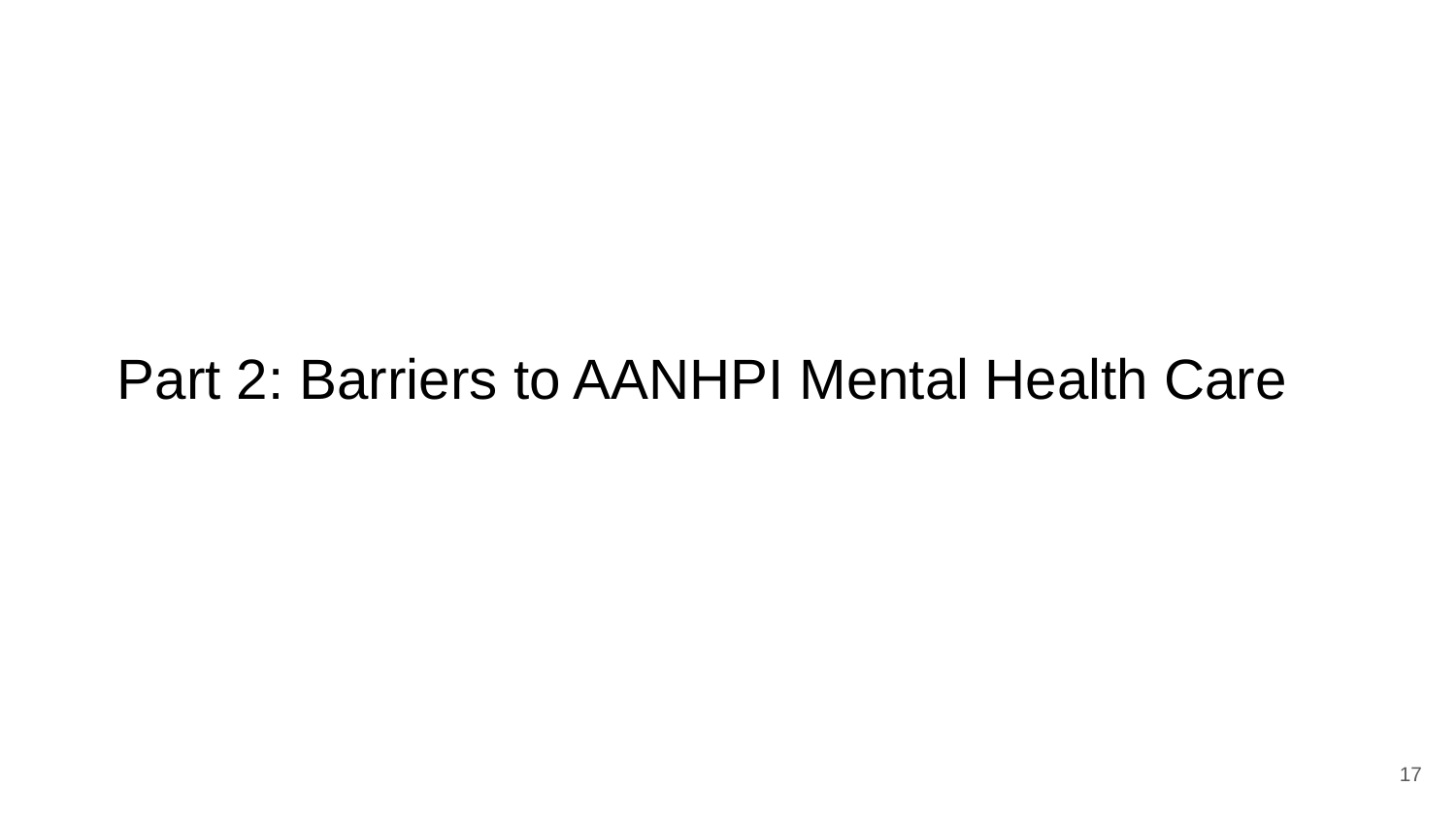

# Part 2: Barriers to AANHPI Mental Health Care
‹#›

## Slide 18
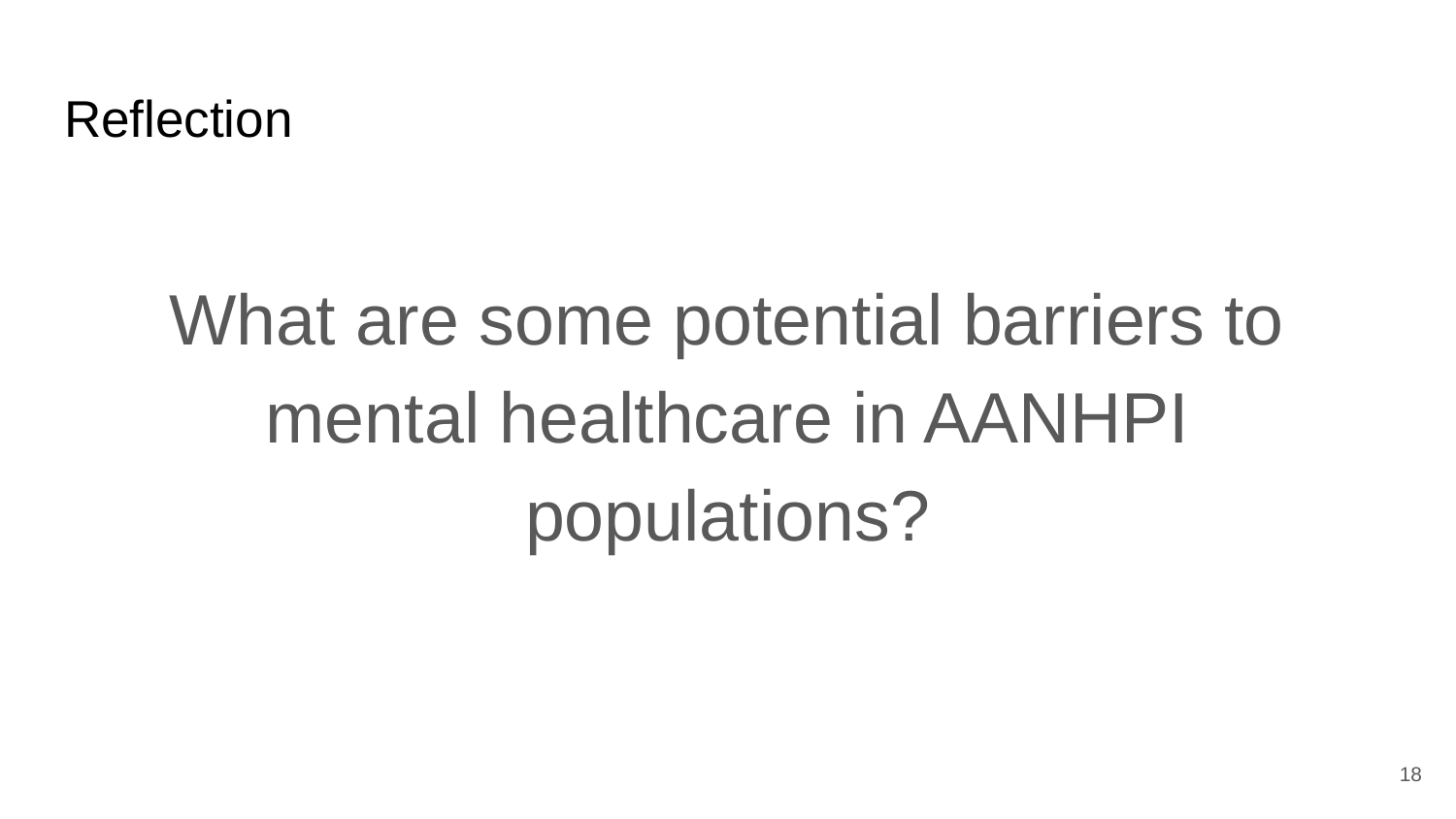

# Reflection
What are some potential barriers to mental healthcare in AANHPI populations?
‹#›

## Slide 19
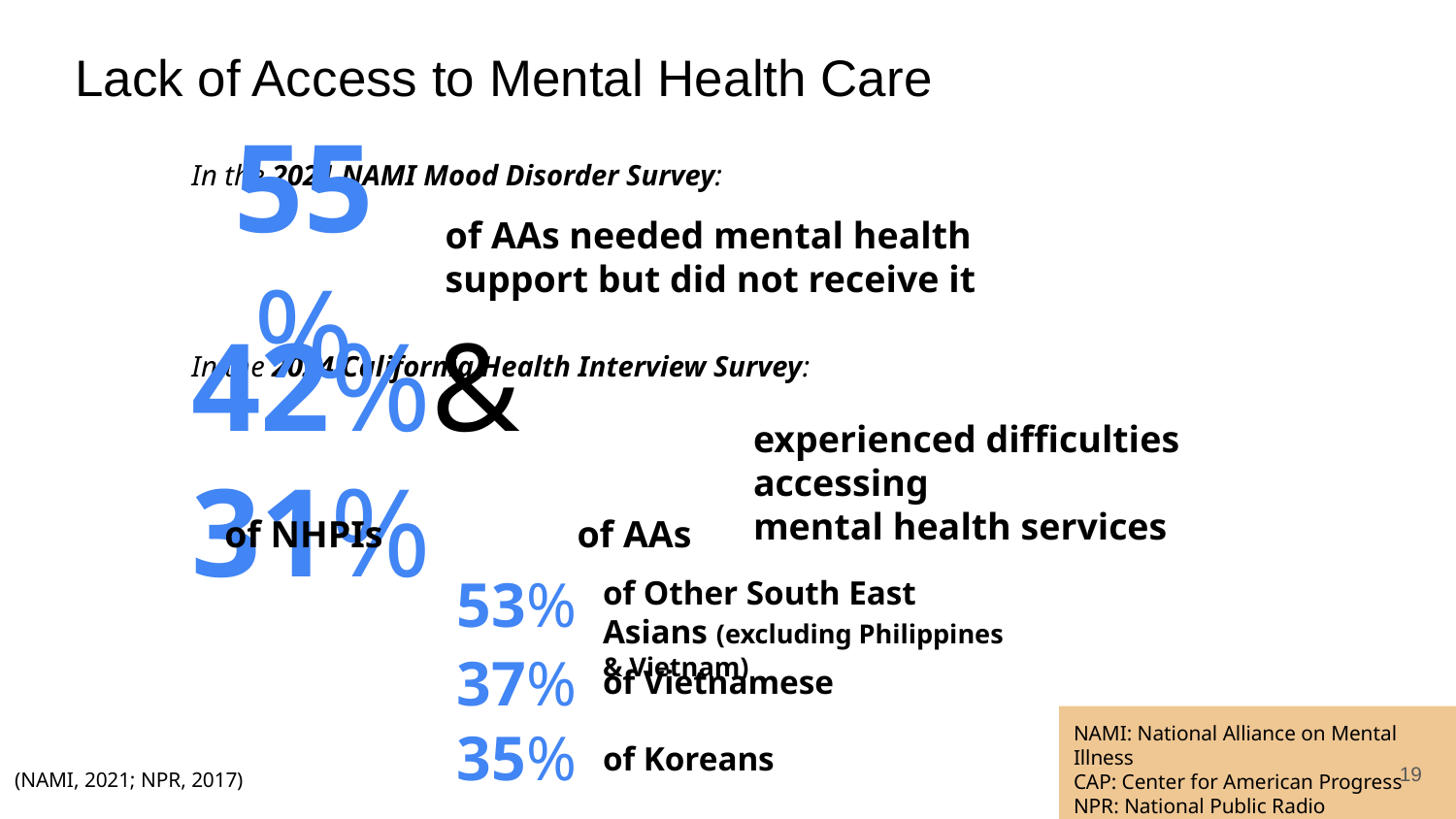

# Lack of Access to Mental Health Care
In the 2021 NAMI Mood Disorder Survey:
55%
of AAs needed mental health support but did not receive it
42%& 31%
In the 2024 California Health Interview Survey:
experienced difficulties accessing
mental health services
of AAs
of NHPIs
53%
37%
35%
of Other South East Asians (excluding Philippines & Vietnam)
of Vietnamese
of Koreans
NAMI: National Alliance on Mental Illness
CAP: Center for American Progress
NPR: National Public Radio
‹#›
(NAMI, 2021; NPR, 2017)

## Slide 20
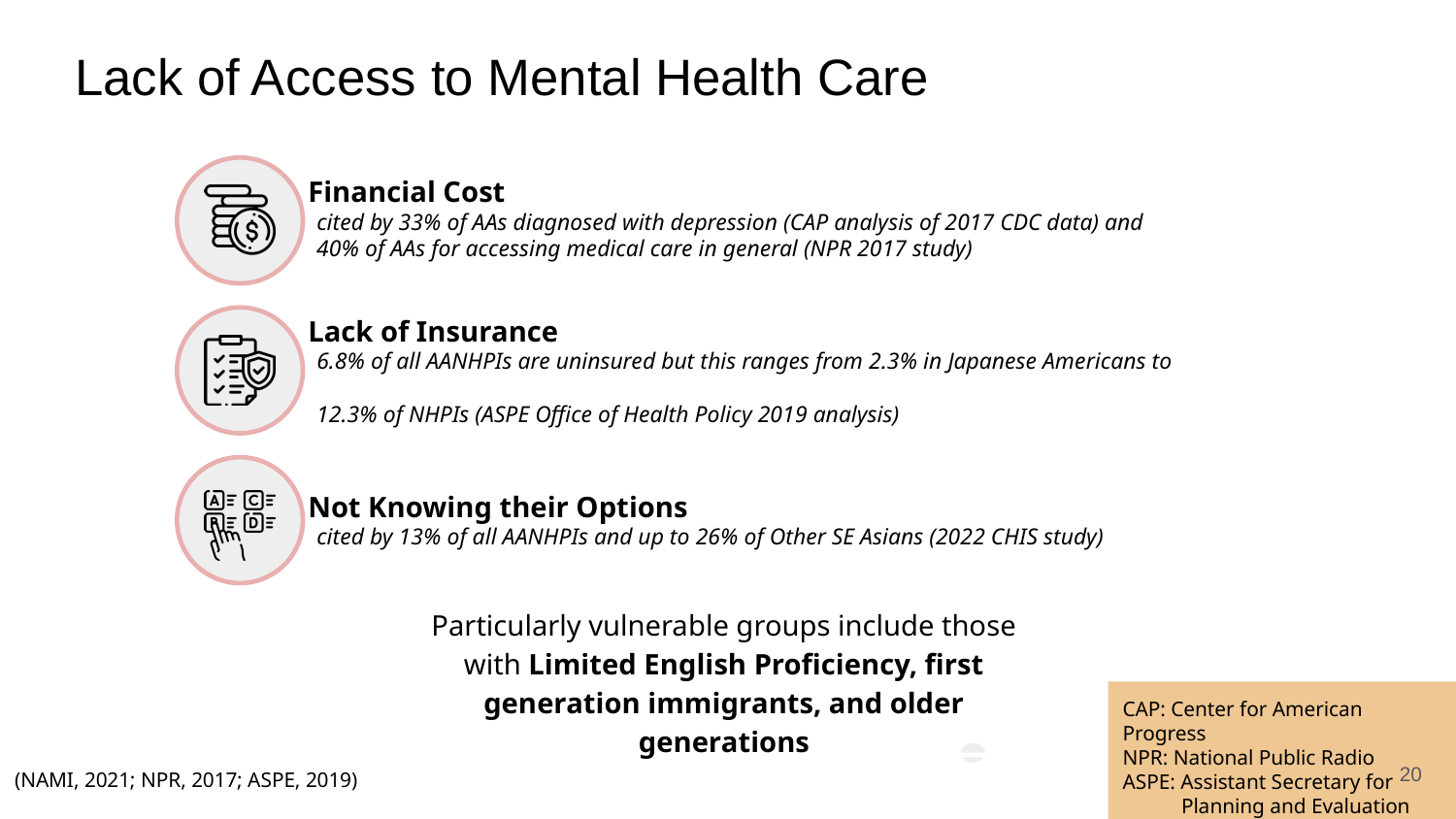

# Lack of Access to Mental Health Care
 Financial Cost
 cited by 33% of AAs diagnosed with depression (CAP analysis of 2017 CDC data) and
 40% of AAs for accessing medical care in general (NPR 2017 study)
 Lack of Insurance
 6.8% of all AANHPIs are uninsured but this ranges from 2.3% in Japanese Americans to
 12.3% of NHPIs (ASPE Office of Health Policy 2019 analysis)
 Not Knowing their Options
 cited by 13% of all AANHPIs and up to 26% of Other SE Asians (2022 CHIS study)
Particularly vulnerable groups include those with Limited English Proficiency, first generation immigrants, and older generations
CAP: Center for American Progress
NPR: National Public Radio
ASPE: Assistant Secretary for
 Planning and Evaluation
‹#›
(NAMI, 2021; NPR, 2017; ASPE, 2019)

## Slide 21
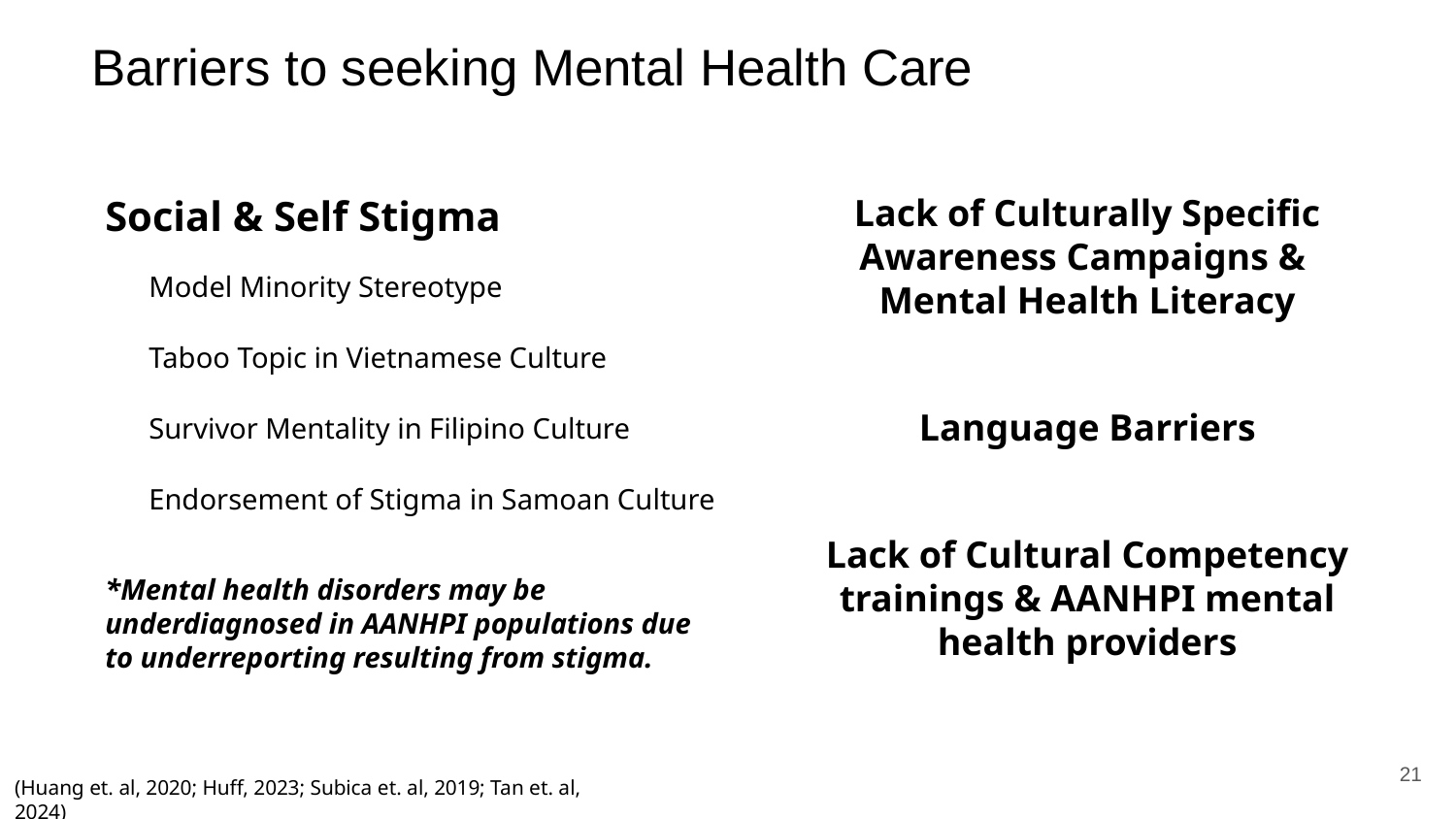

# Barriers to seeking Mental Health Care
Social & Self Stigma
*Mental health disorders may be underdiagnosed in AANHPI populations due to underreporting resulting from stigma.
Lack of Culturally Specific Awareness Campaigns &
Mental Health Literacy
Model Minority Stereotype
Taboo Topic in Vietnamese Culture
Language Barriers
Survivor Mentality in Filipino Culture
Endorsement of Stigma in Samoan Culture
Lack of Cultural Competency trainings & AANHPI mental health providers
‹#›
(Huang et. al, 2020; Huff, 2023; Subica et. al, 2019; Tan et. al, 2024)

## Slide 22
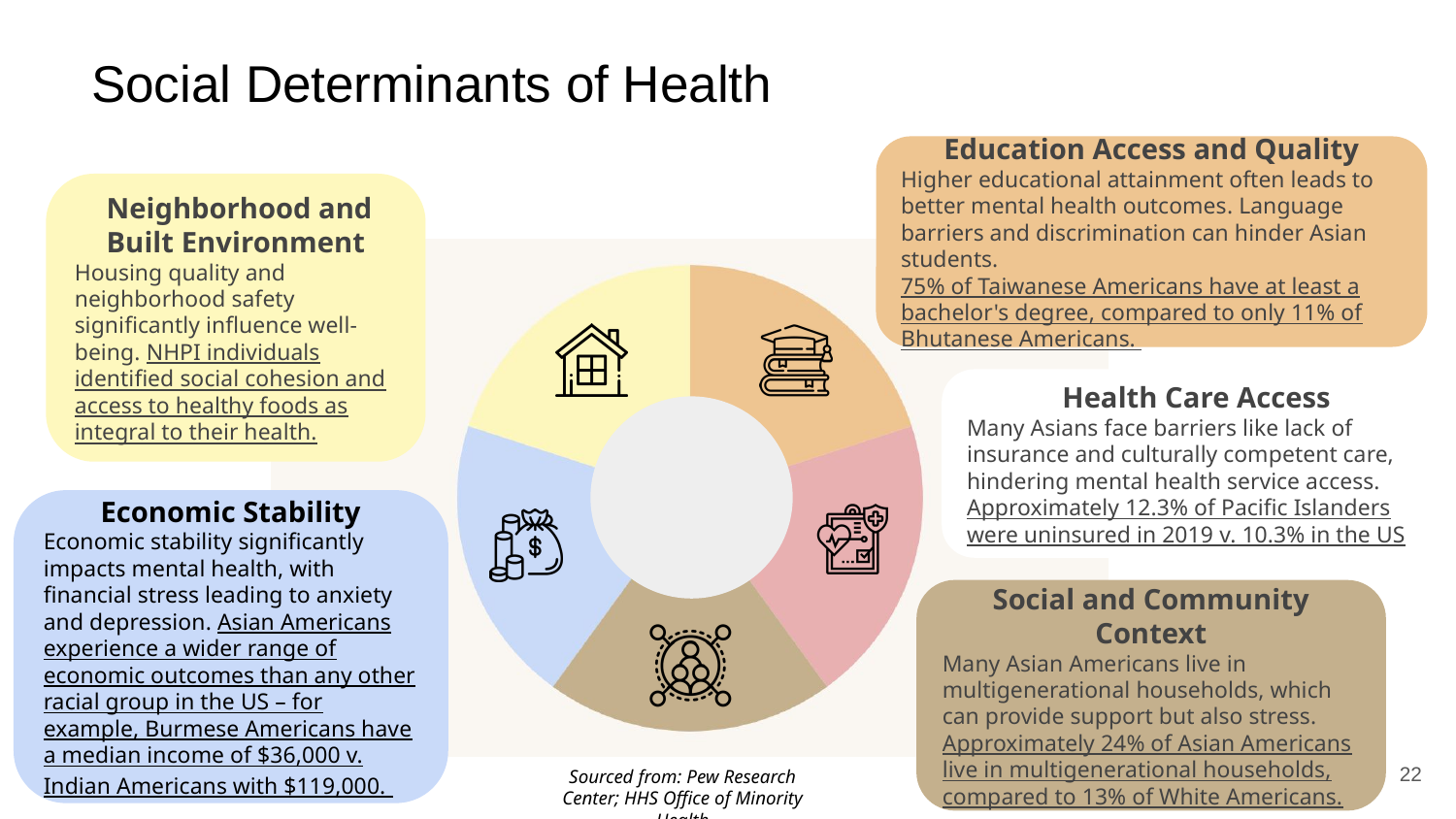

# Social Determinants of Health
Education Access and Quality
Higher educational attainment often leads to better mental health outcomes​. Language barriers and discrimination can hinder Asian students.
75% of Taiwanese Americans have at least a bachelor's degree, compared to only 11% of Bhutanese Americans.
 Neighborhood and Built Environment
Housing quality and neighborhood safety significantly influence well-being. NHPI individuals identified social cohesion and access to healthy foods as integral to their health.
Health Care Access
Many Asians face barriers like lack of insurance and culturally competent care, hindering mental health service access. Approximately 12.3% of Pacific Islanders were uninsured in 2019​ v. 10.3% in the US
Economic Stability
Economic stability significantly impacts mental health, with financial stress leading to anxiety and depression. Asian Americans experience a wider range of economic outcomes than any other racial group in the US – for example, Burmese Americans have a median income of $36,000 v. Indian Americans with $119,000.
Social and Community Context
Many Asian Americans live in multigenerational households, which can provide support but also stress​.
Approximately 24% of Asian Americans live in multigenerational households, compared to 13% of White Americans.
‹#›
Sourced from: Pew Research Center; HHS Office of Minority Health

## Slide 23
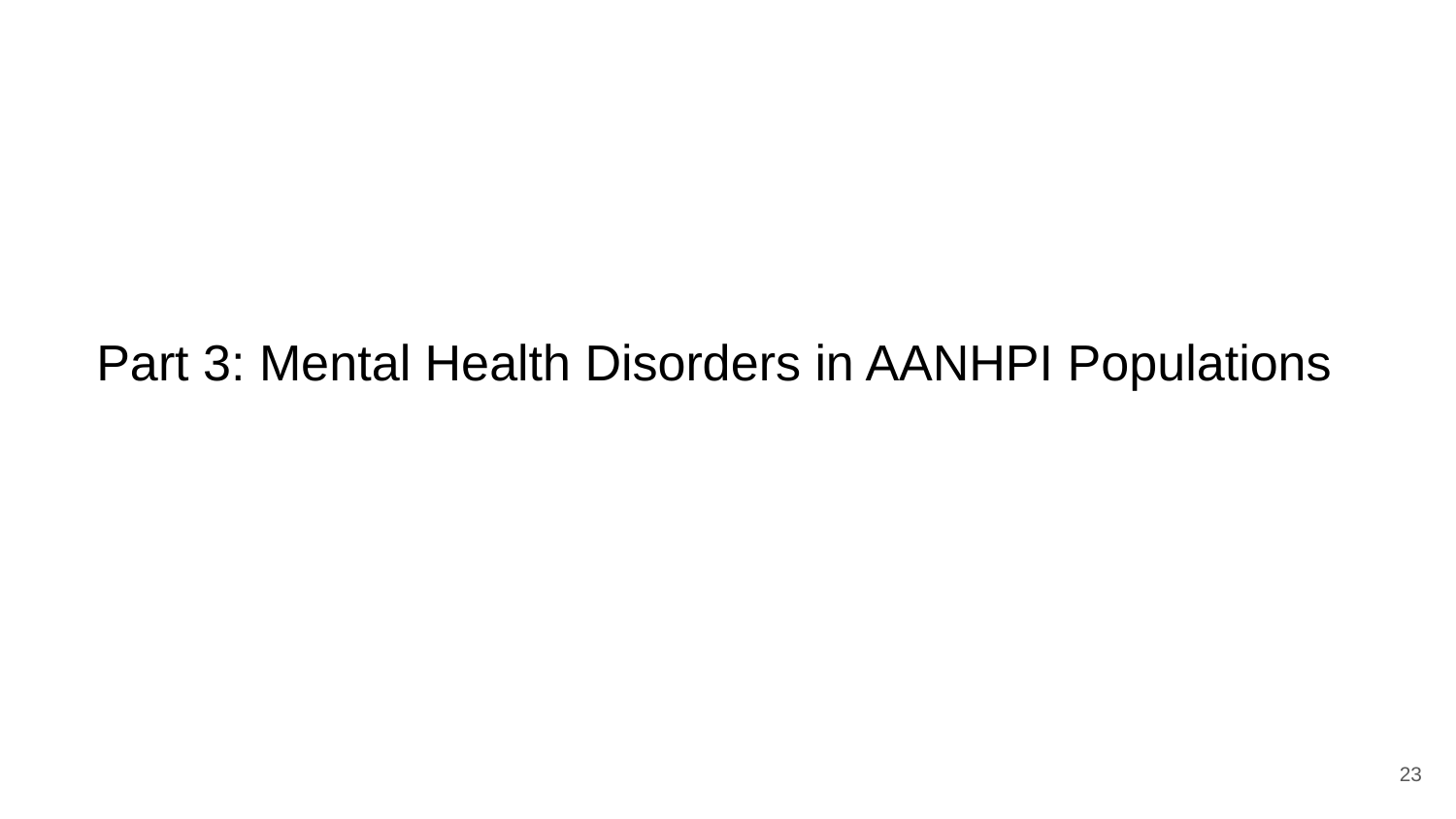

# Part 3: Mental Health Disorders in AANHPI Populations
‹#›

## Slide 24
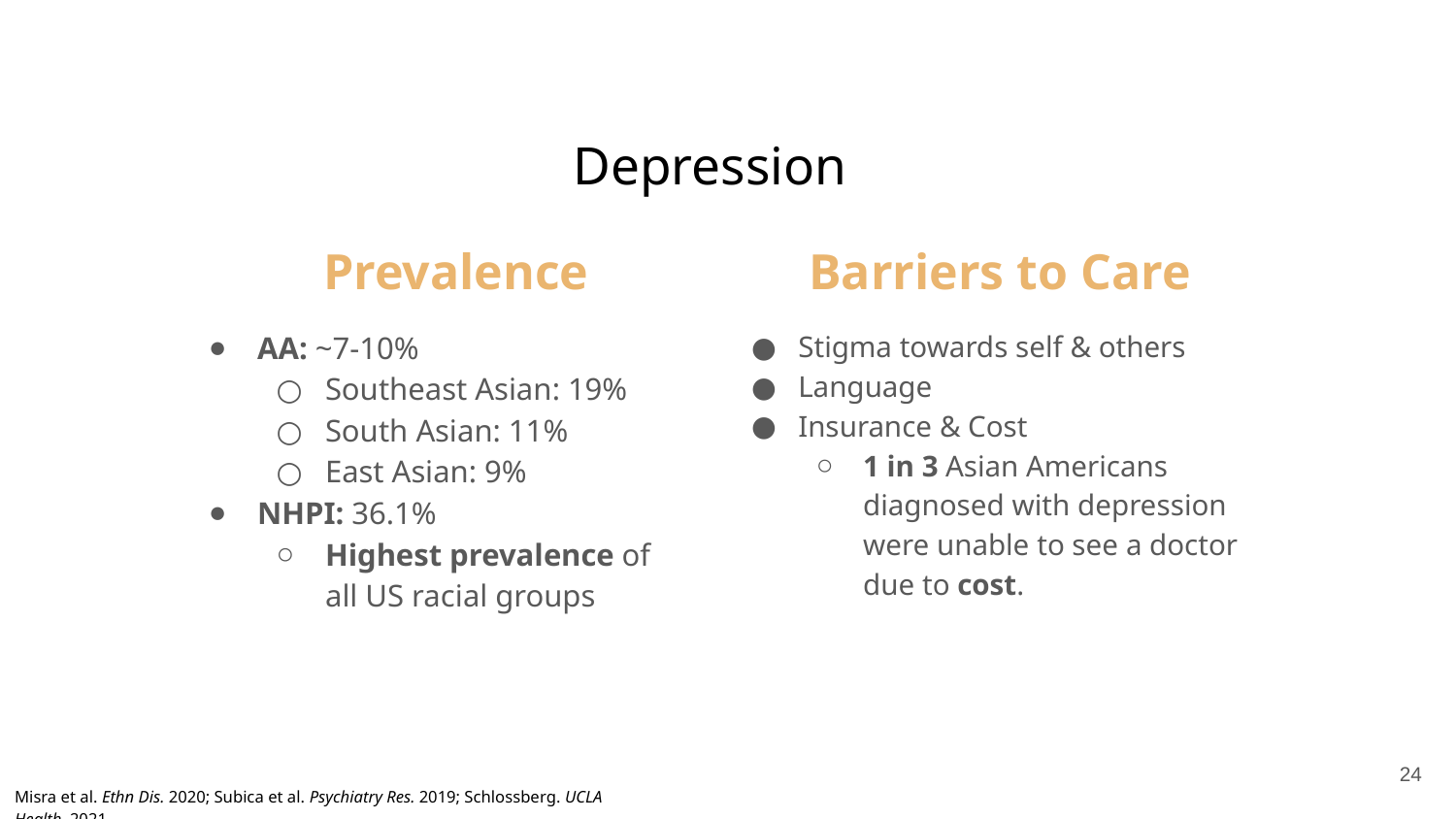

# Depression
Prevalence
Barriers to Care
AA: ~7-10%
Southeast Asian: 19%
South Asian: 11%
East Asian: 9%
NHPI: 36.1%
Highest prevalence of all US racial groups
Stigma towards self & others
Language
Insurance & Cost
1 in 3 Asian Americans diagnosed with depression were unable to see a doctor due to cost.
‹#›
Misra et al. Ethn Dis. 2020; Subica et al. Psychiatry Res. 2019; Schlossberg. UCLA Health. 2021.

## Slide 25
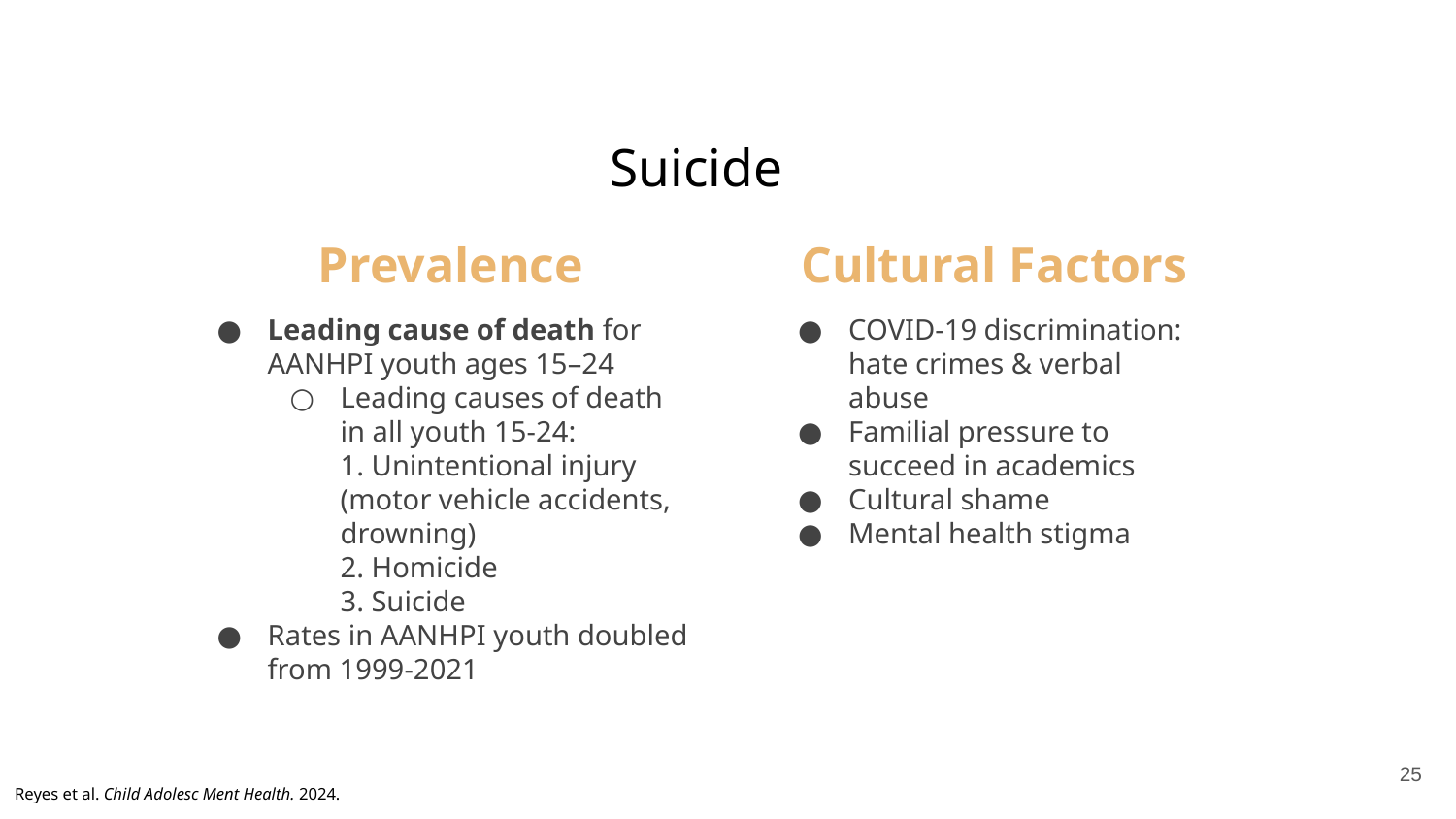

# Suicide
Prevalence
Cultural Factors
Leading cause of death for AANHPI youth ages 15–24
Leading causes of death in all youth 15-24:
1. Unintentional injury (motor vehicle accidents, drowning)
2. Homicide
3. Suicide
Rates in AANHPI youth doubled from 1999-2021
COVID-19 discrimination: hate crimes & verbal abuse
Familial pressure to succeed in academics
Cultural shame
Mental health stigma
‹#›
Reyes et al. Child Adolesc Ment Health. 2024.

## Slide 26
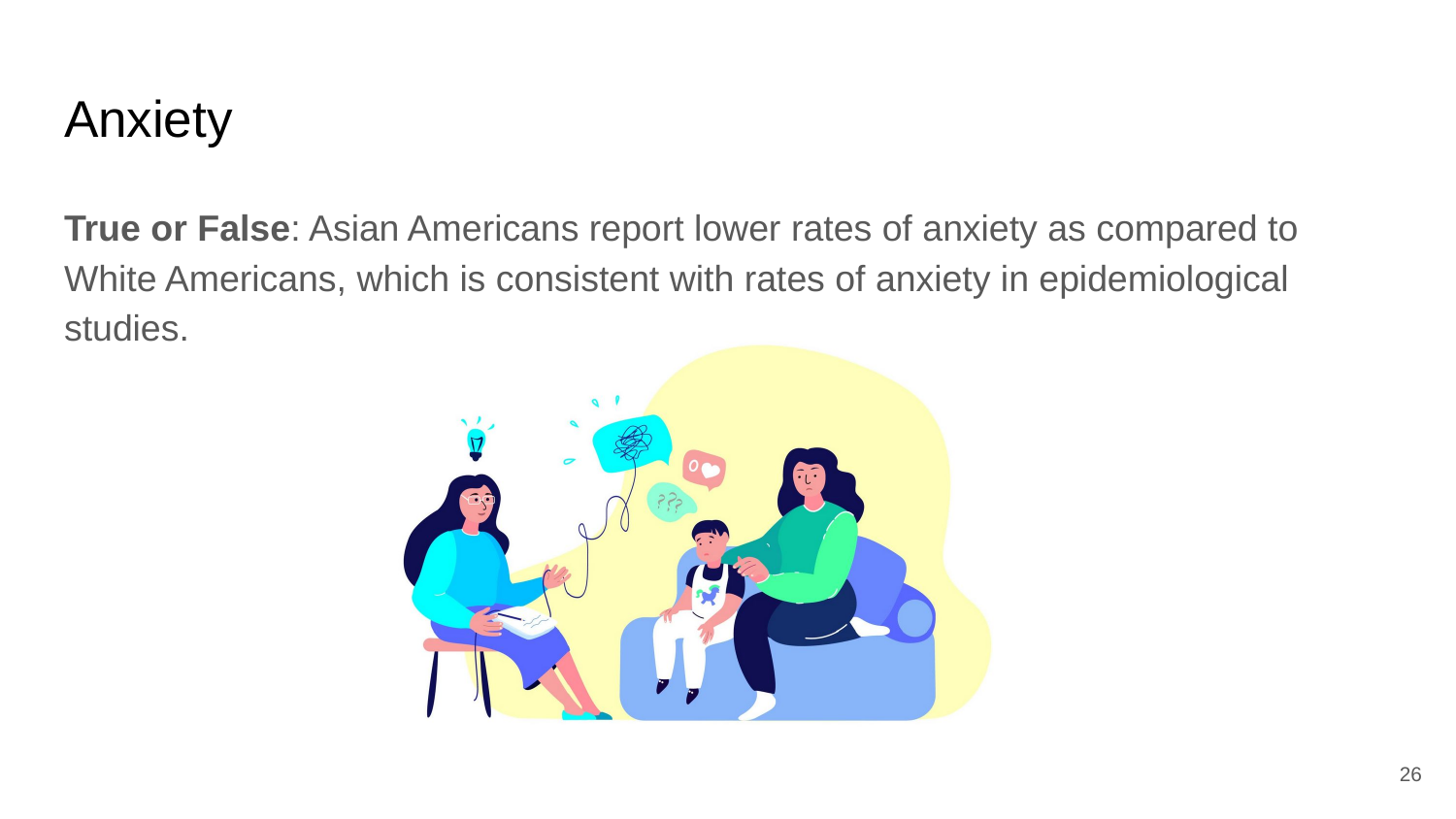

# Anxiety
True or False: Asian Americans report lower rates of anxiety as compared to White Americans, which is consistent with rates of anxiety in epidemiological studies.
‹#›

## Slide 27
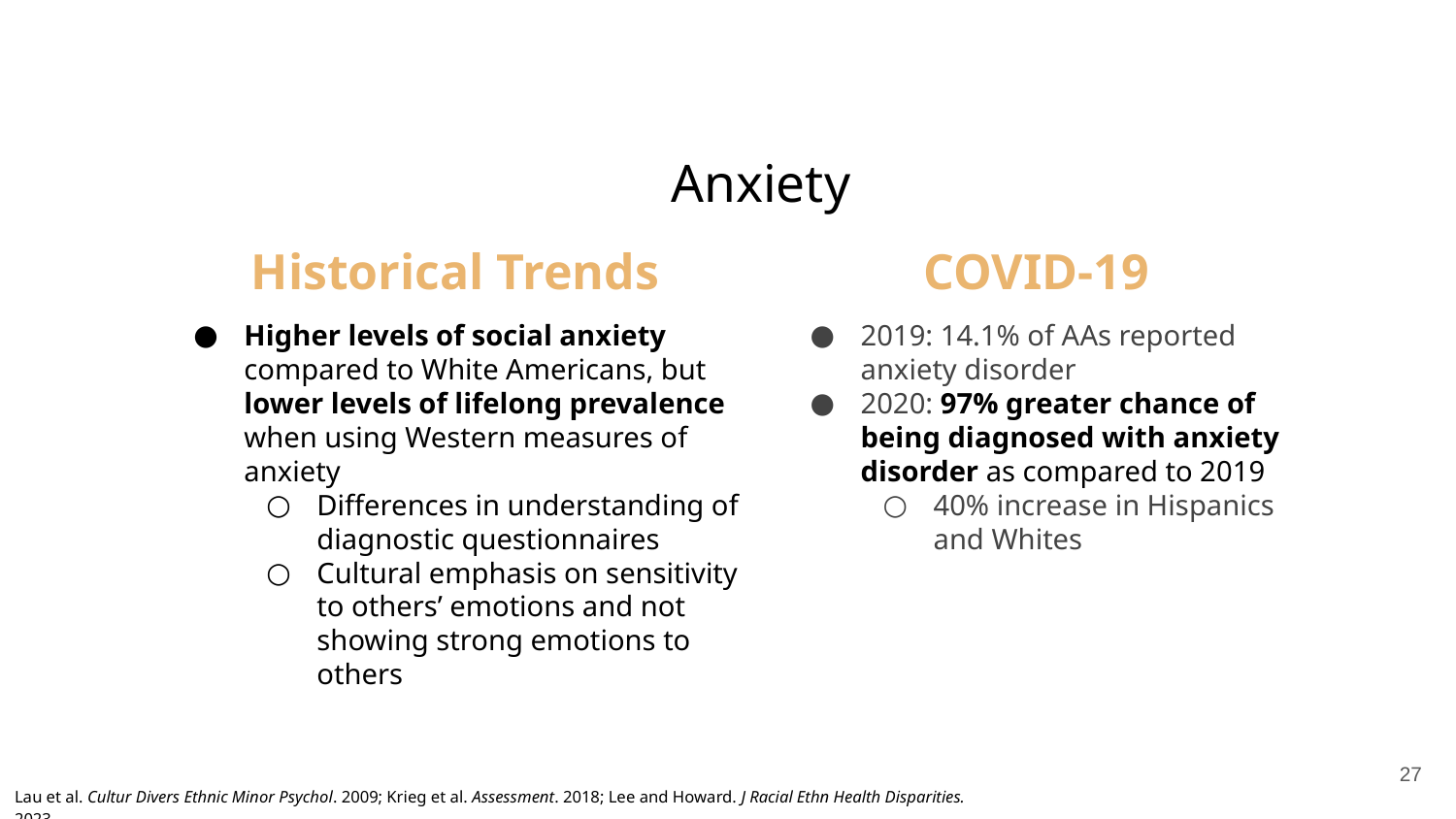

# Anxiety
COVID-19
Historical Trends
Higher levels of social anxiety compared to White Americans, but lower levels of lifelong prevalence when using Western measures of anxiety
Differences in understanding of diagnostic questionnaires
Cultural emphasis on sensitivity to others’ emotions and not showing strong emotions to others
2019: 14.1% of AAs reported anxiety disorder
2020: 97% greater chance of being diagnosed with anxiety disorder as compared to 2019
40% increase in Hispanics and Whites
‹#›
Lau et al. Cultur Divers Ethnic Minor Psychol. 2009; Krieg et al. Assessment. 2018; Lee and Howard. J Racial Ethn Health Disparities. 2023.

## Slide 28
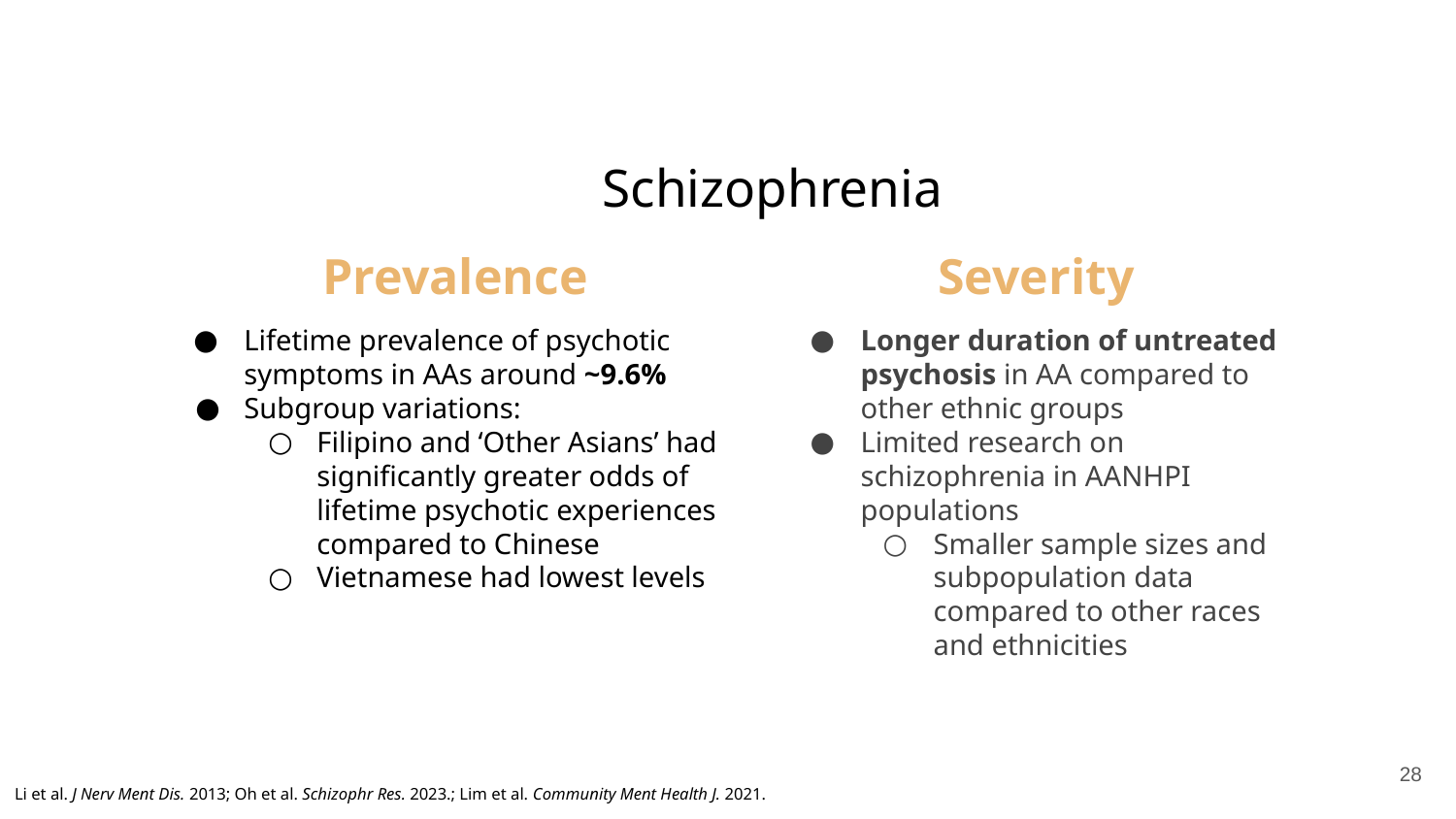

# Schizophrenia
Severity
Prevalence
Lifetime prevalence of psychotic symptoms in AAs around ~9.6%
Subgroup variations:
Filipino and ‘Other Asians’ had significantly greater odds of lifetime psychotic experiences compared to Chinese
Vietnamese had lowest levels
Longer duration of untreated psychosis in AA compared to other ethnic groups
Limited research on schizophrenia in AANHPI populations
Smaller sample sizes and subpopulation data compared to other races and ethnicities
‹#›
Li et al. J Nerv Ment Dis. 2013; Oh et al. Schizophr Res. 2023.; Lim et al. Community Ment Health J. 2021.

## Slide 29
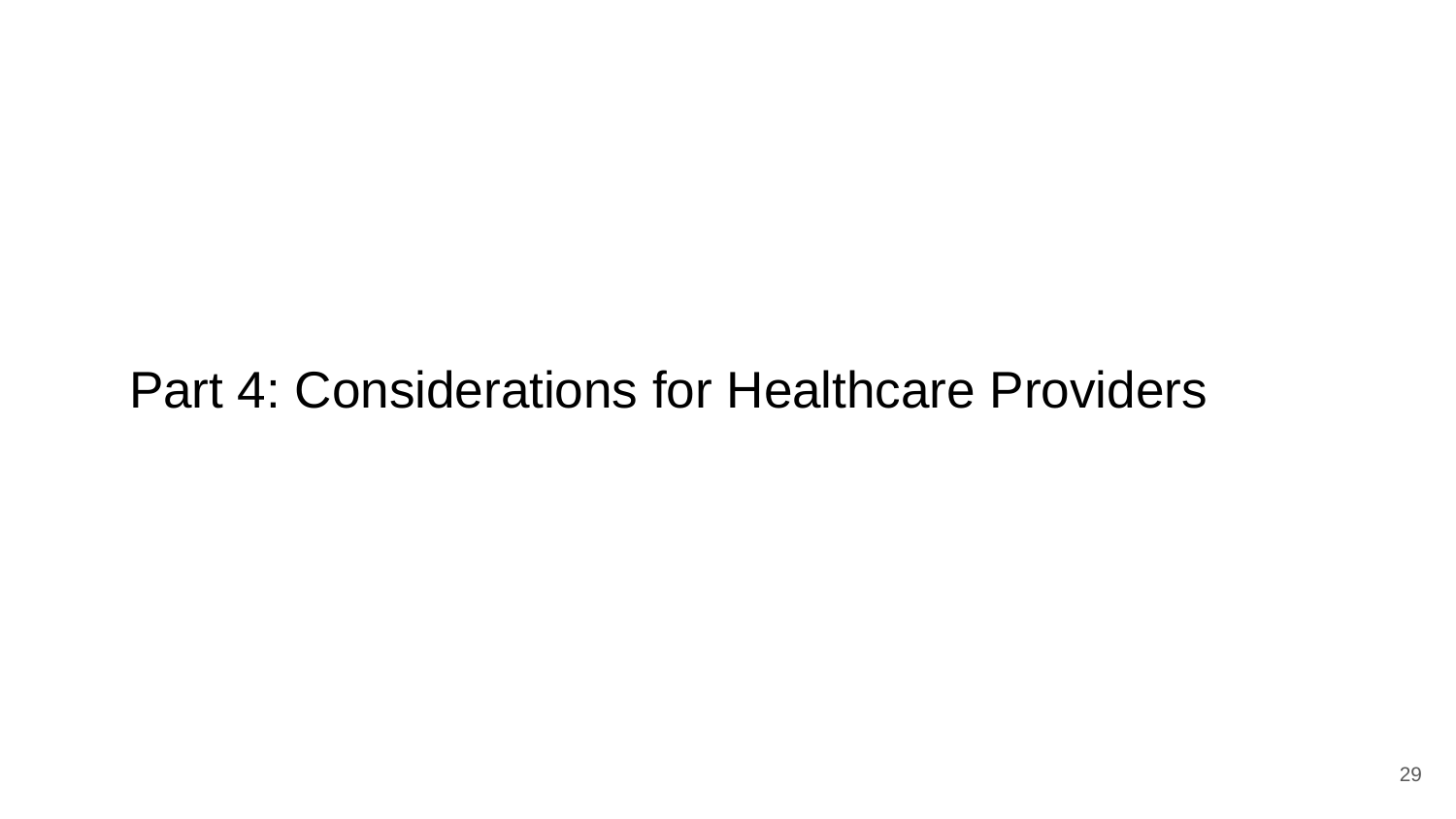

# Part 4: Considerations for Healthcare Providers
‹#›

## Slide 30
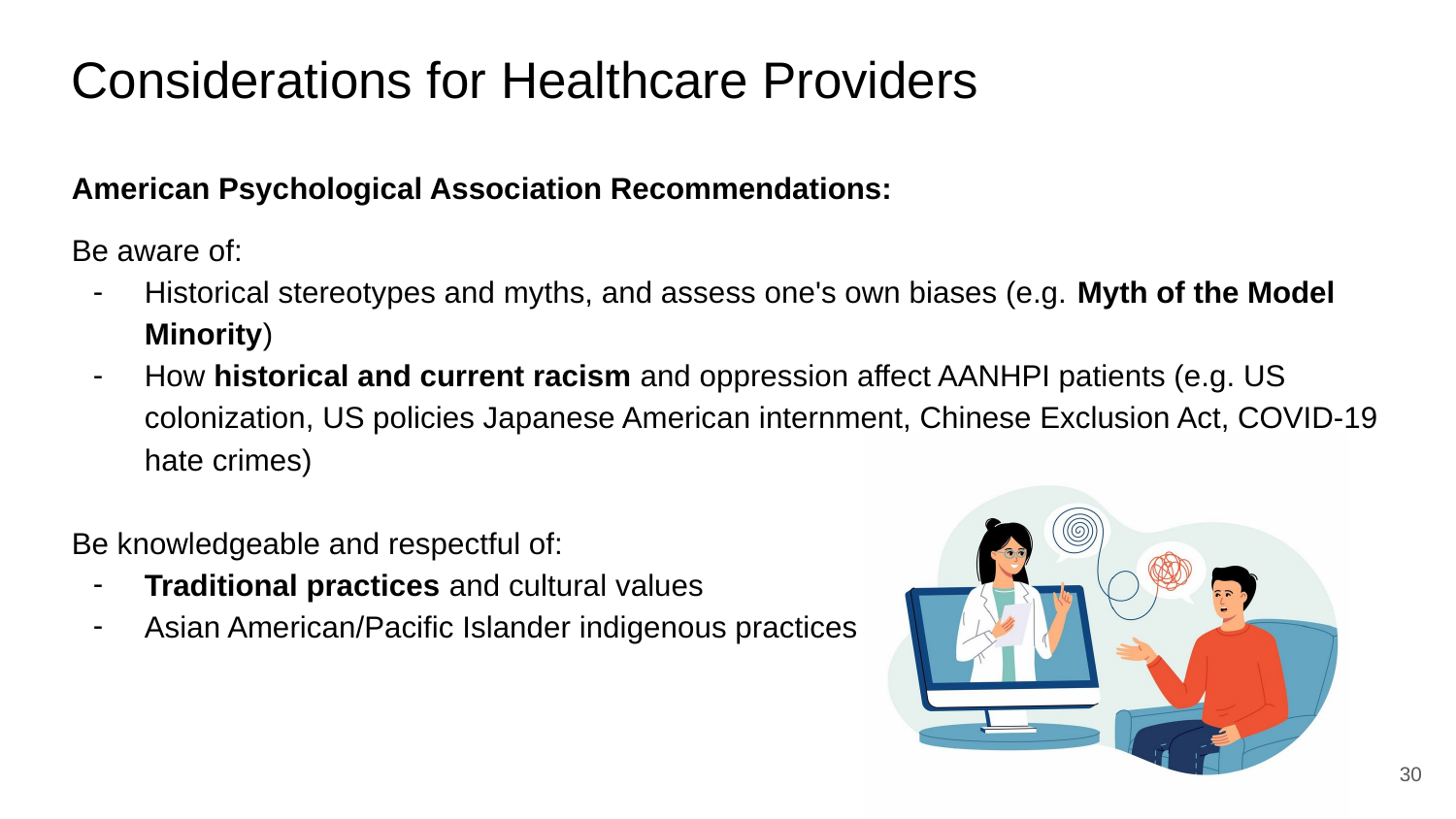

# Considerations for Healthcare Providers
American Psychological Association Recommendations:
Be aware of:
Historical stereotypes and myths, and assess one's own biases (e.g. Myth of the Model Minority)
How historical and current racism and oppression affect AANHPI patients (e.g. US colonization, US policies Japanese American internment, Chinese Exclusion Act, COVID-19 hate crimes)
Be knowledgeable and respectful of:
Traditional practices and cultural values
Asian American/Pacific Islander indigenous practices
‹#›

## Slide 31
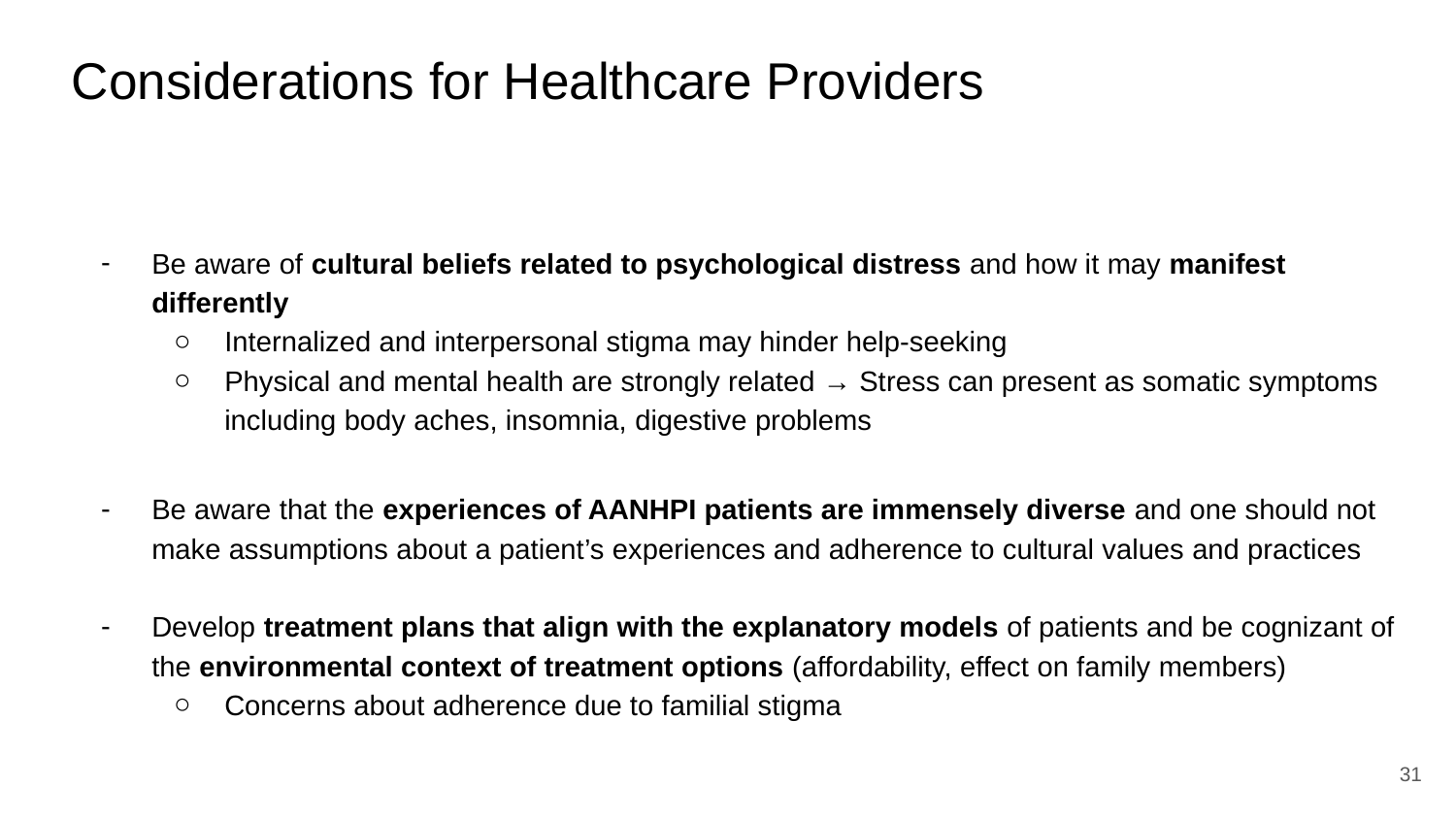

# Considerations for Healthcare Providers
Be aware of cultural beliefs related to psychological distress and how it may manifest differently
Internalized and interpersonal stigma may hinder help-seeking
Physical and mental health are strongly related → Stress can present as somatic symptoms including body aches, insomnia, digestive problems
Be aware that the experiences of AANHPI patients are immensely diverse and one should not make assumptions about a patient’s experiences and adherence to cultural values and practices
Develop treatment plans that align with the explanatory models of patients and be cognizant of the environmental context of treatment options (affordability, effect on family members)
Concerns about adherence due to familial stigma
‹#›

## Slide 32
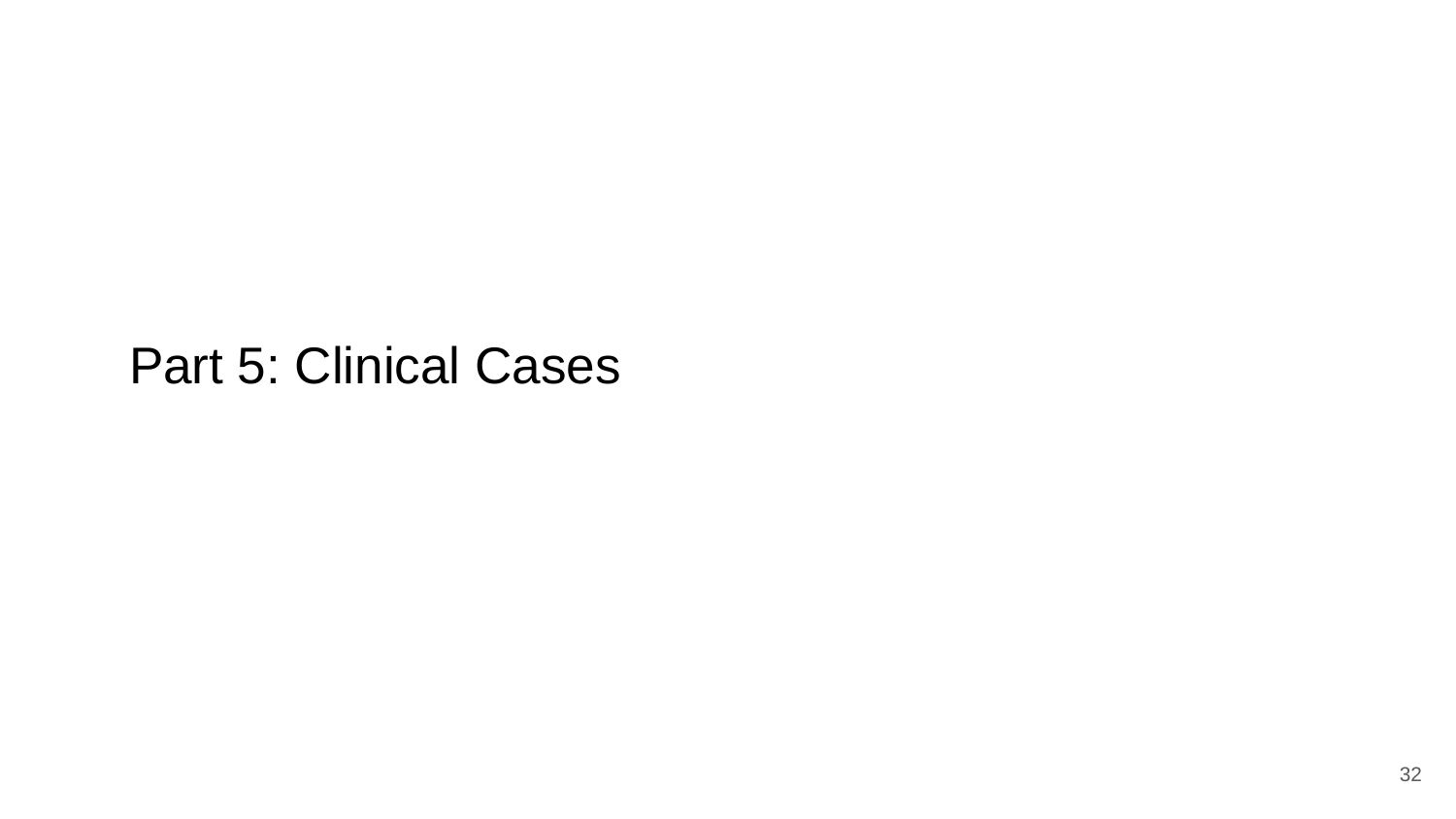

# Part 5: Clinical Cases
‹#›

## Slide 33
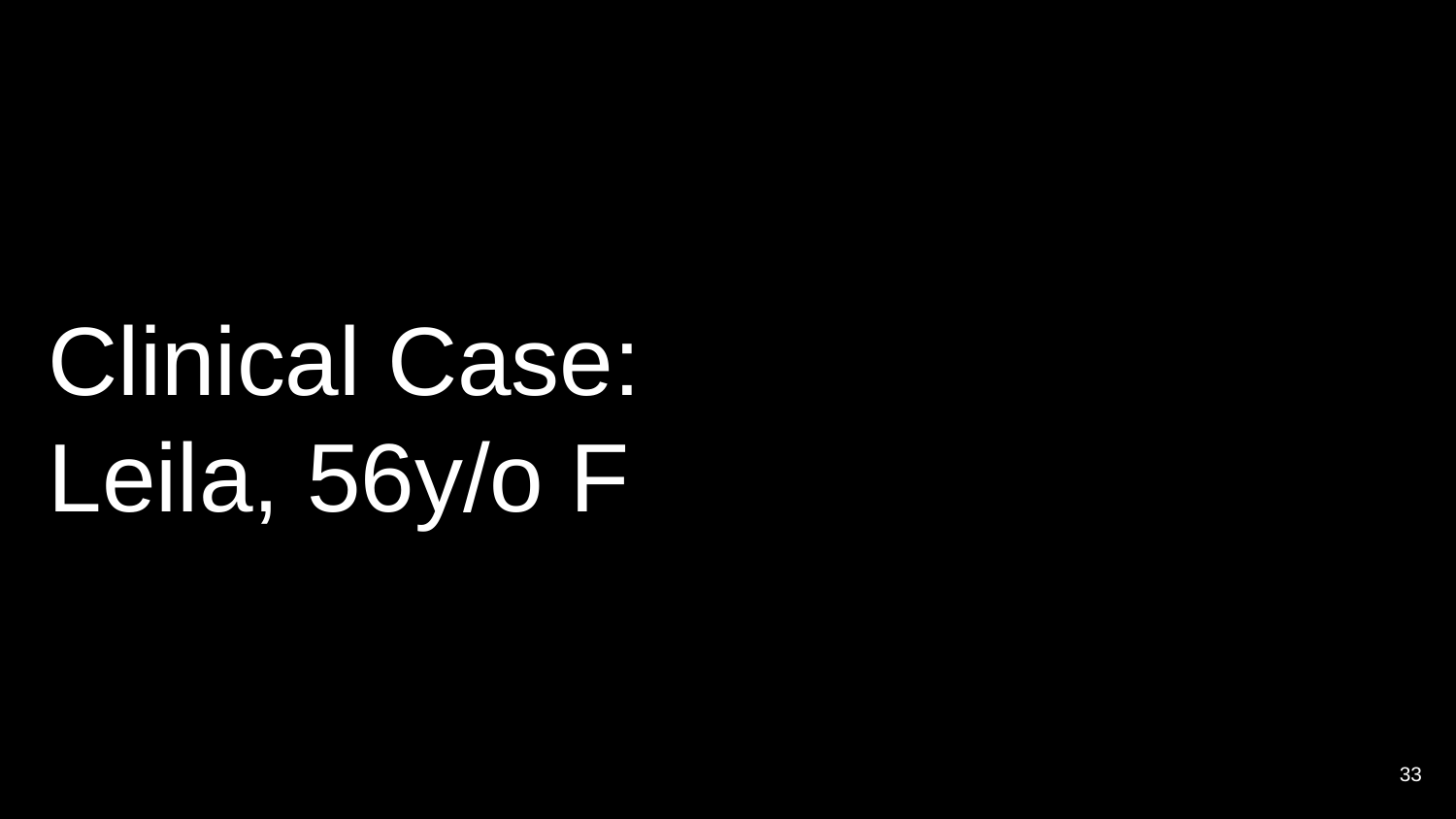

# Clinical Case:
Leila, 56y/o F
‹#›

## Slide 34
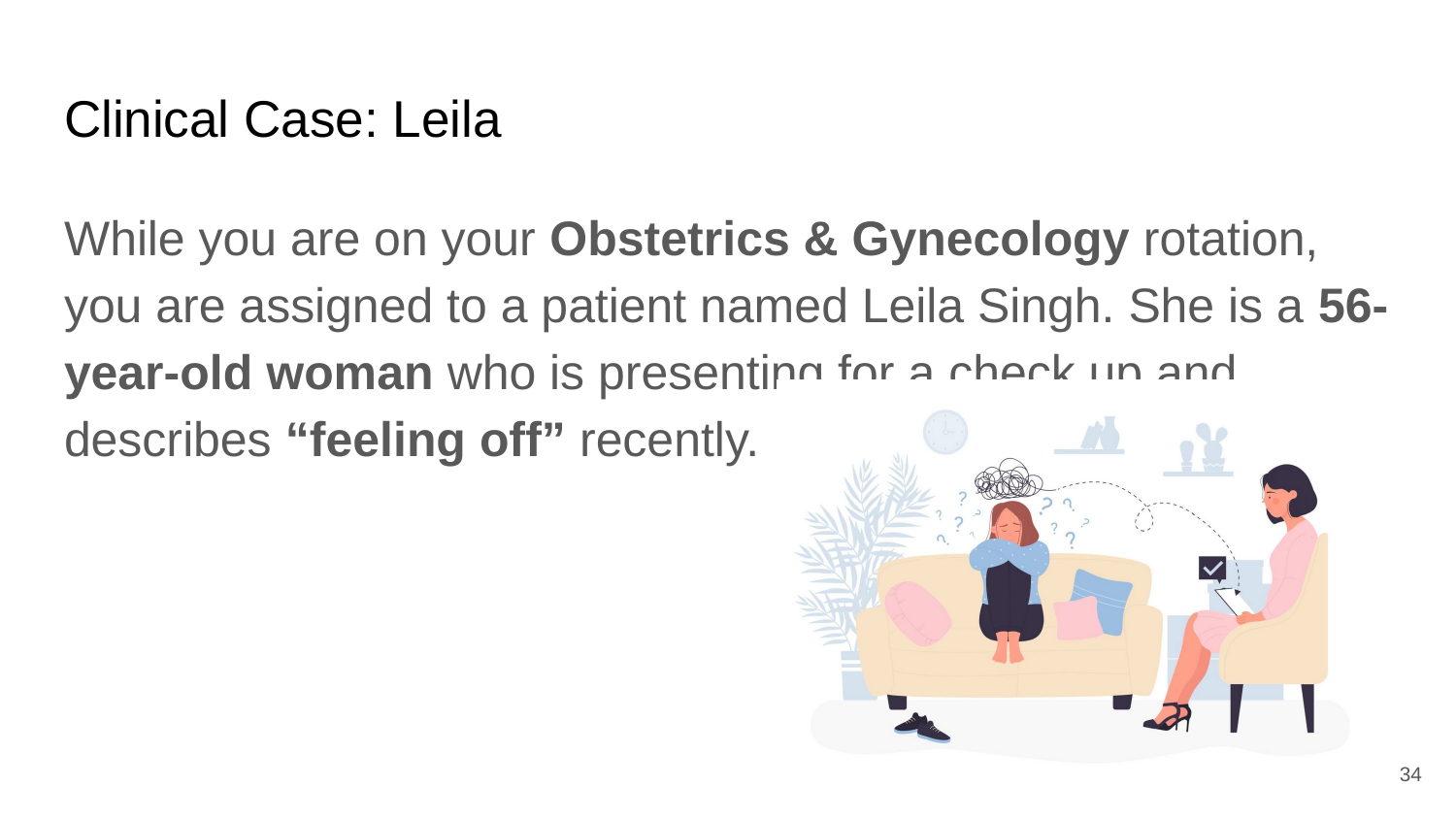

# Clinical Case: Leila
While you are on your Obstetrics & Gynecology rotation, you are assigned to a patient named Leila Singh. She is a 56-year-old woman who is presenting for a check up and describes “feeling off” recently.
‹#›

## Slide 35
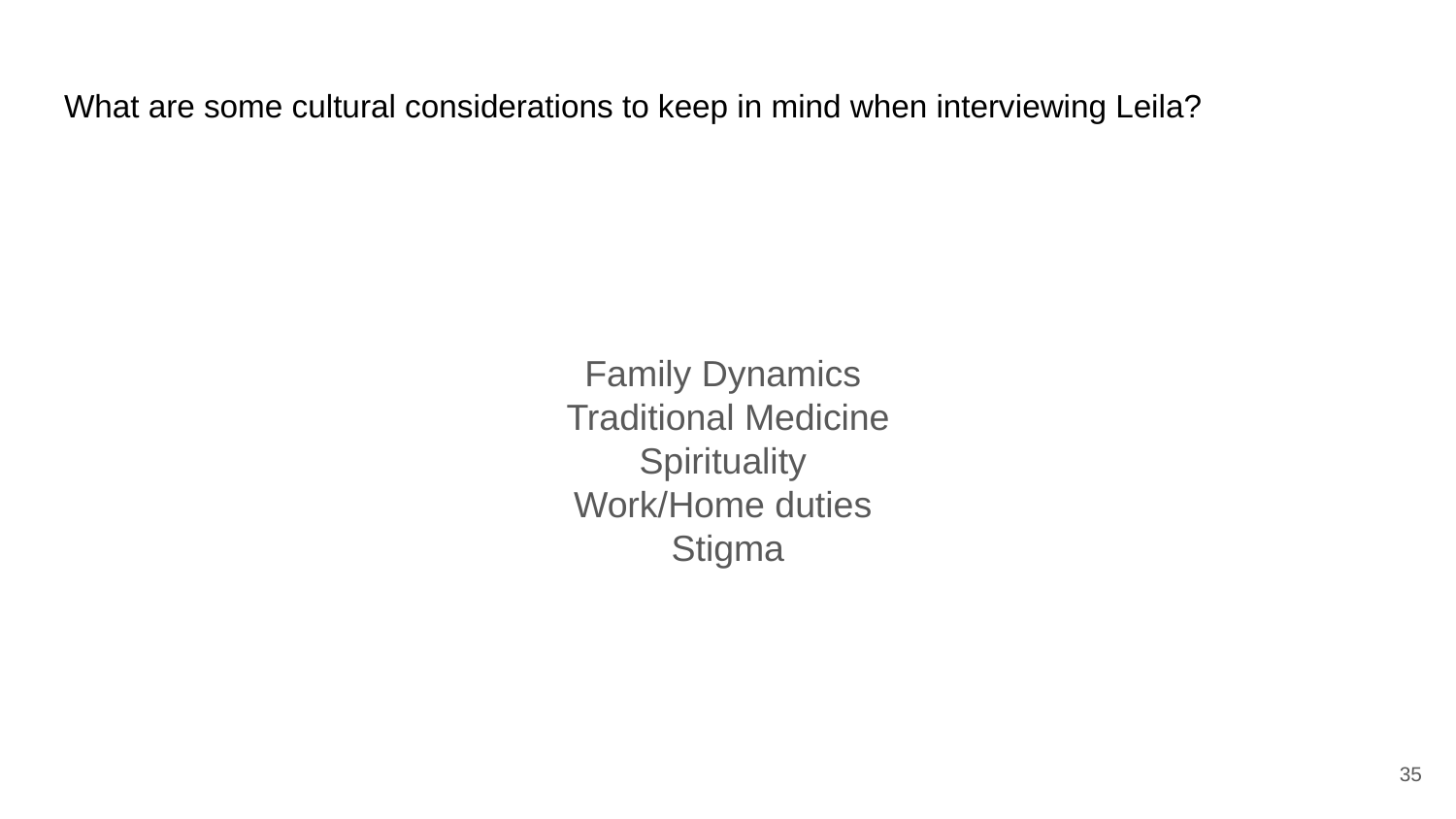

# What are some cultural considerations to keep in mind when interviewing Leila?
Family Dynamics
Traditional Medicine
Spirituality
Work/Home duties
Stigma
‹#›

## Slide 36
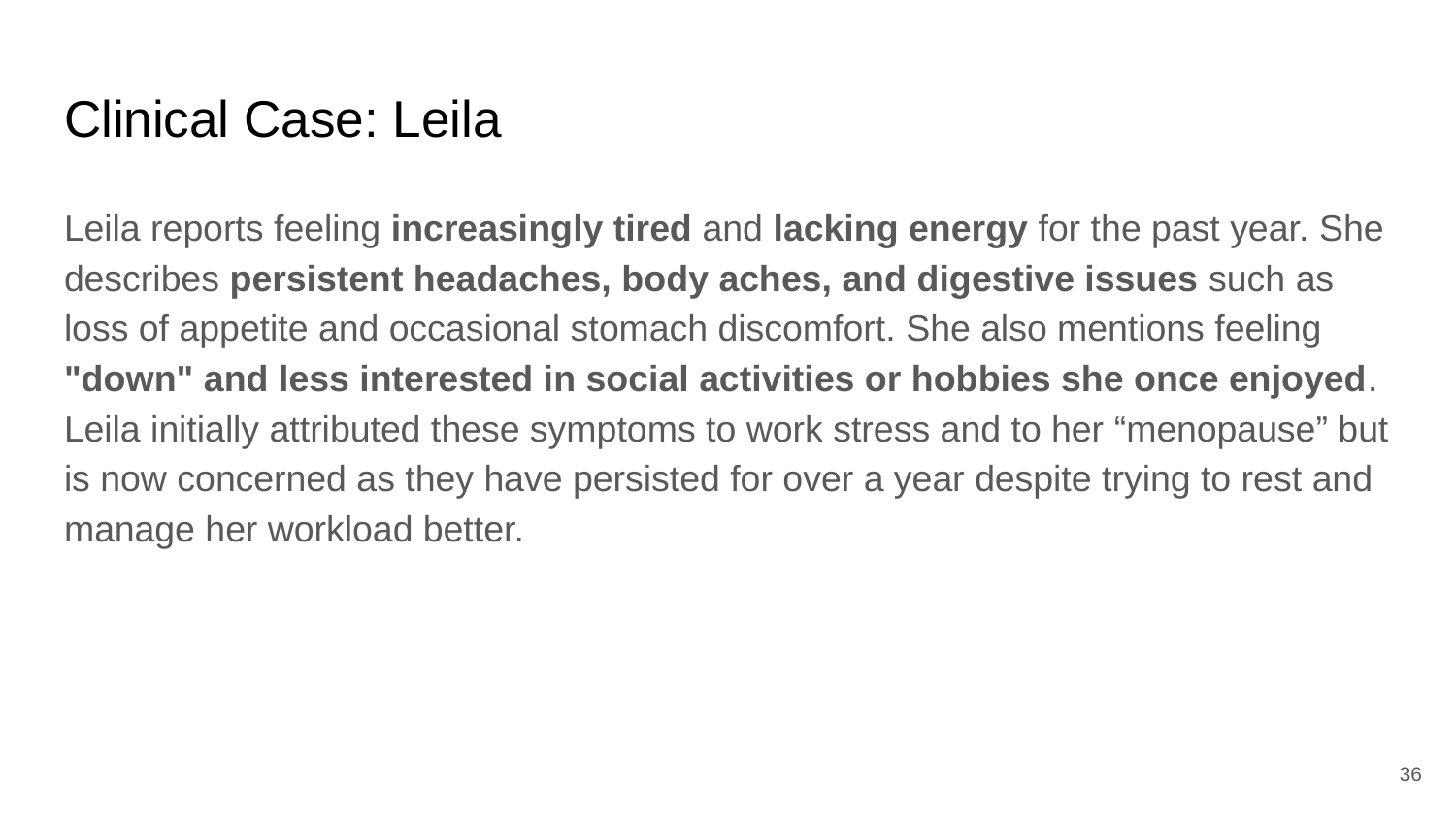

# Clinical Case: Leila
Leila reports feeling increasingly tired and lacking energy for the past year. She describes persistent headaches, body aches, and digestive issues such as loss of appetite and occasional stomach discomfort. She also mentions feeling "down" and less interested in social activities or hobbies she once enjoyed. Leila initially attributed these symptoms to work stress and to her “menopause” but is now concerned as they have persisted for over a year despite trying to rest and manage her workload better.
‹#›

## Slide 37
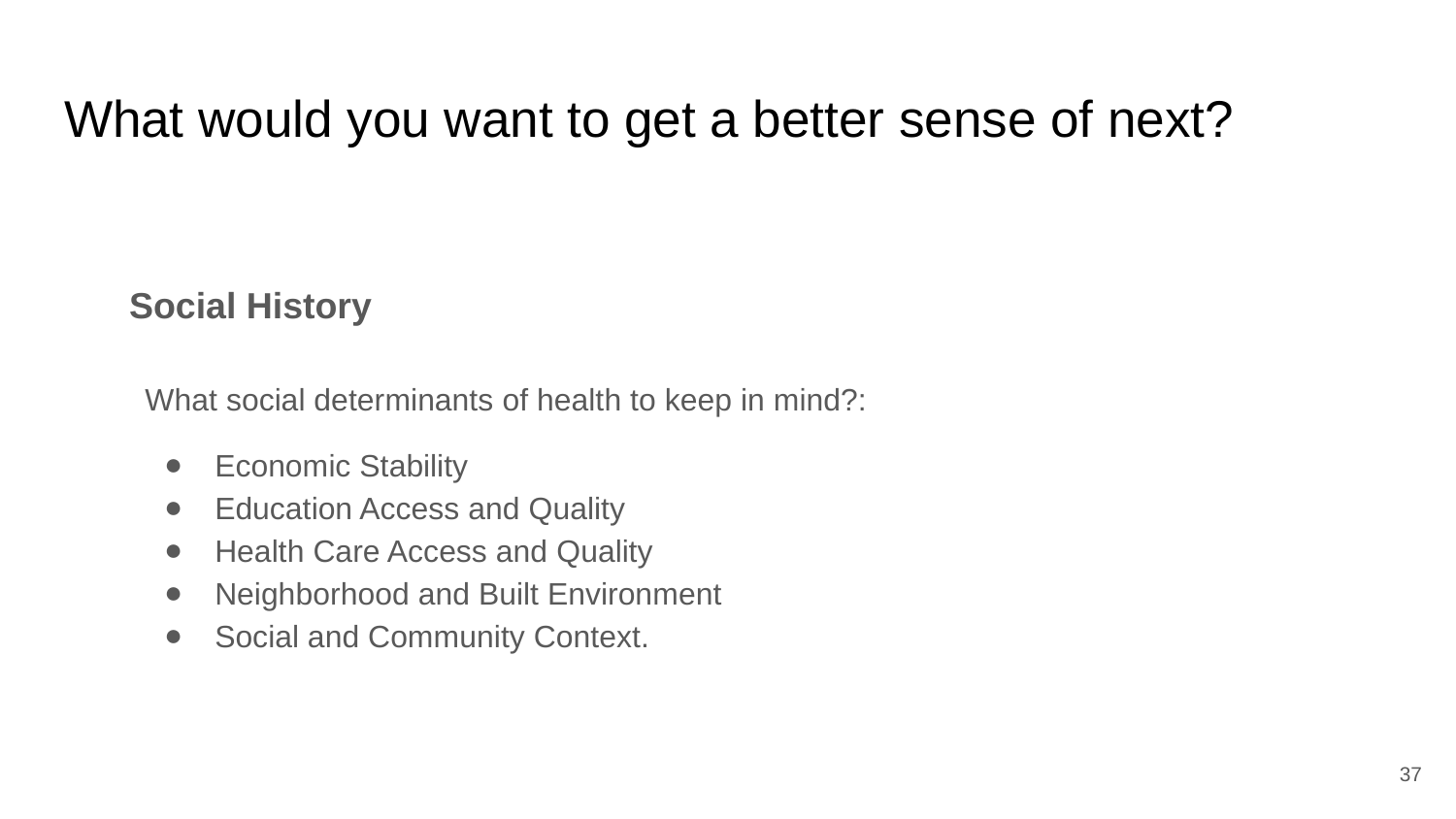

# What would you want to get a better sense of next?
Social History
What social determinants of health to keep in mind?:
Economic Stability
Education Access and Quality
Health Care Access and Quality
Neighborhood and Built Environment
Social and Community Context.
‹#›

## Slide 38
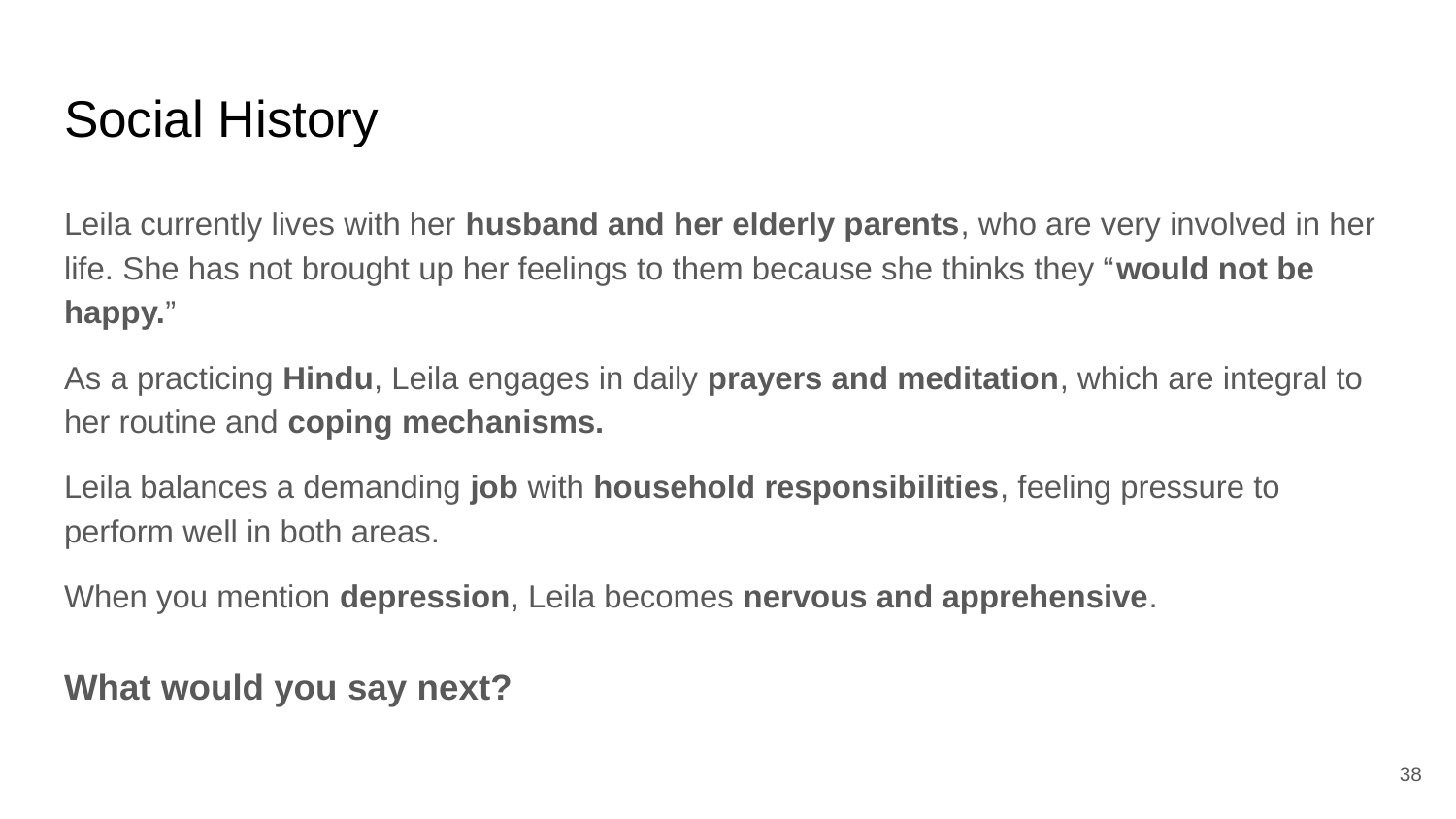

# Social History
Leila currently lives with her husband and her elderly parents, who are very involved in her life. She has not brought up her feelings to them because she thinks they “would not be happy.”
As a practicing Hindu, Leila engages in daily prayers and meditation, which are integral to her routine and coping mechanisms.
Leila balances a demanding job with household responsibilities, feeling pressure to perform well in both areas.
When you mention depression, Leila becomes nervous and apprehensive.
What would you say next?
‹#›

## Slide 39
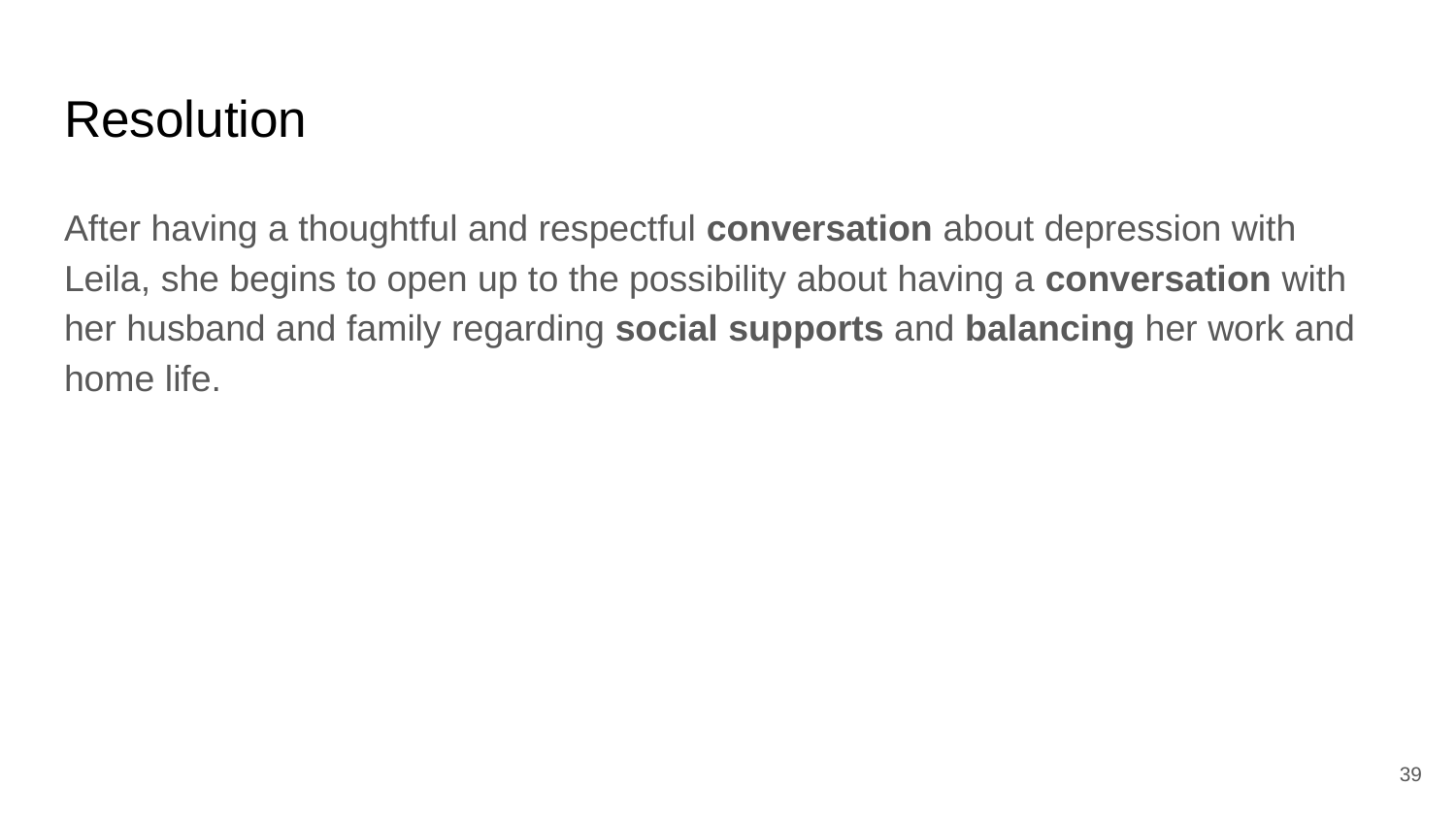

# Resolution
After having a thoughtful and respectful conversation about depression with Leila, she begins to open up to the possibility about having a conversation with her husband and family regarding social supports and balancing her work and home life.
‹#›

## Slide 40
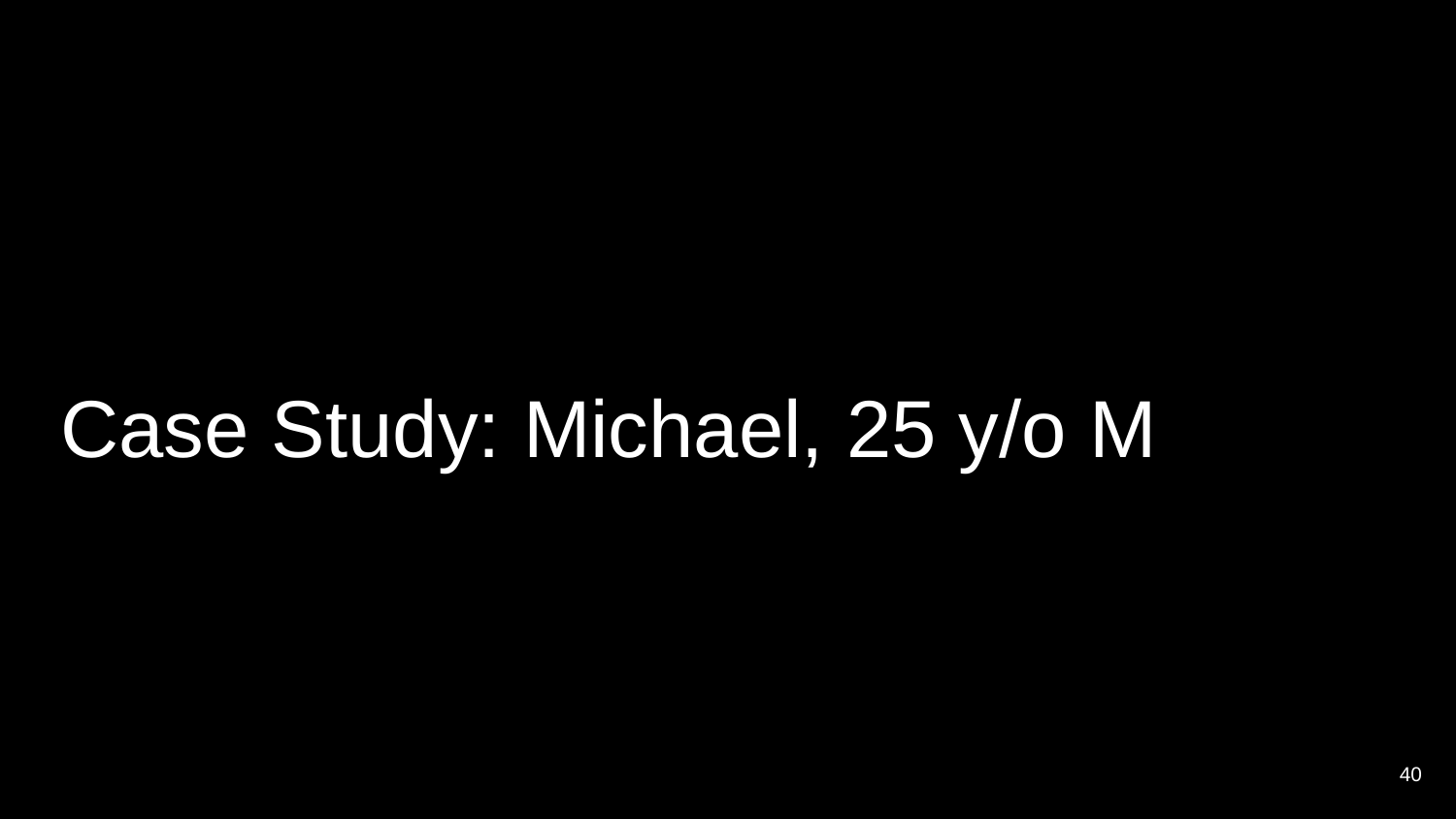

# Case Study: Michael, 25 y/o M
‹#›

## Slide 41
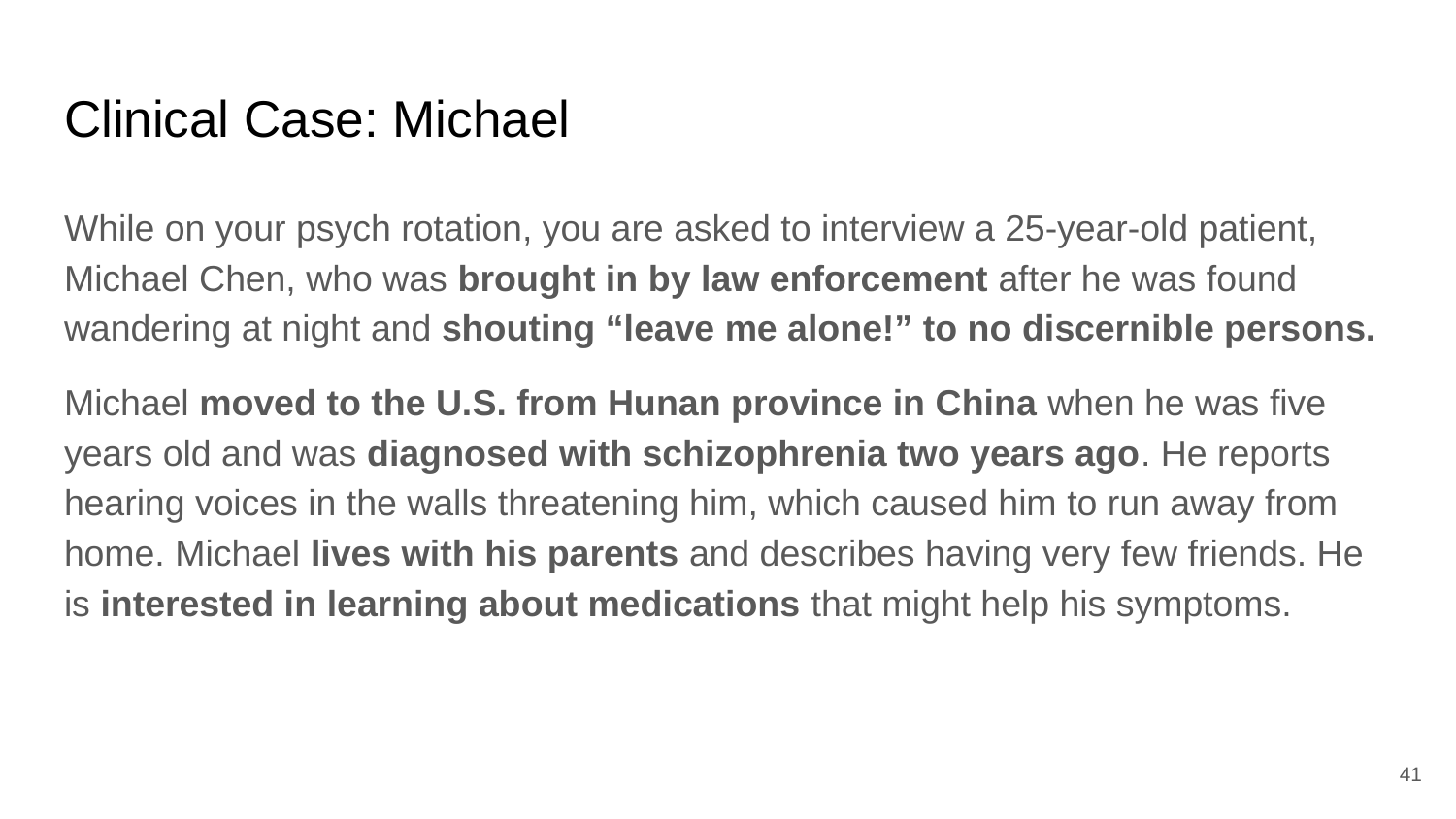

# Clinical Case: Michael
While on your psych rotation, you are asked to interview a 25-year-old patient, Michael Chen, who was brought in by law enforcement after he was found wandering at night and shouting “leave me alone!” to no discernible persons.
Michael moved to the U.S. from Hunan province in China when he was five years old and was diagnosed with schizophrenia two years ago. He reports hearing voices in the walls threatening him, which caused him to run away from home. Michael lives with his parents and describes having very few friends. He is interested in learning about medications that might help his symptoms.
‹#›

## Slide 42
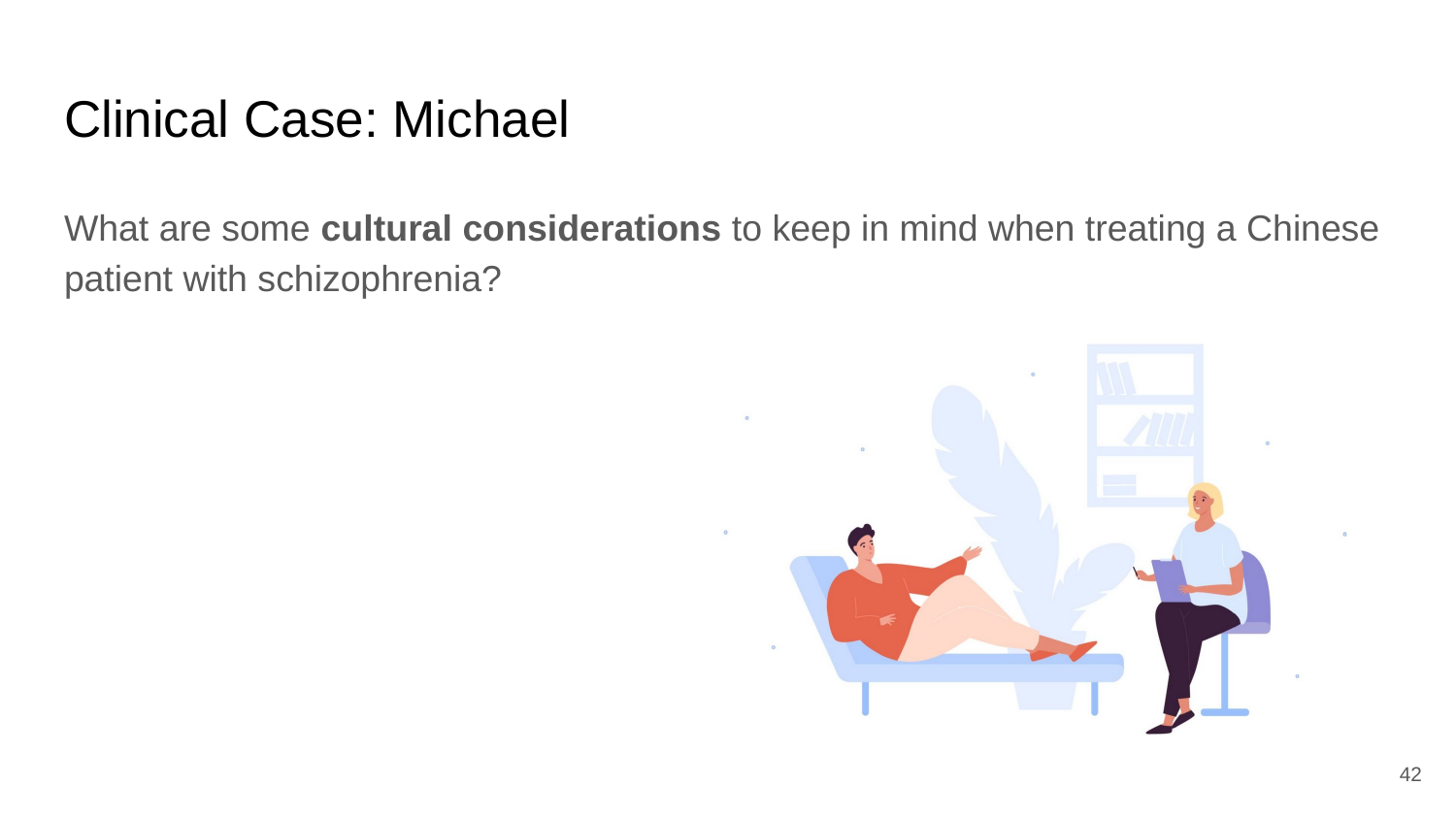

# Clinical Case: Michael
What are some cultural considerations to keep in mind when treating a Chinese patient with schizophrenia?
‹#›

## Slide 43
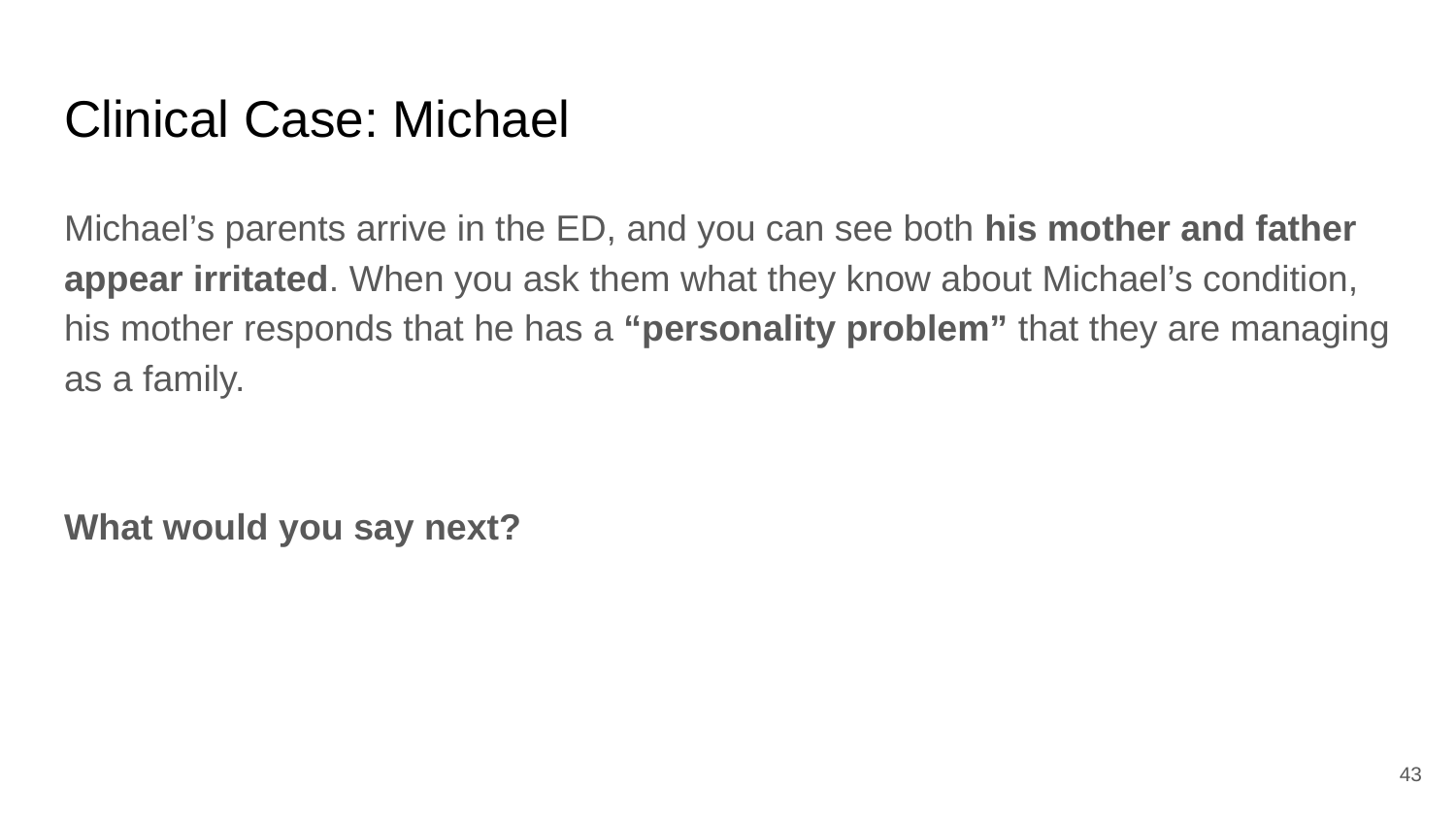

# Clinical Case: Michael
Michael’s parents arrive in the ED, and you can see both his mother and father appear irritated. When you ask them what they know about Michael’s condition, his mother responds that he has a “personality problem” that they are managing as a family.
What would you say next?
‹#›

## Slide 44
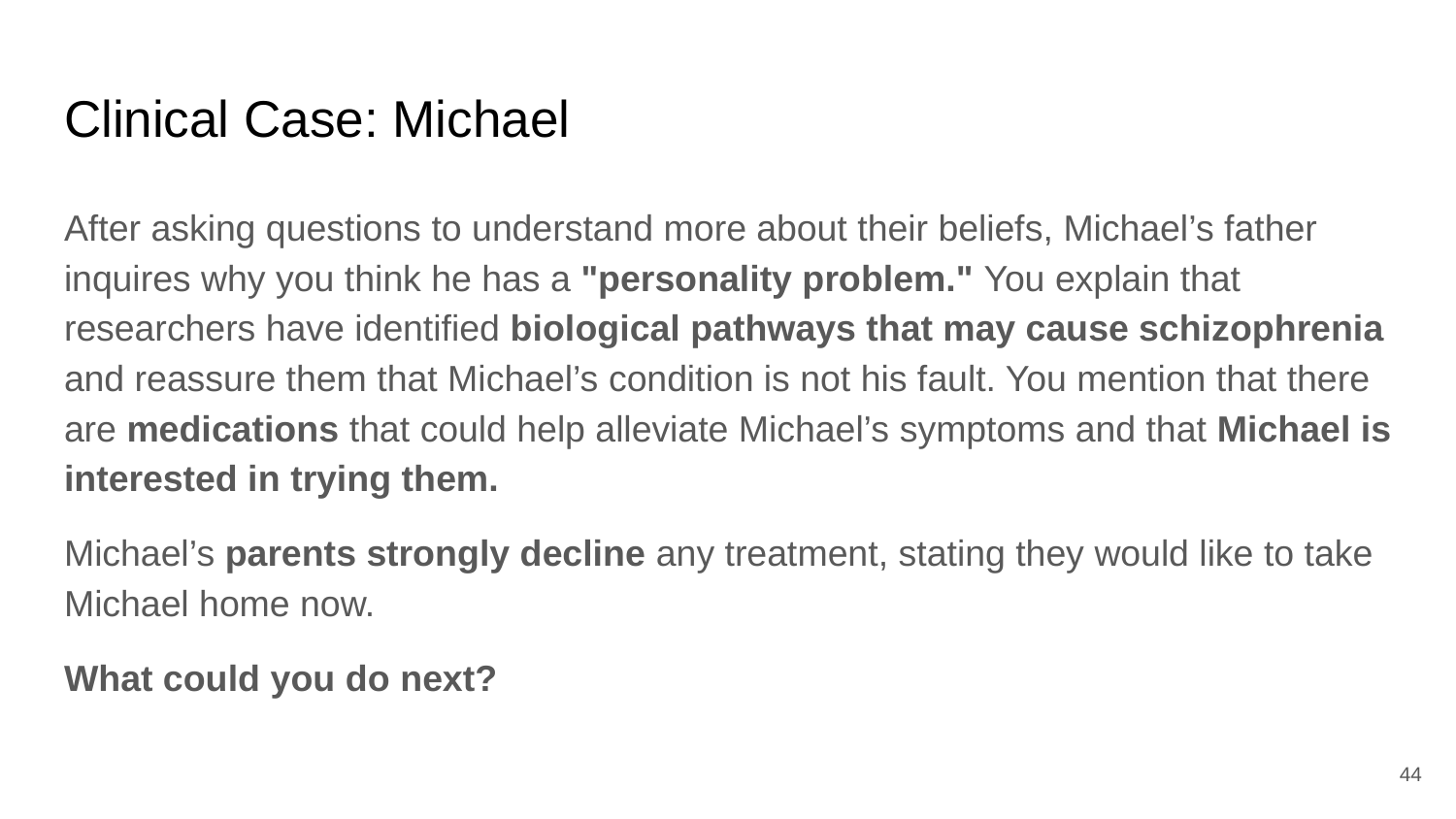

# Clinical Case: Michael
After asking questions to understand more about their beliefs, Michael’s father inquires why you think he has a "personality problem." You explain that researchers have identified biological pathways that may cause schizophrenia and reassure them that Michael’s condition is not his fault. You mention that there are medications that could help alleviate Michael’s symptoms and that Michael is interested in trying them.
Michael’s parents strongly decline any treatment, stating they would like to take Michael home now.
What could you do next?
‹#›

## Slide 45
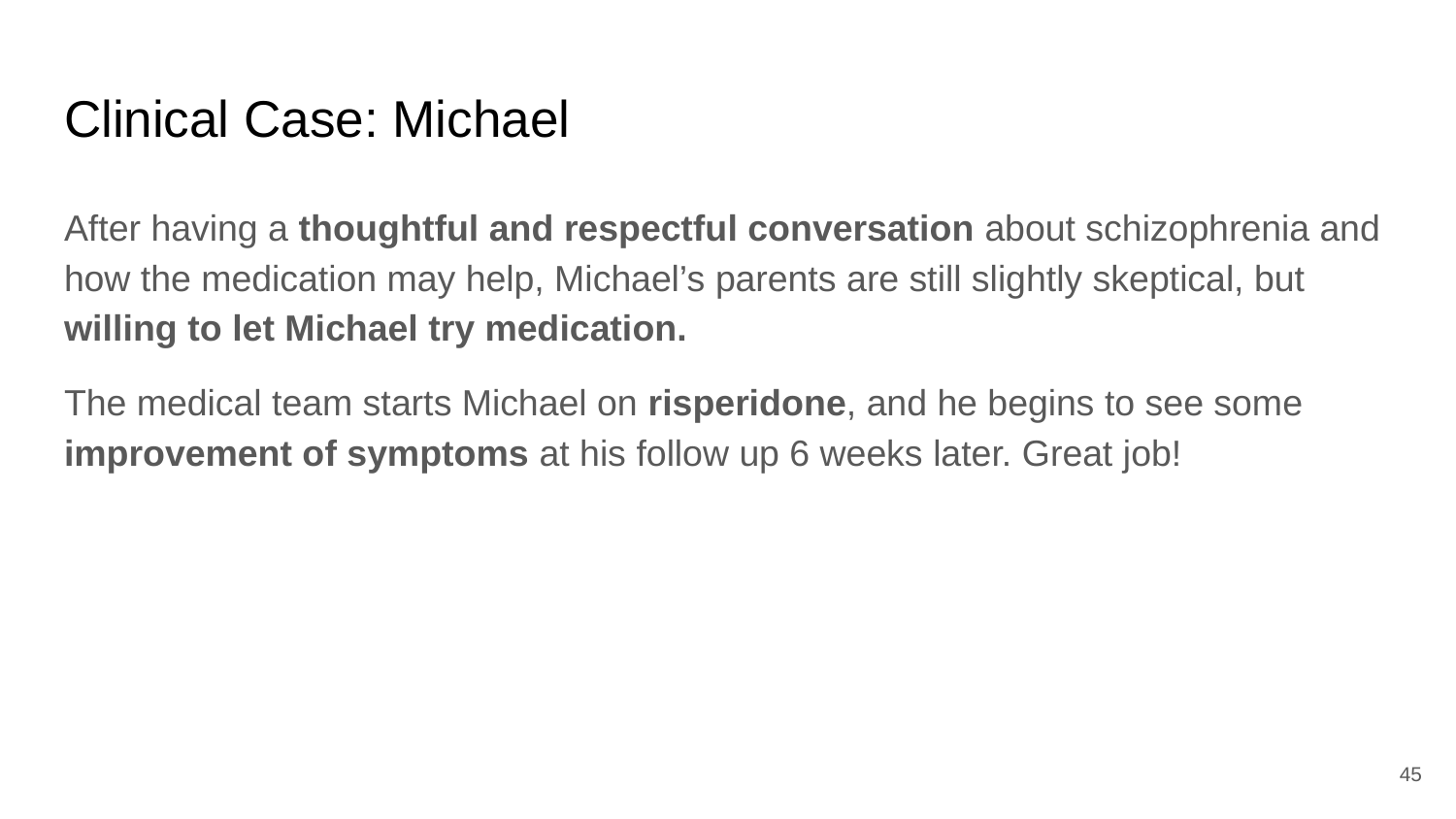

# Clinical Case: Michael
After having a thoughtful and respectful conversation about schizophrenia and how the medication may help, Michael’s parents are still slightly skeptical, but willing to let Michael try medication.
The medical team starts Michael on risperidone, and he begins to see some improvement of symptoms at his follow up 6 weeks later. Great job!
‹#›

## Slide 46
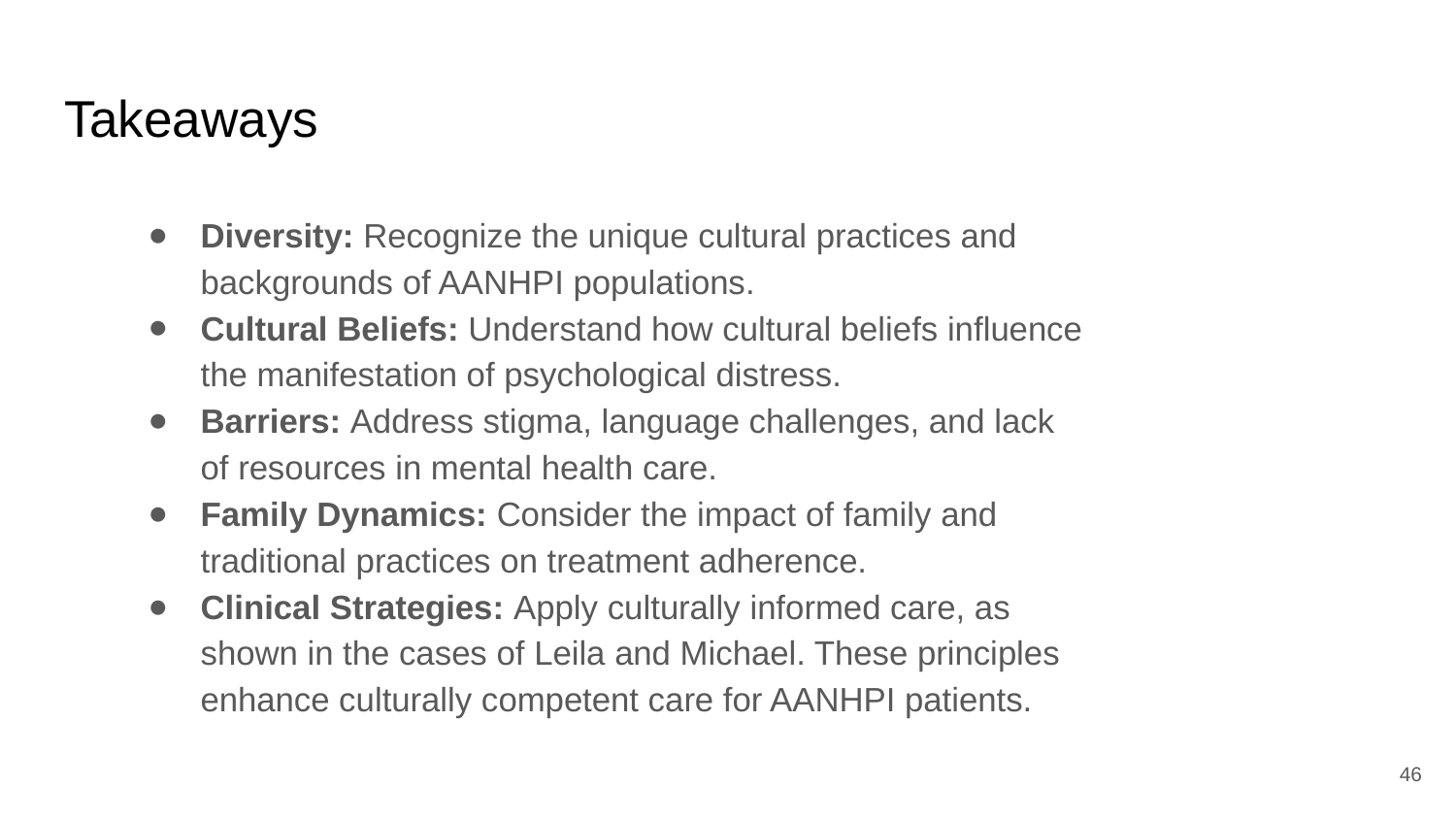

# Takeaways
Diversity: Recognize the unique cultural practices and backgrounds of AANHPI populations.
Cultural Beliefs: Understand how cultural beliefs influence the manifestation of psychological distress.
Barriers: Address stigma, language challenges, and lack of resources in mental health care.
Family Dynamics: Consider the impact of family and traditional practices on treatment adherence.
Clinical Strategies: Apply culturally informed care, as shown in the cases of Leila and Michael. These principles enhance culturally competent care for AANHPI patients.
‹#›

## Slide 47
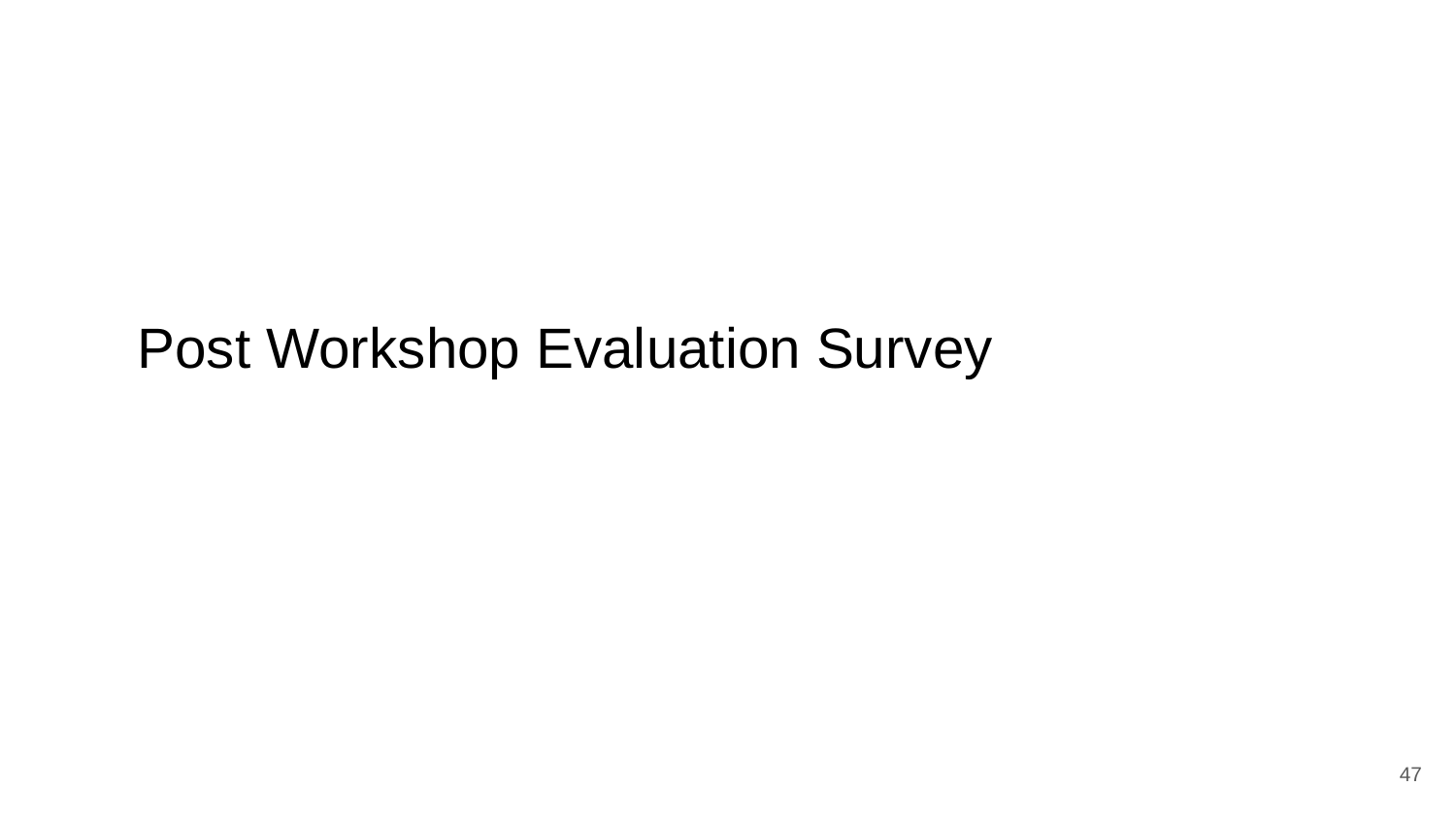

# Post Workshop Evaluation Survey
‹#›

## Slide 48
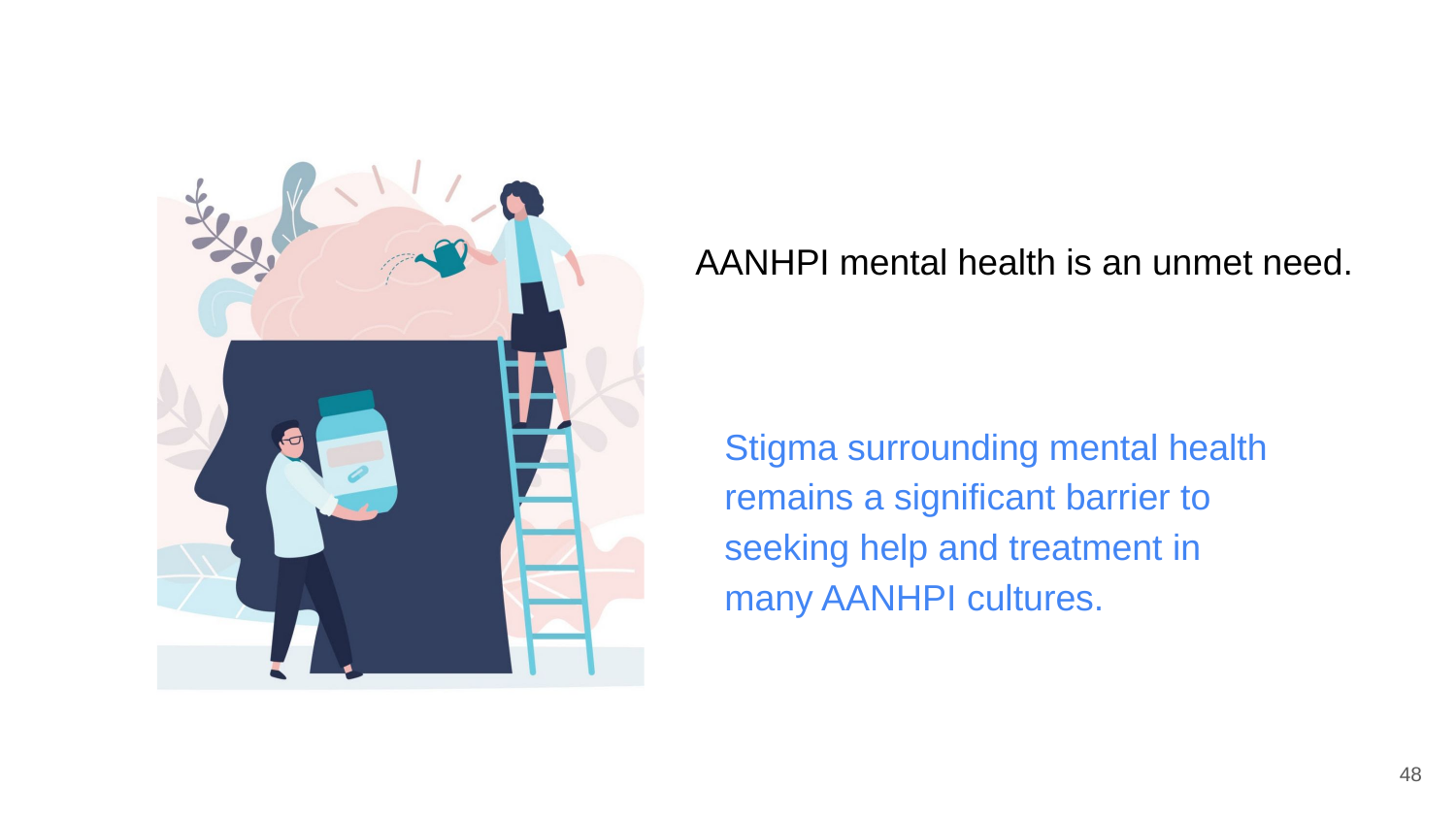

AANHPI mental health is an unmet need.
Stigma surrounding mental health remains a significant barrier to seeking help and treatment in many AANHPI cultures.
‹#›
