# Supplementary material for: Enhancing Cultural Humility: Addressing Mental Health Disparities in AANHPI Communities
Source: MedEdPORTAL. 2026 May 21;22:11599. doi: 10.15766/mep_2374-8265.11599 (PMC13192378; doi:10.15766/mep_2374-8265.11599)
Supplement: Supplementary file 1 — AANHPI Mental Health Workshop.pptxPre- and Postworkshop Survey.docxFacilitator Guide.docx [file mep_2374-8265.11599-s001.zip › B. Pre- and Postworkshop Survey.docx]

### Information Sheet for Participants

IRB #**AAAV4220**

**Enhancing Cultural Competence in Medical Students for AANHPI Mental Health Care**

**This information sheet should be included when using this survey.**

#### Purpose of the Study:

This study aims to evaluate the effectiveness of an educational intervention designed to improve medical students' knowledge, attitudes, and confidence in providing culturally competent care to Asian American, Native Hawaiian, and Pacific Islander (AANHPI) patients. This workshop is a part of APAMSA’s research initiative on advancing AANHPI health education for medical students.

#### Procedures:

- You will be asked to complete a pre-survey before the educational session and a post-survey afterward.
- The surveys will assess your knowledge, attitudes, and confidence in providing culturally competent care.

#### Voluntary Participation:

- Your participation in this study is entirely voluntary.
- You may choose not to participate or withdraw at any time without any consequences.
- You may participate in the workshop without filling out the surveys.
- Participation in research is entirely voluntary. Your decision whether or not to participate will have no impact on your employment, student status, or any other entitlements. If you do choose to participate, the answers given will have no impact on salary, grade, or employment with Columbia University Irving Medical Center (CUIMC) or New-York Presbyterian (NYP).

#### Risks:

- The study poses minimal risk, primarily involving the potential for mild discomfort when reflecting on your knowledge or attitudes.

#### Benefits:

- You may gain valuable insights and skills that enhance your ability to care for diverse patient populations.
- Your participation will contribute to improving medical education and patient care.

#### Confidentiality:

- Your survey responses will be anonymous.
- Data will be securely stored and only accessed by the research team.

#### Consent:

- By completing and submitting the surveys, you are giving your implied consent to participate in the study.

#### Contact Information:

If you have any questions or concerns about the study, please contact:

Karen Chen (kc3746@cumc.columbia.edu)

Thank you for considering participation in this important study!

**PRE - Workshop Survey**

**Please rate the following from 1-5 (strongly agree.)**

| **I am knowledgeable about mental health disparities in AANHPI patients.** |  |
| --- | --- |
| **I am able to discern how social and cultural factors may affect patient care in AANHPI populations.** |  |
| **I would feel confident conducting a psych evaluation for an AANHPI patient.** |  |
| **My medical school does a good job at teaching about mental health disparities in AANHPI populations.** |  |

Multiple Choice

1. AANHPI individuals are ________ to seek out mental health services compared to their White peers.
2. 2x more likely
3. 2x less likely
4. **5x less likely**
5. 7x more likely
6. In AANHPI communities, which of the following is/are driver(s) of mental health problems?
   1. Discrimination
   2. Historical trauma
   3. Model Minority stereotype
   4. Perpetual foreigner stereotype
   5. **All of the above**
7. What are the most common barriers to mental healthcare among AANHPI populations?
   1. Transportation issues, language barriers, and cost
   2. **Cost, lack of insurance, and not knowing their options**
   3. Lack of insurance, long waiting times, and language barriers
   4. Not knowing their options, distance to healthcare facilities, and high deductibles
8. True or False: Asian Americans report lower rates of anxiety as compared to White Americans, which is consistent with rates of anxiety in epidemiological studies. (T/F) **False**

**PRE - Workshop Survey**

Demographic data

1. In which state is your current academic medicine center (e.g. medical school, residency, etc.) located?

2. Are you a (choose one):

a. Medical Student

b. Intern/Resident

c. Fellow

d. Faculty

e. Staff

f. Other (please specify)

3. What is your race/ethnicity (choose all that apply):

a. White

b. Black or African American

c. Asian

d. Hispanic

e. American Indian or Alaska Native

f. Other

**POST - Workshop Survey**

**Please rate the following from 1-5 (strongly agree.)**

| **I am knowledgeable about mental health disparities in AANHPI patients.** |  |
| --- | --- |
| **I am able to discern how social and cultural factors may affect patient care in AANHPI populations.** |  |
| **I would feel confident conducting a psych evaluation for an AANHPI patient.** |  |
| **My medical school does a good job at teaching about mental health disparities in AANHPI populations.** |  |

1. AANHPI individuals are ________ to seek out mental health services compared to their White peers.
2. 2x more likely
3. 2x less likely
4. **5x less likely**
5. 7x more likely
6. In AANHPI communities, which of the following is/are driver(s) of mental health problems?
   1. Discrimination
   2. Historical trauma
   3. Model Minority stereotype
   4. Perpetual foreigner stereotype
   5. **All of the above**
7. What are the most common barriers to healthcare among AANHPI populations?
   1. Transportation issues, language barriers, and cost
   2. **Cost, lack of insurance, and not knowing their options**

Lack of insurance, long waiting times, and language barriers

- 1. Not knowing their options, distance to healthcare facilities, and high deductibles

1. True or False: Asian Americans report lower rates of anxiety as compared to White Americans, which is consistent with rates of anxiety in epidemiological studies. (T/F) **False**
2. To what extent do you agree that the workshop learning objectives were met?
3. Suggestions for improvement / Feedback
