# Supplementary material for: Enhancing Cultural Humility: Addressing Mental Health Disparities in AANHPI Communities
Source: MedEdPORTAL. 2026 May 21;22:11599. doi: 10.15766/mep_2374-8265.11599 (PMC13192378; doi:10.15766/mep_2374-8265.11599)
Supplement: Supplementary file 1 — AANHPI Mental Health Workshop.pptxPre- and Postworkshop Survey.docxFacilitator Guide.docx [file mep_2374-8265.11599-s001.zip › C. Faciltator Guide.docx]

***Enhancing Cultural Competence: Addressing Mental Health Disparities in AANHPI Communities***

***Facilitator Guide***

**Overall Goals**

The aim of this module is to enhance cultural competence among medical students, residents, and other health professionals in addressing mental health disparities in Asian American, Native Hawaiian, and Pacific Islander (AANHPI) communities. This module is designed to engage learners from diverse backgrounds and promote a deeper understanding of the unique mental health challenges faced by various AANHPI subpopulations.

**Workshop Objectives**

1. Explain the cultural influences and social determinants that impact mental health perceptions within AANHPI communities, using case studies for illustration.

2. Assess the mental health needs specific to different AANHPI subgroups through analysis of demographic data and health service utilization.

3. Develop and apply culturally informed communication strategies to improve mental health discussions and interventions with AANHPI patients.

**Workshop Handouts and Materials**

1. Computer setup with audio capability, and connection to projector.

2. Mobile device for participants.

**Suggested Agenda and Timeline**

- 3 min: Pre-workshop evaluation and Introduction (Slides 1-2)

- 5 min: AANHPI Mental Health Burdens (Slides 3-7)

- 7 min: Barriers to AANHPI Mental Health Care (Slides 8-11)

- 15 min: Mental Health Disorders in AANHPI Populations (Slides 12-16)

- 15 min: Considerations for Healthcare Providers (Slides 17-21)

- 10 min: Clinical Cases (Slides 22-30)

- 5 min: Post-workshop evaluation

Pre-reading Materials:
Facilitators are encouraged to review the following materials in advance to support thoughtful and inclusive dialogue around AANHPI mental health stigma. These resources offer guidance on recognizing implicit bias, unpacking stereotypes, and fostering psychologically safe spaces.

- **AAPI Data & UCLA Center for Health Policy Research.** *Piecing the Puzzle of AANHPI Mental Health* (2024). This report analyzes disaggregated data from California’s Health Interview Survey and community engagement efforts to uncover structural and cultural barriers to mental health care in AANHPI communities. It emphasizes the need for culturally and linguistically appropriate services and calls for improved data collection and policy reform. Read the full report
- **Stanford Medicine Children’s Health.** *Understanding Culture to Address Mental Health in the AANHPI Community* (2024). This article explores how cultural framing, acculturative stress, and stigma influence mental health experiences among AANHPI youth. It highlights the importance of cultural humility and the need for clinicians to understand family dynamics and culturally acceptable treatment pathways. View the article
- **National Partnership for Women & Families.** *Let’s Talk Mental Health in AANHPI Communities* (2024). A reflective piece that incorporates lived experiences and community perspectives on stigma, intergenerational trauma, and the “survivor mentality” that often deters help-seeking. It’s a valuable resource for facilitators seeking to understand emotional and cultural barriers to care. Explore the blog

**Note on Active Learning Integration:** Facilitators are encouraged to incorporate active learning strategies throughout the session. This may include:

- **Small Group Discussions:** Break participants into groups to explore cultural case scenarios, share personal reflections, or brainstorm strategies for culturally responsive care.
- **Case-Based Role-Plays:** Expand existing clinical vignettes (e.g., Leila and Michael) into interactive role-play exercises where learners practice communication techniques and navigate cultural dynamics in real time.
- **Structured Reflection Prompts:** Use guided questions at key intervals to prompt learners to reflect on their own biases, cultural assumptions, and clinical decision-making processes.

**Slide Instructions**

**Before starting please have everyone fill out the pre-workshop survey [CREATE Appendix B as an online survey]:**

- **Have it on the board prior to starting (as a QR code) - will be attached.**

Slide 1: Introduction

Facilitators should introduce themselves and explain the structure of the module. Mention that the presentation will cover cultural influences, specific mental health needs of different AANHPI subgroups, and strategies for effective communication in clinical settings. Emphasize the importance of cultural competence in addressing mental health disparities.

Narration: “Welcome everyone. We will be guiding you through today’s module on ‘Enhancing Cultural Competence: Addressing Mental Health Disparities in AANHPI Communities.’ This module is structured to provide an in-depth look into the unique mental health challenges faced by AANHPI communities. We will cover cultural influences, specific mental health needs of different AANHPI subgroups, and strategies for effective communication in clinical settings. Our goal is to enhance your ability to provide culturally competent care and address mental health disparities in these communities.”

Slide 2: Learning Objectives

Facilitators should read aloud the learning objectives and remind the audience that a post-module evaluation will be used to quantify the effectiveness of the learning module in addressing these objectives.

Narration: “By the end of this module, you will be able to explain the cultural influences and social determinants that impact mental health perceptions within AANHPI communities, using case studies for illustration. You will assess the mental health needs specific to different AANHPI subgroups through analysis of demographic data and health service utilization. Finally, you will develop and apply culturally informed communication strategies to improve mental health discussions and interventions with AANHPI patients. We will also conduct a post-module evaluation to measure the effectiveness of this session.”

Slide 3: What is AANHPI?

Facilitators should define AANHPI and present the key statistics related to the AANHPI population from the 2020 census.

Narration: “AANHPI stands for Asian American, Native Hawaiian, and Pacific Islander. According to the 2020 census, 20.6 million people identify as AANHPI, with 19.9 million identifying as Asian and 690,000 as Native Hawaiian or other Pacific Islander. The AANHPI community is incredibly diverse, with 21 detailed groups and 23 languages originating from Asia. Understanding this diversity is crucial for addressing the unique mental health needs of these populations.”

Slide 4: Reflection

Have student’s answer “What does mental health mean to you” in the PollEv.

Slide 5: Mental Health Overview

Facilitators should explain the definition of mental health according to the WHO and its importance beyond the absence of mental disorders.

Narration: “Mental health, as defined by the WHO, is a state of well-being that enables people to cope with the stresses of life, realize their abilities, learn well, work well, and contribute to their community. It is more than just the absence of mental disorders; it is a basic human right. However, medical schools and society have fallen short in acknowledging the needs of AANHPI patients, even as the number of AANHPI medical students continues to rise.”

Slide 6: Shortcomings in Medical Education

Facilitators should read the quote on the slide.

Narration: ““Medical schools, like many institutions, are need to evolve in their efforts to recognize and address the nuanced needs of AANHPI patients, even as the number of AANHPI medical students continues to grow.”

This sentence means that medical schools—similar to broader societal systems—have not adequately recognized or addressed the complex and specific factors that affect the mental health and healthcare experiences of Asian American, Native Hawaiian, and Pacific Islander (AANHPI) patients. These “nuanced needs” refer to things like:

Cultural beliefs about mental illness, Language barriers and limited access to culturally concordant providers, Stigma within AANHPI communities, Underrepresentation in mental health research and curricula

At the same time, the number of AANHPI individuals entering medical school is increasing, which makes the lack of attention to these issues even more striking.

## Slide 7: Shortcomings in Medical Education – AANHPI Mental Health

### Narration

“AANHPI populations are incredibly unrecognized in medical education, with only 0.01% of studies found on MEDLINE even mentioning them as a studied subgroup. Currently, there are no publications that focus on destigmatizing mental health in AANHPI communities and in the general population to our knowledge. This highlights the urgent need for more research and resources dedicated to these populations.”

### Facilitator Talking Points

- Underserved in Research and Curriculum The extreme underrepresentation of AANHPI populations in medical literature reflects broader gaps in clinical training, public health policy, and culturally responsive care.
- Beyond Medline: Expanding the Evidence Base Encourage learners to explore interdisciplinary and community-based sources that offer richer insights into AANHPI mental health.

### Key Literature Contributions

| Source | Contribution to Education Reform |
| --- | --- |
| AAPI Data & UCLA CHPR (2024) Piecing the Puzzle of AANHPI Mental Health | Provides disaggregated data and community-informed analysis, revealing subgroup-specific disparities and stigma patterns often missed in aggregated studies. |
| Psychological Services Special Issue (2023) Improving Access and Quality of Mental Health Care | Offers practice-based evidence and culturally tailored interventions, highlighting the need for clinician training models that reflect AANHPI realities. |
| Asian American Psychological Association (AAPA) AAPA Publications | Publishes frameworks rooted in AANHPI psychology, including identity development, acculturation stress, and destigmatization strategies. |

### Facilitator Tip

Ask learners:

- *What assumptions might be embedded in the current curriculum?*
- *How can we advocate for more inclusive research and training models?*
- *What would a culturally responsive mental health curriculum for AANHPI patients look like?*

Slide 8: Part 1: AANHPI Mental Health Burdens

Facilitators should introduce the first section of the presentation, which focuses on the mental health burdens within AANHPI communities.

Narration: “In this section, we will explore the mental health burdens faced by AANHPI communities. We will discuss statistical data, the impact of the COVID-19 pandemic, and the unique challenges faced by different subgroups within the AANHPI population.”

Slide 9: Question

Have participants answer the Question in PollEV.

Slide 10: AANHPI Mental Health Statistics

Facilitators should present the key statistics related to mental health in AANHPI populations, emphasizing the lower rates of mental health service utilization compared to their White peers.

Narration: “Despite the significant mental health needs within AANHPI communities, individuals in these groups are up to 5 times less likely to seek mental health services compared to their White peers. Up to 18.1% of AANHPI individuals experience a psychiatric disorder in their lifetime. The COVID-19 pandemic has further increased rates of discrimination and mental health challenges, with 35% of AANHPI individuals reporting symptoms of anxiety disorder during this period.”

Slide 11: Impact of COVID-19

Facilitators should discuss the increased rates of discrimination and mental health challenges faced by AANHPI individuals during the COVID-19 pandemic.

Narration: “The COVID-19 pandemic has exacerbated mental health challenges within AANHPI communities. With increased rates of discrimination and hate directed towards AANHPI individuals, addressing these mental health challenges is more pressing than ever before. Studies show that 35% of AANHPI individuals reported symptoms of anxiety disorder during the pandemic.”

Slide 12: Have students submit reflections in the PollEV on “What are some potential causes of mental health problems in AANHPI populations?”

Slide 13: Drivers of Mental Health Problems

Facilitators should explain how discrimination, historical trauma, and stereotypes such as the Model Minority Myth contribute to mental health problems. Use examples and case studies to illustrate these points.

Narration: “Several factors drive mental health problems within AANHPI communities. Discrimination, especially the anti-Asian harassment following COVID-19, has increased the prevalence and severity of mental health issues. Historical trauma, such as U.S. colonization, resettlement, and racist policies like the Japanese American internment and the Chinese Exclusion Act, also play a significant role. The Model Minority Stereotype and the Perpetual Foreigner Stereotype further exacerbate these issues by creating unrealistic expectations and barriers to belonging.”

Slide 14: Differing Burden Across Subgroups

Facilitators should explain the differences in mental health burdens across various AANHPI subgroups, providing specific statistics and examples.

Narration: “Mental health burdens vary significantly across AANHPI subgroups. For example, 33% of Korean American adults experience symptoms of depression, compared to less than 16% of Chinese Americans. Similarly, 78% of Filipino American women rate their mental health as excellent or very good, compared to 45% of Chinese American women and 50% of Vietnamese American women. Japanese American and Korean American men are at greater risk of suicide than other Asian American men. Understanding these differences is crucial for effective mental health care.”

Slide 15: Differing Presentations Across Subgroups

Facilitators should discuss how mental health symptoms present differently across various AANHPI subgroups and the importance of culturally sensitive approaches.

Narration: “Mental health symptoms also present differently across AANHPI subgroups. South Asians often describe psychological distress through physical symptoms like lack of sleep, body aches, and digestive problems. Korean Americans may experience hwa-byung, or ‘anger syndrome,’ characterized by insomnia, fatigue, and panic. Native Hawaiians prefer traditional healing practices and a holistic approach to health, integrating physical, spiritual, emotional, and mental aspects. These variations highlight the need for culturally sensitive approaches in mental health care.”

Slide 16: Subgroup Specific Perceptions

Facilitators should present the differing perceptions of mental health conditions among various AANHPI subgroups.

Narration: “Perceptions of mental health conditions vary widely among AANHPI subgroups. For instance, there are common misconceptions among Korean Americans that medication treatments are addictive. Studies highlight the importance of involving family and community in understanding and treating mental health conditions. Chinese American adults often attribute mental health disorders to cultural and personality differences, while Vietnamese Americans may have different cultural contexts for understanding mental illness. It’s essential to recognize these subgroup-specific perceptions in clinical practice.”

Slide 17: Part 2: Barriers to AANHPI Mental Health Care

Facilitators should introduce the second section of the presentation, focusing on the barriers to accessing mental health care for AANHPI individuals.

Narration: “In this section, we will explore the barriers that AANHPI individuals face in accessing mental health care. We will discuss issues such as lack of access, social stigma, and language barriers, and examine how these factors impact mental health outcomes in these communities.”

Slide 18: Have students submit reflections to “What are some potential barriers to mental healthcare in AANHPI populations?” in PollEV.

Slide 19: Lack of Access to Mental Health Care

Facilitators should discuss the statistics on the lack of access to mental health care for AANHPI individuals and the common barriers they face.

Narration: “A 2021 survey by the National Alliance on Mental Illness found that 55% of Asian Americans needed mental health support but did not receive it. Analysis of data also showed that a significant percentage of Native Hawaiians, Pacific Islanders, and Asian Americans had difficulties accessing mental health services. Common barriers include cost, lack of insurance, and not knowing their options. Vulnerable groups include those with limited English proficiency, first-generation immigrants, and older generations.”

Slide 20: Lack of Access to Mental Health Care

Facilitators should explore the structural, financial, and informational barriers limiting access to mental health care for AANHPI communities.

Narration:

“Multiple barriers restrict mental health care access within AANHPI communities. Financial costs, such as those faced by 33% of Asian Americans diagnosed with depression and 40% for general medical care, present significant obstacles. Insurance coverage disparities are evident, with 6.8% of AANHPIs uninsured, and rates vary widely among subgroups, such as Japanese Americans (2.3%) versus Native Hawaiians and Pacific Islanders (12.3%). Additionally, many individuals are unaware of available mental health resources, with 13% of AANHPIs and up to 26% of Southeast Asians lacking awareness. Compounding these issues, vulnerable groups—such as those with limited English proficiency, first-generation immigrants, and older generations—face heightened challenges.

Slide 21: Barriers to Seeking Mental Health Care

Facilitators should discuss the impact of social and self-stigma on mental health care seeking behavior within AANHPI communities.

Narration: “Social and self-stigma surrounding mental health are significant barriers within AANHPI communities. Mental health is often considered a taboo topic, compounded by stereotypes like the Model Minority Myth and survivor mentality in Filipino culture. Language barriers and a lack of culturally specific awareness campaigns further hinder access to mental health care. Increasing cultural competence among providers and addressing stigma are essential steps in overcoming these barriers.”

Slide 22: Social Determinants of Health

Facilitators should review the key points from this slide, emphasizing how these social determinants affect health outcomes within Asian American communities.

**Narration:** "This slide provides an overview of the social determinants of health that impact Asian American communities. We discuss the significance of economic stability, noting the disparities in median income between Burmese and Indian Americans. In the realm of neighborhood and built environment, we highlight the home ownership rates among Asian Americans compared to the overall U.S. population. The section on health care access draws attention to challenges such as lack of insurance and a shortage of culturally competent providers. In terms of education, we examine the variance in educational attainment between Taiwanese and Bhutanese Americans. Lastly, we consider the social and community context, emphasizing the role of multigenerational households in providing support and potentially contributing to stress. Each of these factors plays a critical role in shaping the health outcomes of Asian American communities."

Slide 23: Part 3: Mental Health Disorders in AANHPI Populations

Facilitators should introduce the third section of the presentation, which focuses on the prevalence of mental health disorders within AANHPI populations.

Narration: “In this section, we will discuss the prevalence of mental health disorders within AANHPI populations. We will examine data on depression, suicide, anxiety, and schizophrenia, and explore the unique challenges faced by these communities in diagnosing and treating these conditions.”

Slide 24: Depression

Facilitators should discuss the prevalence of depression within AANHPI communities and the systemic barriers that prevent care.

Narration:

"Depression is a significant concern within AANHPI communities. Prevalence rates vary, with Southeast Asians reporting the highest rates at 19%, followed by South Asians at 11%, and East Asians at 9%. Native Hawaiians and Pacific Islanders (NHPI) face an alarming prevalence of 36.1%, the highest of any U.S. racial group. Despite this, barriers to care persist, including stigma, language difficulties, and financial constraints. For example, 1 in 3 Asian Americans diagnosed with depression are unable to seek care due to costs. Addressing these challenges requires reducing stigma and enhancing accessibility to culturally competent care."

Slide 25: Suicide

Facilitators should explore the prevalence and cultural factors influencing suicide in AANHPI youth.

Narration:

"Suicide is the leading cause of death for AANHPI youth aged 15–24, with rates doubling between 1999 and 2021. The leading causes of death in this age group include unintentional injury, homicide, and suicide. Cultural factors such as discrimination, familial pressures to succeed, cultural shame, and mental health stigma compound this issue. These factors create significant barriers to seeking help, underscoring the need for culturally sensitive suicide prevention and mental health support initiatives."

Slide 26: Anxiety Question

Facilitators should ask the question on the screen:

True or False: Asian Americans report lower rates of anxiety as compared to White Americans, which is consistent with rates of anxiety in epidemiological studies.

The answer is: False

Slide 27: Anxiety

Facilitators should engage the audience in discussing the misperceptions around anxiety in AANHPI communities.

Narration:

"True or False: Asian Americans report lower rates of anxiety compared to White Americans, as reflected in epidemiological studies. While this statement is true in reported statistics, it masks the reality that many cases go undiagnosed due to cultural stigma, underreporting, and barriers to accessing care. Recognizing and addressing these hidden challenges is crucial in providing appropriate support and interventions for AANHPI individuals struggling with anxiety."

Slide 28: Schizophrenia

Facilitators should examine the prevalence and challenges related to schizophrenia in AANHPI populations.

Narration:

"The lifetime prevalence of psychotic symptoms in Asian Americans is around 9.6%, with significant subgroup variations. For instance, Filipinos and 'Other Asians' are more likely to experience psychotic symptoms than Chinese, while Vietnamese have the lowest rates. However, disparities exist in treatment, with Asian Americans experiencing a longer duration of untreated psychosis compared to other ethnic groups. Research into schizophrenia within AANHPI populations remains limited due to small sample sizes and a lack of subpopulation-specific data, emphasizing the need for more inclusive and representative studies."

Slide 29: Considerations for Healthcare Providers

Facilitators should introduce the fourth section of the presentation, focusing on considerations for healthcare providers in addressing mental health disparities in AANHPI communities.

Narration: “In this section, we will discuss important considerations for healthcare providers in addressing mental health disparities in AANHPI communities. We will cover topics such as understanding cultural beliefs, traditional practices, and the impact of historical and current racism and oppression on AANHPI patients.”

Slide 30: APA Recommendations

Facilitators should present the American Psychological Association's recommendations for providing culturally competent care to AANHPI patients.

Narration: “The American Psychological Association recommends that healthcare providers be aware of historical stereotypes and myths, assess their own biases, and understand how historical and current racism and oppression affect AANHPI patients. It is also important to be knowledgeable and respectful of traditional practices and cultural values, as well as Asian-American/Pacific Islander indigenous healing practices.”

Slide 31: Cultural Beliefs and Distress

Facilitators should explain the cultural beliefs related to psychological distress in various AANHPI subgroups and how these beliefs can vary and can influence the manifestation of symptoms and treatment approaches. Facilitators should emphasize the diversity of experiences within AANHPI communities and the importance of avoiding assumptions about cultural values and practices.

Narration: “Cultural beliefs can significantly influence how psychological distress is perceived and expressed. For example, in Vietnamese culture, mental health issues may be seen as a consequence of past misdeeds or ancestral sins. Somatic symptoms, such as body aches and insomnia, are common presentations of psychological distress in many AANHPI cultures. Additionally, the experiences of AANHPI patients are immensely diverse, and healthcare providers should avoid making assumptions about a patient’s cultural values and practices. It’s important to consider the implications of treatments on the patient’s family members, as familial stigma can affect adherence to treatment.”

Slide 32: Part 5: Clinical Cases

Facilitators should introduce the fifth section of the presentation, which includes clinical cases to apply the concepts covered in the module.

Narration: “Now, let’s apply what we’ve learned through clinical cases. These cases will help us understand the practical implications of cultural competence in mental health care for AANHPI patients. We will discuss scenarios involving patients from different AANHPI backgrounds and explore appropriate communication strategies, cultural considerations, and treatment plans. Let’s start with our first case.”

Slide 33: Clinical Case - Leila

Facilitators should introduce the first case.

Narration: “For the first case we have, Leila, 27y/o F. Let’s begin”

Slide 34: Clinical Case - Leila

Facilitators should present the case of Leila Singh, a 56-year-old woman with symptoms of psychological distress, and discuss the cultural considerations in her treatment.

Narration: “While you are on your Obstetrics & Gynecology rotation, you are assigned to a patient named Leila Singh. She is a 56-year-old woman who is presenting for a check-up and describes ‘feeling off’ recently.”

### **Slide 35: Cultural Considerations for Leila**

**Narration:** “What are some cultural considerations to keep in mind when interviewing Leila? Consider family dynamics, traditional medicine, spirituality, work/home duties, and stigma.”

**Facilitator Insights:**

- **Themes to Draw Out:**
  - Family-centered decision-making and emotional restraint in collectivist cultures.
  - The role of traditional medicine and spiritual practices in coping.
  - Gendered expectations around caregiving and emotional labor.
  - Cultural stigma surrounding mental illness and psychiatric labels.
- **How to Probe Deeper:**
  - Ask learners: “How might Leila’s cultural background shape her understanding of emotional distress?”
  - Encourage reflection on how cultural norms may influence symptom presentation (e.g., somatic complaints vs. emotional language).
- **Navigating Difficult Responses:**
  - If learners stereotype or generalize, gently redirect: “Let’s think about how these dynamics might vary within South Asian communities.”
  - If someone dismisses cultural relevance, ask: “What might we miss if we don’t explore these layers?”

### **Slide 36: Detailed Case Information – Leila**

**Narration:** “Leila reports feeling increasingly tired and lacking energy for the past year. She describes persistent headaches, body aches, and digestive issues such as loss of appetite and occasional stomach discomfort. She also mentions feeling ‘down’ and less interested in social activities or hobbies she once enjoyed. Leila initially attributed these symptoms to work stress and to her ‘menopause’ but is now concerned as they have persisted for over a year despite trying to rest and manage her workload better.”

**Facilitator Insights:**

- **Themes to Draw Out:**
  - Somatization as a culturally normative expression of psychological distress.
  - The intersection of physical and emotional symptoms in clinical interviews.
  - The impact of gender roles and life transitions (e.g., menopause) on mental health.
- **How to Probe Deeper:**
  - Ask: “What might be the significance of Leila attributing her symptoms to menopause or work stress?”
  - Explore: “How do we differentiate between culturally normative coping and clinical depression?”
- **Navigating Difficult Responses:**
  - If learners focus only on physical symptoms, prompt: “What else might be going on beneath the surface?”
  - If someone questions the relevance of cultural framing, ask: “How might Leila’s interpretation of her symptoms affect her willingness to seek care?”

### **Slide 37: Next Steps in the Interview**

**Narration:** “What would you want to get a better sense of next? It's important to gather a thorough social history. What are some SDH that we should consider as well?”

**Facilitator Insights:**

- **Themes to Draw Out:**
  - Social determinants of health (SDH): caregiving burden, employment stress, intergenerational expectations, access to culturally concordant care.
  - Emotional isolation despite living in a multigenerational household.
  - Religious and spiritual coping mechanisms.
- **How to Probe Deeper:**
  - Ask: “What questions would help you understand Leila’s support system?”
  - Explore: “How might her role in the household affect her mental health?”
- **Navigating Difficult Responses:**
  - If learners overlook SDH, prompt: “What structural or systemic factors might be contributing to Leila’s distress?”
  - If someone assumes family support is protective, ask: “What if family involvement also adds pressure?”

### **Slide 38: Social History – Leila**

**Narration:** “Leila currently lives with her husband and her elderly parents, who are very involved in her life. She has not brought up her feelings to them because she thinks they ‘would not be happy.’ As a practicing Hindu, Leila engages in daily prayers and meditation, which are integral to her routine and coping mechanisms. Leila balances a demanding job with household responsibilities, feeling pressure to perform well in both areas. When you mention depression, Leila becomes nervous and apprehensive. What would you say next?”

**Facilitator Insights:**

- **Themes to Draw Out:**
  - Cultural stigma and fear of burdening family.
  - Spirituality as both a source of resilience and a potential barrier to seeking clinical care.
  - The tension between professional identity and traditional gender roles.
- **How to Probe Deeper:**
  - Ask: “How might Leila’s spiritual practices inform your approach to care?”
  - Explore: “What language might feel safer than ‘depression’ in this context?”
- **Navigating Difficult Responses:**
  - If learners suggest pushing a diagnosis, redirect: “How can we validate her experience without labeling?”
  - If someone assumes spiritual coping is sufficient, ask: “What if her practices are no longer enough?”

### **Slide 39: Resolution – Leila**

**Narration:** “After discussing her symptoms and the possibility of depression, Leila begins to consider talking to her husband and family about seeking social support and balancing her work and home life better. This case highlights the importance of culturally sensitive communication and involving family members in the treatment process.”

**Facilitator Insights:**

- **Themes to Draw Out:**
  - The power of gentle, respectful dialogue in reducing stigma.
  - Family involvement as a culturally aligned strategy for support.
  - The role of cultural humility in building trust and therapeutic alliance.
- **How to Probe Deeper:**
  - Ask: “What communication strategies helped Leila feel safe enough to open up?”
  - Explore: “How can we support patients in navigating family conversations about mental health?”
- **Navigating Difficult Responses:**
  - If learners express frustration with slow progress, reframe: “Why might pacing be important in culturally responsive care?”
  - If someone questions family involvement, ask: “How can we balance patient autonomy with cultural values?”.

Facilitator Talking Points:

- Emphasize how culturally responsive communication helped Leila feel safe enough to consider opening up to her family.
- Note that in many AANHPI communities, family plays a central role in health decisions, and involving them can reduce stigma and improve outcomes.
- Highlight the clinician’s role in gently exploring cultural values, validating emotional distress, and offering support without judgment.
- Reinforce that culturally sensitive care includes understanding gender roles, expectations around emotional expression, and the impact of migration or acculturation stress.

Anticipated Participant Responses:

- Learners may reflect on how they would approach similar conversations with patients who are hesitant to disclose emotional struggles.
- Some may share concerns about navigating family dynamics or cultural taboos around mental illness.
- Others may suggest strategies like using metaphors, normalizing distress, or framing treatment in terms of restoring balance or harmony.

Follow-Up Questions:

- What communication strategies helped Leila feel comfortable discussing her symptoms?
- How might cultural humility shape the clinician’s approach in this case?
- What are some ways to involve family members without breaching confidentiality or pressuring the patient?
- How would you adapt this approach for a patient from a different AANHPI subgroup or generational background?

Slide 40: Clinical Case - Michael

Facilitators should introduce the the second case.

Narration: “Great, now let’s move on to the second case. For the second case, we have, Michael, 25y/o M. Let’s begin.”

Slide 41: Clinical Case - Michael

Facilitators should present the case of Michael Chen, a 25-year-old man with symptoms of schizophrenia, and discuss the cultural considerations in his treatment.

Narration: “While on your psych rotation, you are asked to interview a 25-year-old patient, Michael Chen, who was brought in by law enforcement after he was found wandering at night and shouting ‘leave me alone!’ to no discernible persons. You learn that Michael moved to the U.S. from the Hunan province in China when he was five years old and was diagnosed with schizophrenia two years ago. Today, he described hearing voices in the walls saying they’re going to kill him, which is why he ran away from home, where he lives with his mother and father. Michael describes having very few friends because his parents don’t like him leaving the house. When you mention there are medications that may help his symptoms, he is very interested.”

Slide 42: Cultural Considerations for Michael

Facilitators should highlight the key cultural considerations to keep in mind when treating a Chinese patient with schizophrenia.

Narration: “What are some cultural considerations to keep in mind when treating a Chinese patient with schizophrenia?”

Facilitator Insights:

- Themes to Draw Out:
  - Stigma and shame: Mental illness may be viewed as a moral failing or family disgrace, leading to denial or concealment.
  - Somatization: Psychological distress may be expressed through physical symptoms, making diagnosis more complex.
  - Filial piety and family authority: Parents may play a central role in decision-making, especially in young adult patients.
  - Beliefs about causation: Mental illness may be attributed to spiritual imbalance, karma, or interpersonal conflict rather than biology.
  - Help-seeking behavior: Preference for traditional medicine or avoidance of psychiatric care due to fear of labeling.
- How to Probe Deeper:
  - Ask: “How might cultural beliefs shape the way schizophrenia is understood or described?”
  - Explore: “What role does family play in treatment decisions, and how can we respectfully engage them?”
- Navigating Difficult Responses:
  - If learners stereotype or generalize, redirect: “Let’s think about how these beliefs might vary across generations or regions.”
  - If someone dismisses cultural relevance, ask: “What might we miss if we don’t explore these perspectives?”

### Slide 43: Interaction with Michael’s Parents

Narration: “Michael’s parents arrive in the ED, and you can see both his mother and father are agitated. When you ask them what they know about Michael’s condition, his mother says he has a ‘personality problem.’ What would you say next?”

Facilitator Insights:

- Themes to Draw Out:
  - The phrase “personality problem” may reflect cultural framing or avoidance of psychiatric terminology.
  - Parents may be fearful of stigma, institutionalization, or long-term dependency.
  - Building trust requires validating concerns while gently introducing clinical language.
- How to Probe Deeper:
  - Ask: “What might ‘personality problem’ mean in this context?”
  - Explore: “How can we respond in a way that’s both clinically accurate and culturally sensitive?”
- Navigating Difficult Responses:
  - If learners suggest correcting the parents abruptly, redirect: “How might that affect rapport?”
  - If someone expresses frustration, reframe: “What’s the opportunity here to build understanding?”

### Slide 44: Further Discussion with Michael’s Parents

Narration: “After asking questions to learn more about the cultural context of their beliefs, Michael’s father asks why you think he has a ‘personality problem.’ You describe how researchers have identified some biological pathways that may cause schizophrenia and affirm that Michael’s condition is not his fault. You share that there are medications that may help Michael’s symptoms and that Michael is very interested in trying medication. Michael’s parents strongly decline any treatment, stating they would like to take Michael home now. What could you do next?”

Facilitator Insights:

- Themes to Draw Out:
  - The importance of shared decision-making and respecting family dynamics while advocating for the patient’s autonomy.
  - Using cultural humility to understand resistance and explore alternatives.
  - Balancing clinical urgency with relational trust-building.
- How to Probe Deeper:
  - Ask: “What strategies could help bridge the gap between Michael’s wishes and his parents’ concerns?”
  - Explore: “How might you reframe treatment in terms of restoring balance or supporting Michael’s strengths?”
- Navigating Difficult Responses:
  - If learners suggest overriding the parents, prompt: “What are the ethical and relational implications?”
  - If someone feels stuck, offer: “What community resources or cultural brokers might help mediate this conversation?”

Slide 45: Resolution – Michael

Narration: “After having a thoughtful and respectful conversation about schizophrenia and what the medication does, Michael’s parents are still slightly skeptical, but willing to let Michael try medication. The medical team starts Michael on risperidone, and he begins to see some improvement in symptoms at his next checkup. Great job!”

Facilitator Talking Points:

- Reinforce the value of respectful, culturally grounded dialogue in building trust.
- Note that partial skepticism is common and doesn’t preclude progress.
- Emphasize the importance of follow-up and continued engagement with both patient and family.

Anticipated Participant Responses:

- Learners may feel encouraged by the outcome and ask about next steps.
- Some may raise questions about medication choice or side effect management.
- Others may reflect on how they would handle similar cases in clinical practice.

Follow-Up Questions:

- What made this conversation effective despite initial resistance?
- How can clinicians maintain trust and engagement over time?
- What role does cultural humility play in navigating ongoing care?

Slide 46: Takeaways

Narration: “We’ve covered a lot of ground today, discussing the mental health disparities in AANHPI communities, the barriers to care, and strategies for providing culturally competent care. We have outlined some of the main points, such as recognizing the unique cultural practices and backgrounds of AANHPI population, understanding how cultural beliefs influence the manifestation of psychological distress, and how we may address stigma, language challenges, and lack of resources in mental health care. We’ve also looked at clinical cases to apply these concepts in real-world scenarios. Now, let’s open the floor for any questions you might have. Thank you for your attention and participation.”

Facilitator Talking Points:

- Summarize key themes: disparities, stigma, cultural beliefs, communication strategies.
- Reinforce the shift from “cultural competence” to “cultural humility” and “culturally responsive care.”
- Encourage learners to reflect on how they’ll apply these insights in future clinical encounters.

Anticipated Participant Responses:

- Learners may share personal experiences or express interest in further training.
- Some may ask for additional resources or raise questions about institutional support.
- Others may reflect on how the module changed their perspective on mental health care.

Follow-Up Questions:

- What’s one insight from today’s session that you’ll carry into your future practice?
- How can medical education better prepare learners to serve AANHPI populations?
- What additional tools or support would help you feel more confident in providing culturally responsive care?

Slide 47: **Post-Workshop Evaluation**

Facilitators should distribute the post-module evaluations and encourage participants to provide feedback.

Narration: “Please take a few minutes to complete the post-module evaluation. Your feedback is crucial in helping us improve this module for future sessions. Thank you.”
